# Genetic Architecture of Immune Cell DNA Methylation in the Rhesus Macaque

**DOI:** 10.1111/mec.17576

**Published:** 2024-11-24

**Authors:** Christina E. Costa, Marina M. Watowich, Elisabeth A. Goldman, Kirstin N. Sterner, Josue E. Negron-Del Valle, Daniel Phillips, Cayo Biobank Research Unit, Michael L. Platt, Michael J. Montague, Lauren J. N. Brent, James P. Higham, Noah Snyder-Mackler, Amanda J. Lea

**Affiliations:** 1 Department of Anthropology, New York University, New York, New York, USA; 2 New York Consortium in Evolutionary Primatology, New York, New York, USA; 3 Department of Biological Sciences, Vanderbilt University, Nashville, Tennessee, USA; 4 Department of Anthropology, University of Oregon, Eugene, Oregon, USA; 5 School of Life Sciences, Arizona State University, Tempe, Arizona, USA; 6 Center for Evolution and Medicine, Arizona State University, Tempe, Arizona, USA; 7 Department of Neuroscience, University of Pennsylvania, Philadelphia, Pennsylvania, USA; 8 Department of Psychology, University of Exeter, Exeter, UK; 9 School of Human Evolution and Social Change, Arizona State University, Tempe, Arizona, USA; 10 Neurodegenerative Disease Research Center, Arizona State University, Tempe, Arizona, USA

**Keywords:** epigenetics, gene regulation, immune function, primate, QTL

## Abstract

Genetic variation that impacts gene regulation, rather than protein function, can have strong effects on trait variation both within and between species. Epigenetic mechanisms, such as DNA methylation, are often an important intermediate link between genotype and phenotype, yet genetic effects on DNA methylation remain understudied in natural populations. To address this gap, we used reduced representation bisulfite sequencing to measure DNA methylation levels at 555,856 CpGs in peripheral whole blood of 573 samples collected from free-ranging rhesus macaques (*Macaca mulatta*) living on the island of Cayo Santiago, Puerto Rico. We used allele-specific methods to map *cis*-methylation quantitative trait loci (meQTL) and tested for effects of 243,389 single nucleotide polymorphisms (SNPs) on local DNA methylation levels. Of 776,092 tested SNP–CpG pairs, we identified 516,213 meQTL, with 69.12% of CpGs having at least one meQTL (FDR < 5%). On average, meQTL explained 21.2% of nearby methylation variance, significantly more than age or sex. meQTL were enriched in genomic compartments where methylation is likely to impact gene expression, for example, promoters, enhancers and binding sites for methylation-sensitive transcription factors. In support, using mRNA-seq data from 172 samples, we confirmed 332 meQTL as whole blood *cis*-expression QTL (eQTL) in the population, and found meQTL–eQTL genes were enriched for immune response functions, like antigen presentation and inflammation. Overall, our study takes an important step towards understanding the genetic architecture of DNA methylation in natural populations, and more generally points to the biological mechanisms driving phenotypic variation in our close relatives.

## 1 ∣ Introduction

A major goal in evolutionary biology is to understand genotype–phenotype relationships and the genetic architecture of complex traits. Non-coding changes that impact gene regulation, rather than protein function, are important contributors. In support of this, the majority of loci associated with complex traits and diseases in human genome-wide association studies (GWAS) fall in non-coding regions (Edwards et al. 2013; Schaub et al. 2012). Whilst protein-coding changes often have pleiotropic effects, regulatory changes may allow for more fine-tuned, targeted phenotypic changes, such as in specific tissues or cell types (Barbeira et al. 2018; GTEx Consortium 2017). For this reason, they have been hypothesized to play an outsized role in phenotypic expression and evolution (Haygood et al. 2010; Molodtsova et al. 2014; Wray 2007). For example, regulatory differences have long been hypothesized to underlie phenotypic differences between chimpanzees and humans (King and Wilson 1975), and recent evidence supports abundant *cis*-regulatory changes along the primate tree—particularly human-specific changes in brain cells (Agoglia et al. 2021; García-Pérez et al. 2021). Studies have also highlighted the significant regulatory contribution of introgressed Neanderthal alleles in modern populations (McCoy, Wakefield, and Akey 2017), and of modern-human specific variants (Weiss et al. 2021), especially those related to immune (Babbitt et al. 2017; Choate et al. 2021; Domínguez-Andrés et al. 2021) and morphological trait variation (Capellini et al. 2017; Gokhman et al. 2021; Prescott et al. 2015; Weiss et al. 2021).

To understand the genetic architecture of regulatory variation on a genome-wide scale, researchers have begun to map molecular quantitative trait loci (molQTL), defined as genetic variants that predict variation in molecular phenotypes (Aguet et al. 2023; Currin et al. 2021; McVicker et al. 2013). One such molecular phenotype is DNA methylation, which can be influenced by methylation quantitative trait loci (meQTL). DNA methylation is an essential component of mammalian gene regulation, often occurring at CpG dinucleotides where it can alter the expression levels of nearby genes (Lister et al. 2009; Wagner et al. 2014). Most DNA methylation patterns are mitotically inherited, established during early development to direct cell-type diversification and maintenance and largely stable across the lifespan (Delacher et al. 2017; Liang et al. 2011). However, a number of CpG sites can exhibit both transient and long-lasting changes in DNA methylation, for example during ageing and/or in response to environmental exposures (Baker et al. 2017; Laubach et al. 2019; Lea et al. 2016; Massart et al. 2014; Non et al. 2016; Provencal et al. 2012; Weaver et al. 2004).

While the epigenome is sensitive to development, ageing, and environmental exposures, it is also strongly impacted by local (i.e., ‘*cis*’) genetic variation. In support, epigenetic divergence closely recapitulates evolutionary divergence in primates and mammals (Haghani et al. 2023; Sahm et al. 2021; Vilgalys et al. 2019). Within species, most work has focused on mapping meQTL in human blood, where methylation levels are, on average, 18%–20% heritable (van Dongen et al. 2016), and sequence variation is a better predictor of methylation variation than demography or environment (Bergstedt et al. 2022; Czamara et al. 2021; Lea et al. 2017; Sepers et al. 2023). Where they occur, meQTL are thought to alter methylation via occupancy that prevents protein (e.g., transcription factor; TF) binding or that activates a cascade that includes the recruitment of methyltransferases (Villicaña and Bell 2021).

In humans, meQTL can be phenotypically meaningful: meQTL frequently co-localise with expression QTL (eQTL) as well as genetic associations with complex traits (Andrews et al. 2017; Gaunt et al. 2016; Pierce et al. 2018), such as cardiovascular disease (Huan et al. 2019), BMI (Hawe et al. 2022) and type 2 diabetes (Volkov et al. 2016). Not surprisingly given their phenotypic impact, there is also growing evidence for the adaptive potential of regulatory QTL. The *cis*-eQTL show signatures of selection for immune function between human populations (Kim-Hellmuth et al. 2017; Lea, Peng, and Ayroles 2022; Nédélec et al. 2016), and meQTL are implicated in adaptation to hypoxia in high altitude populations (Childebayeva et al. 2021) and stature in human hunter-gatherers (Fagny et al. 2015). Despite this, there has been little work in our close primate relatives, especially in natural populations, which has left a gap in our understanding of the evolutionary conservation of patterns of genotype-dependent gene regulation.

Here, we aimed to identify meQTL in a large population of free-ranging rhesus macaques (*Macaca mulatta*)—a leading biomedical research model exhibiting greater genetic diversity and functional variation than humans (Xue et al. 2016) but highly homologous physiology (Chiou et al. 2020; Cooper et al. 2022; Rhesus Macaque et al. 2007). To better understand how phenotypic variation is generated at the cellular level, we mapped genotype-methylation associations (*cis*-meQTL) in 573 whole blood samples using an approach that combines traditional meQTL mapping with the additional information that can be gained from considering allele-specific methylation (ASM). As we expect meQTL to impact functional epigenetic variation, we tested whether *cis*-meQTL: (1) have a greater effect on methylation variation compared to sex and age; (2) are enriched in active regulatory regions and (3) in vertebrate TF binding sites that are known to alter chromatin availability and DNA methylation. As regulatory QTL-associated loci are often less evolutionarily conserved (Shang et al. 2020; Tung et al. 2015), we also tested the prediction that meQTL are enriched (4) in regions showing differential methylation between macaque species. Finally, we hypothesized that meQTL-driven variation may also impact downstream phenotypes and tested the predictions that meQTL: (5) are enriched near genes actively regulated in macaque blood cells, such as immune genes; (6) are also whole blood *cis*-eQTL in the same population and (7) impact CpG sites where DNA methylation levels are correlated with gene expression levels.

## 2 ∣ Materials and Methods

### 2.1 ∣ Study Population

This study was conducted using samples collected from the rhesus macaque colony of Cayo Santiago, off the coast of Puerto Rico (Widdig et al. 2016). This population was established in 1938 with 409 founding macaques from India. It has been continuously researched since then, providing a wealth of information on each individual, including sex, age, relatedness, and many quantifiable social behaviours. As of 2023, it is home to over 1700 free-ranging macaques that form natural associations and social groups. There is no predation risk, and the animals are provisioned with food.

### 2.2 ∣ Reduced Representation Bisulfite Sequencing

**FIGURE 1 ∣:**
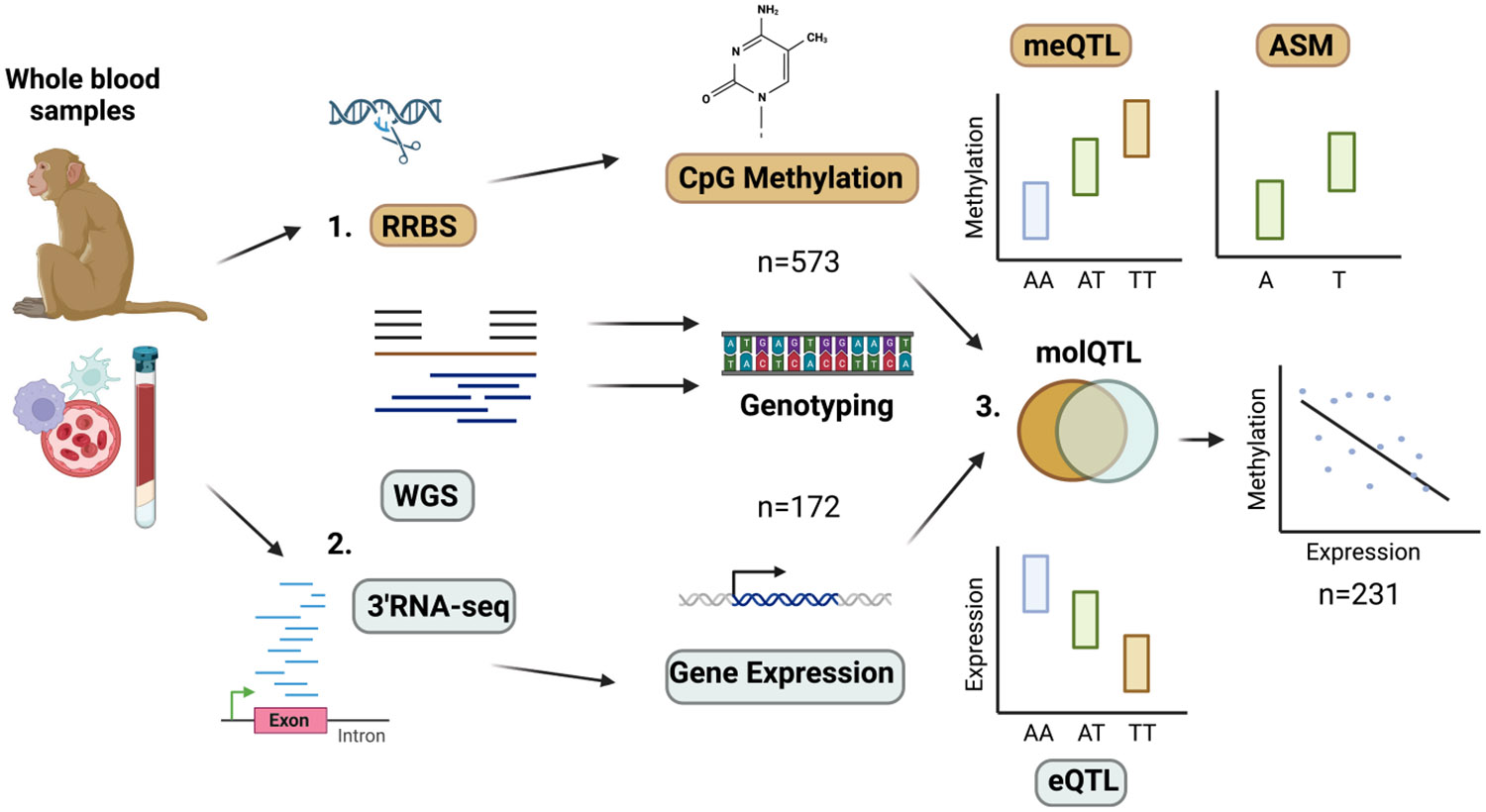
Data generation and analysis overview. DNA extracted from whole blood (1) Reduced representation bisulfite sequencing (RRBS), genotyping from RRBS reads, DNA methylation quantification and meQTL mapping. (2) 3′RNA sequencing and whole genome sequencing (WGS), genotyping from WGS reads, gene expression quantification and eQTL mapping. (3) Integration of meQTL and eQTL, and DNA methylation and gene expression levels. Numbers represent sample counts for each analysis. Dataset overlap information can be found in Figure S1. Figure 1 was created using BioRender.

CpG methylation was quantified from 573 peripheral whole blood samples (*n* = 500 unique individuals; Figure 1, Figure S1) collected between 2010 and 2018 in collaboration with the Caribbean Primate Research Center (CPRC). We sampled 68 individuals at least twice during the study (63 individuals sampled twice and five individuals sampled three times). The ratio of males to females was roughly balanced across collection years and ages (Figure S2), which range from infancy to late life (0.11–28.78 years; median lifespan: females 18, males 15). We extracted DNA from each blood sample (Qiagen Blood and Tissue DNA kit) and generated reduced representation bisulfite sequencing (RRBS) libraries to quantify DNA methylation using an established protocol (Snyder-Mackler 2022). RRBS is a cost-effective method for surveying DNA methylation levels that uses a methylation insensitive restriction enzyme (Msp1) which targets CpG-rich regions (Gu et al. 2011). This reduces the complexity of the genome and enriches functionally relevant sites near gene bodies (Lea et al. 2017; Figure S3). Samples were sequenced in two batches: the first batch was sequenced on the Illumina NovaSeq S2 flowcell (paired-end 50 bp reads), and the second on the NovaSeq S4 flowcell (paired-end 100 bp reads). Reads were trimmed to remove adapters and filtered for quality control. Subsequently, we mapped reads to the rhesus macaque reference genome (mmul_10; Warren et al. 2020) and quantified CpG-site specific DNA methylation using Bismark (v0.20.0) (Krueger and Andrews 2011).

### 2.3 ∣ Whole Genome Sequencing

We generated whole-genome sequencing (WGS) data from blood-derived DNA from 190 animals. Briefly, following DNA extraction, WGS libraries were generated using the Nextera DNA library preparation kit and sequenced using paired-end sequencing on the Illumina HiSeq 2500. Reads were mapped using bwa (Li and Durbin 2009). The average read depth was 7.13 (median = 2.04, SD = 14.13), and the average autosomal coverage was 71.06% (median = 67.32%, SD = 18.92%). For comparison with RRBS genotyping, genotypes were called using GATK’s best practice pipeline (Van der Auwera and O’Connor 2020), and the recommended germline hard filters were applied (Supporting Information Section 2.2). For the eQTL analysis, genotypes were imputed using the program ‘loimpute’ (Wasik et al. 2021) and the large rhesus macaque reference panel from the mGAP database (Bimber et al. 2019). This imputation pipeline has been shown to perform well even at low sequencing depth in this population (Watowich et al. 2023). SNPs were filtered for imputation genotype probability > 0.9, minor allele frequency (MAF) (> 0.05) and Hardy Weinberg equilibrium (HWE) (10e-8) using Plink v1.9 (Purcell et al. 2007). The final filtered dataset consisted of 5,684,471 SNPs (Figure S27).

### 2.4 ∣ RRBS Genotype Calling, ASM and Filtering

Genotype calls from bisulfite sequencing data can be inaccurate because unmethylated cytosines are converted to thymine following bisulfite conversion and PCR amplification, making it difficult to distinguish between thymine produced by bisulfite conversion and an actual thymine allele. We used the program CGmapTools, which is designed to call SNPs from bisulfite sequencing reads and implement a Bayesian wild card strategy (Guo et al. 2018). This method performs better than the majority of available tools for SNP calling from bisulfite data, showing the lowest number of false positives whilst maintaining relatively high true positives (Lindner et al. 2022). We used a conservative error rate of 0.01 and a dynamic *p*-value to account for per site read depth differences following (Fan et al. 2019). We used a modified version of the program which outputs homozygous reference genotype calls as well as heterozygous and homozygous alternate calls. The 573 bam files from Bismark were converted to the CGmapTools’ ATCGmap format for variant calling, and genotype calls were output to variant call files (VCF). Using BCFtools (V1.10.2) we removed sites with ambiguous genotype calls and with a read depth < 5. Additionally, only SNPs that were also present in the WGS dataset from the population were retained, to increase confidence. 3,951,189 SNPs were called in total from the RRBS dataset (Figures S4 and S5). For the filtered variants an average of 613,770 SNPs were genotyped per individual (median = 579,547, SD = 206,103.50). The average number of reads per SNP–CpG pair for each genotype and methylation call was 18.39 (median = 13.92, SD = 55.24). For samples with both RRBS and WGS data (*n* = 163, Figure S1), genotypes for all sites that could be compared (mean = 32,798.18) showed > 80% agreement on average, which increased to > 90% agreement when limiting to only homozygous sites (Supporting Information Section 2.2; Figure S6).

Allele-specific methylation can be detected in sequencing data from heterozygous individuals when differences in methylation levels are detected between the two alleles. CGmapTools was used to obtain ASM estimates from the RRBS data, taking the VCF with genotypes and the bams as input. As variants were called directly from the RRBS data, SNP–CpG pairs in this analysis come from the same read, allowing us to test only proximal *cis*-genetic effects (within 100 bp) (Figure S9). The output ASM files contain genotype and methylation level information for sites both with and without ASM. These SNP–CpG pairs were filtered and served as input for IMAGE meQTL analysis. We removed CpGs in the dataset: (i) that were measured in less than 10% of samples; (ii) that were constitutively hypomethylated or hypermethylated, that is, where methylation levels fell below 10% or above 90% in at least 90% of measured individuals (Figure S7); (iii) that had a mean read depth < 5 or (iv) that were paired with an SNP with MAF < 0.05 across individuals with DNA methylation estimates. These filters largely followed Fan et al. (2019). The final dataset included 776,092 autosomal SNP–CpG pairs (555,856 unique CpGs, 243,389 unique SNPs; mean SNPs per CpG = 1.40, median = 1, max = 28).

### 2.5 ∣ Gene Expression Levels (mRNA Sequencing)

We used a previously published gene expression dataset that intersects with our study individuals (Figure S1). Transcript levels of 547 whole blood samples (*n* = 445 unique individuals) were quantified using 3′ mRNA-seq (Pallares, Picard, and Ayroles 2020). Data generation and processing are outlined in (Watowich et al. 2022). Briefly, peripheral whole blood samples were collected from 2013 to 2018 in a PAXgene Blood RNA Tube prior to extraction and library preparation. Libraries were sequenced on an Illumina NovaSeq S2 flowcell, reads mapped to the macaque reference genome (mmul_10) and read counts normalised. After filtering, 7009 expressed genes were retained.

### 2.6 ∣ Genetic Associations With DNA Methylation Levels

To identify *cis*-meQTLs in our 573 samples, we used the R software program IMAGE to model associations between 776,092 SNP–CpGs (Fan et al. 2019). IMAGE has greater power to detect genetic associations with nearby methylation levels compared to similar methods as it jointly models differential methylation across genotypes at biallelic sites, and information on ASM when available (Figure 1). IMAGE employs an overdispersed binomial mixed model, shown to be the most robust way to model the count-based nature of bisulfite sequencing data in the presence of relatives or genetic structure (Lea, Tung, and Zhou 2015). Sequencing batch, sex and age were included as fixed effects covariates in the model. We controlled for relatedness and repeated measurements of the same individual by including a random effect derived from a genetic covariance matrix. Pairwise relatedness estimates were obtained from the population pedigree, and repeated measurements for the same individual were denoted by a value of 1 (as in Cuomo et al. 2021; see also Supporting Information Section 2.3). Mixed modelling approaches that rely on a genetic covariance matrix to control for population structure, familial relatedness and/or repeated measurements are well-established and used heavily in the fields of quantitative, population and functional genomics (Johnston et al. 2021; Kang et al. 2008; Lea, Tung, and Zhou 2015; Snyder-Mackler et al. 2019; Sul, Martin, and Eskin 2018; Sun et al. 2019; Zhou and Stephens 2012). Applying mixed effects models to control for the use of repeated, longitudinally collected measurements has been successful in similar study designs to our own (Eu-Ahsunthornwattana, et al. 2014; Watowich et al. 2022).

We used a permutation-based multiple testing correction method. Specifically, we used the built-in permutation function in IMAGE (*n* = 10 per chromosome). The permuted and actual *p*-values were input into the empirical null-based multiple testing correction R program ‘perm.fdr’, which uses the Storey–Tibshirani method (Storey and Tibshirani 2003) to provide corresponding *q*-values for a list of *p*-values (Figure S10). We used a false discovery rate (FDR) of 5%. IMAGE models genotype residuals and does not output covariate estimates. We calculated the percent variance explained (PVE) by genotype for each SNP–CpG pair using residuals from linear models with all covariates, and beta estimates for genotype from IMAGE (Supporting Information Section 2.3).

To compare demographic and local genetic contributions to interindividual methylation variation on a site-by-site basis we used a modelling approach in the R package ‘PQLseq’, which outputs estimates for all covariates. We modelled the same 776,092 SNP–CpG pairs, using the same methylation and genotype data as input. PQLseq uses a binomial mixed model similar to that employed by IMAGE, but without information on ASM (Sun et al. 2019). We therefore expected it to be underpowered compared to our primary modelling approach. A total of 709,049 pairs were successfully modelled in PQLseq, 630,826 of which converged. We again modelled methylation as a function of genotype at the associated SNP, whilst controlling for sex, age, sequencing batch and genetic relatedness. PVE was calculated for each predictor (Supporting Information Section 2.3; Figure S15). PVE estimates and figures are filtered for SNP–CpG pairs with sigma^2^ values greater than zero (Supporting Information Section 3; Figures S20-S26). In addition, we carried out meQTL mapping with only unique individuals (*n* = 500); 80.07% of SNP–CpGs significant in the larger dataset remained significant at the same threshold (5% FDR), and effect sizes were highly correlated (rho = 0.886, *p* < 2.2e-16; Supporting Information Section 2.4, Figure S14).

### 2.7 ∣ Genetic Associations With Gene Expression Levels

We used the program GEMMA (Zhou and Stephens 2012) to implement a linear mixed model to test for associations between genotype at significant meQTL SNPs and gene expression levels in the whole blood RNA-seq data. A total of 172 samples (*n* = 120 unique individuals) overlap with the WGS data (Figure 1) and were used for eQTL calling (Figure 1). We used imputed genotypes, as is recommended for modelling associations in GEMMA, with the default setting excluding sites with > 5% genotype missingness. To increase our ability to detect genetic effects on expression, we performed a surrogate variable analysis (SVA) using the R package ‘sva’ (Leek and Storey 2007) on the normalised expression data whilst preserving variation due to age and sex, a common practice in eQTL mapping (T. Wang et al. 2021). We took the residuals of the fitted linear model including the first 5 SVs for each normalised gene count and used these as the dependent variable for the eQTL analysis. We chose 5 SV because it showed the greatest increase in eQTL gene detection when comparing models including 1–15 SV (Figure S28). We tested for associations between genotype and gene expression of all SNPs within 200 kb of a gene in the whole blood dataset (2,062,839 SNPs, 6412 genes and 4,836,446 SNP–gene pairs). SNPs can map to multiple genes (2.35 on average). The R package ‘qvalue’ was used to control for multiple testing, and an FDR of 20% was used to account for reduced power in the eQTL compared to the meQTL analysis (Supporting Information Section 4.3, Figures S29 and S30). Intercept, sex, age and RIN (RNA integrity number) were included as covariates, and a random effect from the relatedness matrix generated from the whole genome SNP genotypes was included to account for relatedness, and repeated individuals (see Supporting Information Section 2.3 for further explanation of the mixed modelling approach). The eQTL analysis was also conducted with unique individuals (*n* = 120). 34.98% of SNP–gene pairs significant in the larger dataset remained significant at the same threshold (20% FDR), and effect sizes were highly correlated (rho = 0.976, *p* < 2.2e-16. Supporting Information Section 4.2, Figure S31).

### 2.8 ∣ Annotations and Enrichment Analyses

Gene feature annotations were created from the macaque (mmul_10) GTF file from Ensembl. Transcription start sites (TSS) were labelled as the start or end of the gene depending on if it was on the positive or negative strand. Promoters were defined as the 2 kb region upstream of the TSS. A site was in an intron or untranslated region of a gene if it was within a gene body but not in an exon. Macaque CpG islands were downloaded from the UCSC Genome Browser. CpG shores were labelled as 2 kb from the start and end of a CpG island, and CpG shelves were 2 kb from the start or end of a CpG shore. Any sites not in a CpG island, shore or shelf, were considered to be in an open sea (Figure S17). Open chromatin annotations were pulled from ATAC-seq peaks called from untreated peripheral blood mononuclear cells (PBMCs) from 43 captive female macaques (Snyder-Mackler et al. 2019). We also drew on chromatin activity state information from the Human Epigenetic Roadmap project (Roadmap Epigenomics Consortium et al. 2015): we used the 15-chromatin state model generated in human PBMCs (E062 bed file) and converted the annotations from human (hg38) to rhesus macaque (mmul_10) genome coordinates using the UCSC genome browser program LiftOver (Kuhn, Haussler, and Kent 2013). We expect chromatin patterns to be highly conserved within species, where reference individuals are often used to annotate population-level analyses, as well as across closely related species (Lea et al. 2016; García-Pérez et al. 2021). meQTL SNPs in SNP–CpG pairs were labelled as residing within their associated CpG if they were within 1 base pair (CpG chromosomal position +1) following Fan et al. 2019. Differentially methylated regions (DMRs) between macaque species were obtained from Wang et al. (2023). Finally, meQTL (5% FDR) and eQTL (20% FDR) SNPs were considered overlapping (meQTL–eQTL) if they shared the same genomic position. We used one-tailed Fisher’s exact tests to test for enrichment of genotype-dependent methylation in functional regions of the genome (Figure S17).

We used HOMER (v4.11) to test for the enrichment of TF motifs surrounding meQTL SNPs (Heinz et al. 2010). We created 200 bp regions, encompassing 100 bp downstream and upstream of each SNP (*n* = 185,698). The background set was all SNPs in the IMAGE analysis. HOMER then takes nonoverlapping input and background regions as the background (*n* = 31,347). We tested for enrichment of 441 known vertebrate TF motifs, controlling for CpG% in the input regions, on top of the automatic normalisation HOMER performs to control for sequence bias. HOMER performs a Benjamini–Hochberg *p*-value correction and provides *q*-values for each motif. The same analysis was carried out for meQTL–eQTL SNPs (S4. Table 1).

A list of macaque genes harbouring at least one meQTL is not biologically informative, given the high number of macaque protein-coding genes that had a nearby meQTL (similar to high-powered human molQTL studies (GTEx Consortium 2017)). Instead, we asked which genes harboured the highest number of significant meQTL CpGs, whilst controlling for differences in the number of CpGs tested for each gene. For all genes with at least 10 tested CpG sites within 50 kb of its coding region, we calculated the proportion of significant meQTL CpGs out of the total number of tested CpGs (Figure S18). To determine which genes were harbouring more or less meQTL CpGs than expected by chance, we took a permutation approach and permuted the significance level of all CpGs in the dataset (*n* = 100). Protein-coding genes with proportions greater than the 95th or less than the 5th percentile of the permuted distribution were input to the Gene Ontology (GO) enrichment program ShinyGO (v0.77) (Ge, Jung, and Yao 2020), using all protein-coding genes with at least 10 tested CpGs as the background set (*n* = 10,415 genes; Table S1.9). FDR is calculated based on the *p*-value from the hypergeometric test, and for this analysis we used a relaxed 20% threshold. eGenes with a meQTL–eQTL SNP were also input to ShinyGO. Background genes were genes expressed in the whole blood dataset with a meQTL SNP within 200 kb of the TSS (*n* = 6100; Tables S2.5).

### 2.9 ∣ Correlation of meQTL–eQTL CpG Methylation and Gene Expression

Spearman’s rank correlation (rho) was calculated for all meQTL–eQTL CpG–Gene pairs (*n* = 908) to assess the degree of correlation between CpG methylation and gene expression levels at overlapping QTL. This was done using individual DNA methylation and expression measurements from 231 matched RRBS and mRNA-seq data generated from the same blood samples (*n* = 226 unique individuals; Figure 1, Figure S1). The R package ‘qvalue’ was used for multiple testing corrections (Storey and Tibshirani 2003), and here we again used a relaxed 20% FDR threshold. All statistical analyses were conducted using R version 4.0.2.

## 3 ∣ Results

### 3.1 ∣ Identification of *cis*-meQTL in a Large Sample of Rhesus Macaques

**FIGURE 2 ∣:**
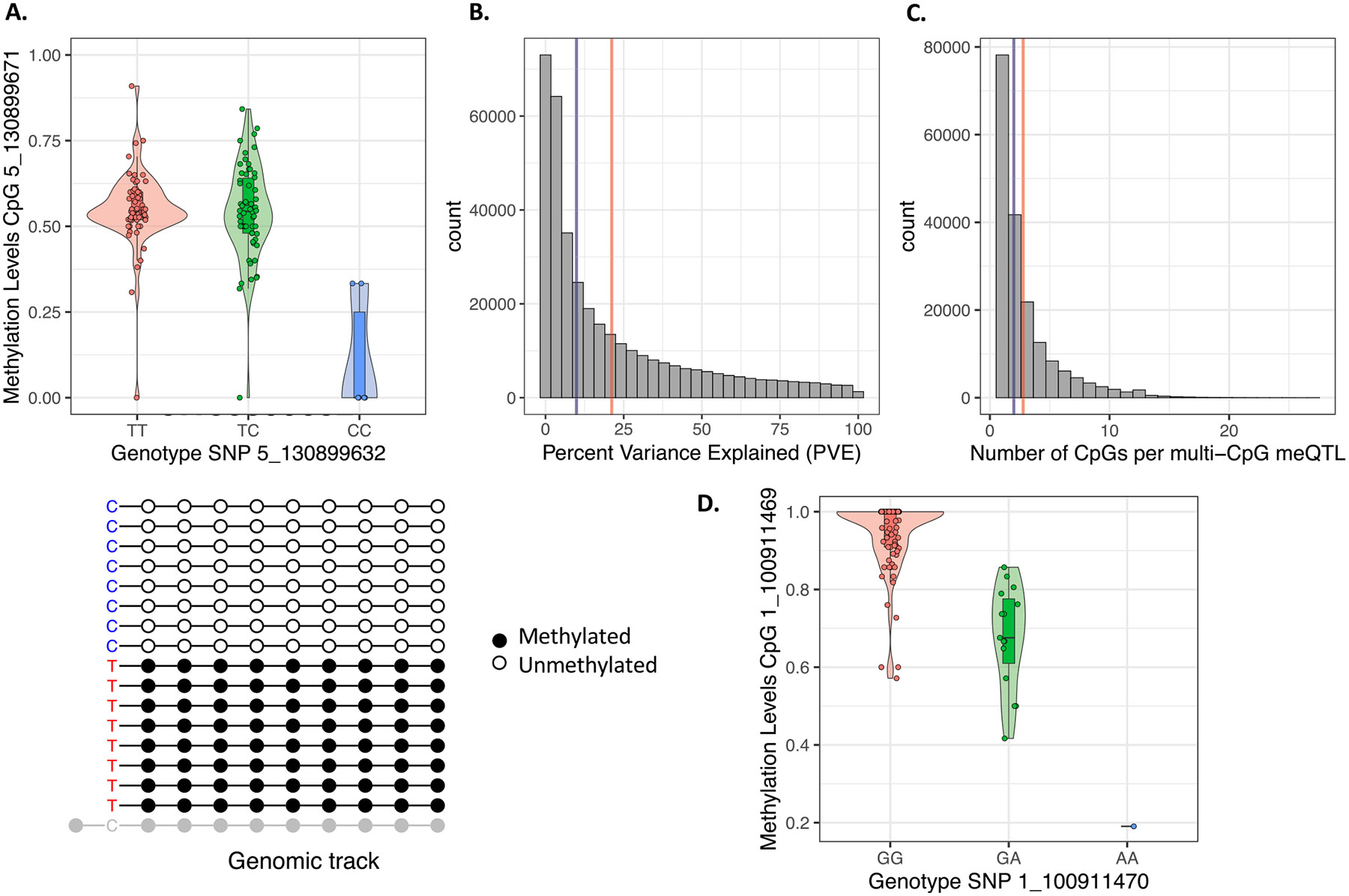
Characteristics of significant whole blood *cis* meQTL. (A). meQTL with allele-specific methylation (ASM). (*Top*) Genotype-methylation association between meQTL chr5 pos 130899632 and one nearby CpG (Counted = C, Alt = T) (Ref = C, Non-ref = T). Beta = 4.09 (*Below*) Allele-specific plot of methylated reads (total = 16) in a single heterozygous sample for the same SNP (chr5 pos 130899632). (B). Distribution of percent methylation variance explained (PVE) by meQTL genotype for each significant SNP–CpG pair. Blue line = Median (9.9%); Red line = mean (21.2%). (C). Histogram of the number of CpGs associated with meQTL with more than one significant CpG. Blue line = Median (2), Red line = mean (2.8), maximum = 27. (D). Example of a CpG disrupting SNP on chromosome 1.

The majority of SNP–CpG DNA methylation level pairs (66.5%) were significantly associated with one another (*n* = 516,213 whole blood *cis*-meQTLs; permutation-based FDR < 5%). This included 185,739 unique SNPs and 384,211 unique CpGs (Figure 2A). The average percent variance in DNA methylation levels explained (PVE) by the genotype of each meQTL was 21.2% (median = 9.9%, SD = 25.1%) (Figure 2B). The majority of meQTL SNPs (57.9%) were associated with more than one CpG (Figure 2C), suggesting strong and concordant genetic control of methylation at a regional level. Indeed, if an SNP was a meQTL for multiple CpG sites, it almost always had a concordant directional effect for nearby CpGs (92.6% of multi-CpG meQTL had an effect on 2 or more associated CpGs in the same direction). Methylation was significantly associated with variation at more than one nearby SNP for 92,292 CpGs (24.0%), suggesting potential additive or linked effects of meQTL on methylation at those sites.

As expected, power to detect a meQTL scaled with MAF of the SNP. Furthermore, meQTL CpGs show intermediate average methylation levels, and greater methylation variation across samples compared to non-meQTL CpGs (Figure S11). 4.06% (*n* = 20,938) of meQTL CpGs are disrupted by their SNP (e.g., Figure 2D), compared to only 0.69% (*n* = 1780) of non-meQTL CpGs (Fisher’s exact test *p* < 2.2e-16, log2OR = 2.616; Figure S12). The majority of meQTL were replicated in the PQLseq modelling framework, and the effect sizes are highly correlated for sites where the non-reference allele is the minor allele (rho = 0.795, *p* < 2.2e-16; Supporting Information Section 2.6, Figure S13).

### 3.2 ∣ Genotype Explains More DNAm Variation Than Age or Sex

**FIGURE 3 ∣:**
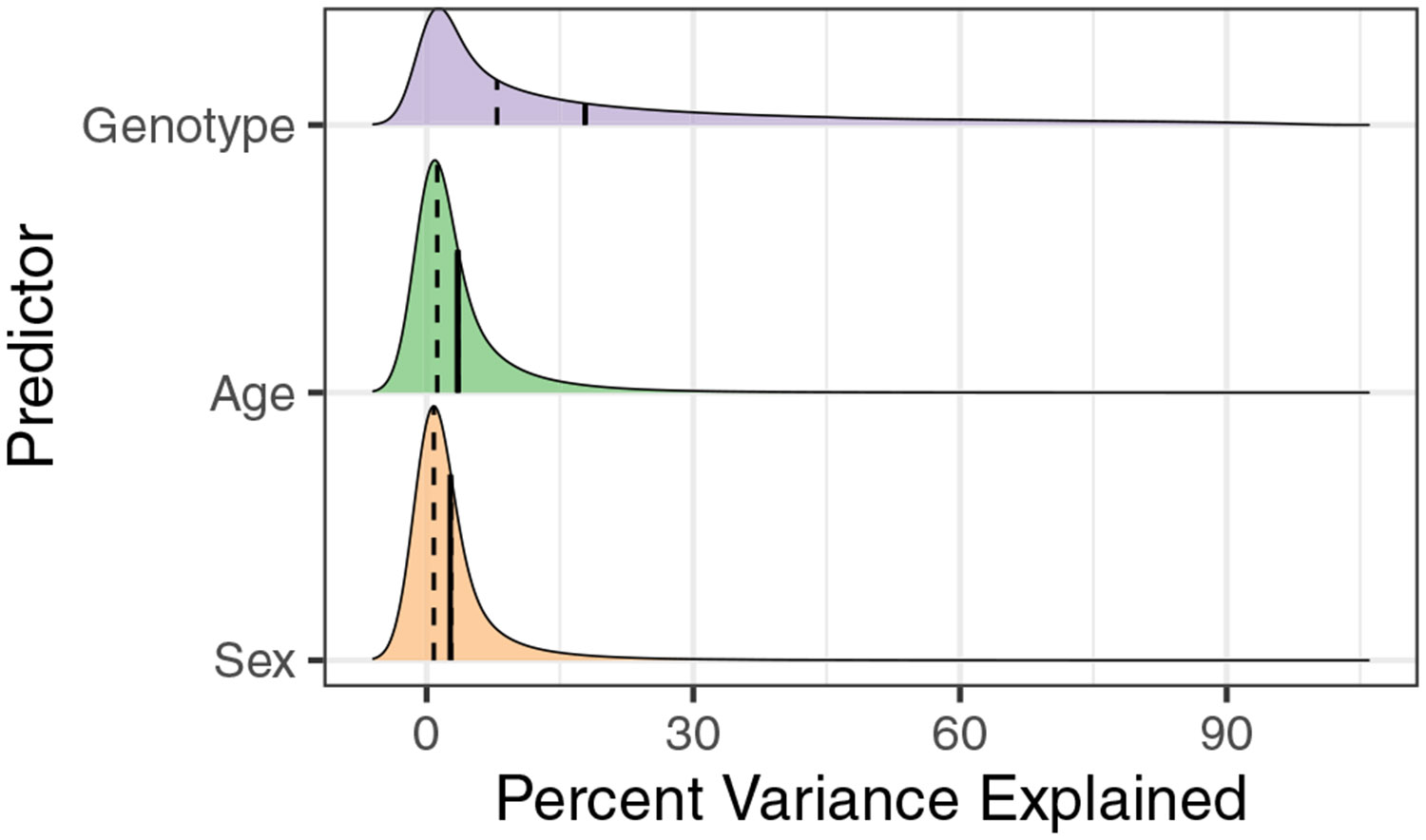
Genotype is a strong predictor of methylation variation. Density plot of percent variance explained (PVE) by predictor for all CpGs. Dotted lines represent the median and solid black lines represent the average PVE of the predictor. Average PVE explained by associated SNP genotype versus age and sex (*t*-test, *p* < 2.2e-16). *n* = 447,453 SNP–CpGs (filtered for pairs with sigma^2^ > 0; see Supporting Information Section 3.1).

To determine the relative contribution of genotype, sex and age to DNA methylation (DNAm) variation in the macaques, we also tested for demographic effects and calculated the PVE by each variable (genotype, sex or age). Average PVE by genotype was significantly greater (*t*-test, *p* < 2.2e-16, mean = 17.8%, median = 7.9%, SD = 22.4%) than demographic effects on methylation at the same sites (age = 3.55%, sex = 2.75%) (Figure 3).

At the site level, 4004 CpGs had significant age effects and 87 had significant sex effects (5% FDR). The majority (70.73%) of age-associated CpGs decreased with age, and the majority (79.46%) of sex-associated CpGs showed higher methylation in females compared to males. CpGs with significant age (Fisher’s exact; log2OR = 0.677, *p* < 2.2e-16) and sex (Fisher’s exact; log2OR = 1.852, *p* = 1.24e-07) effects were both more often than not also associated with a meQTL, regardless of average methylation level (Table S1.14).

### 3.3 ∣ meQTL CpGs Are Enriched in Active Regulatory Regions

**FIGURE 4 ∣:**
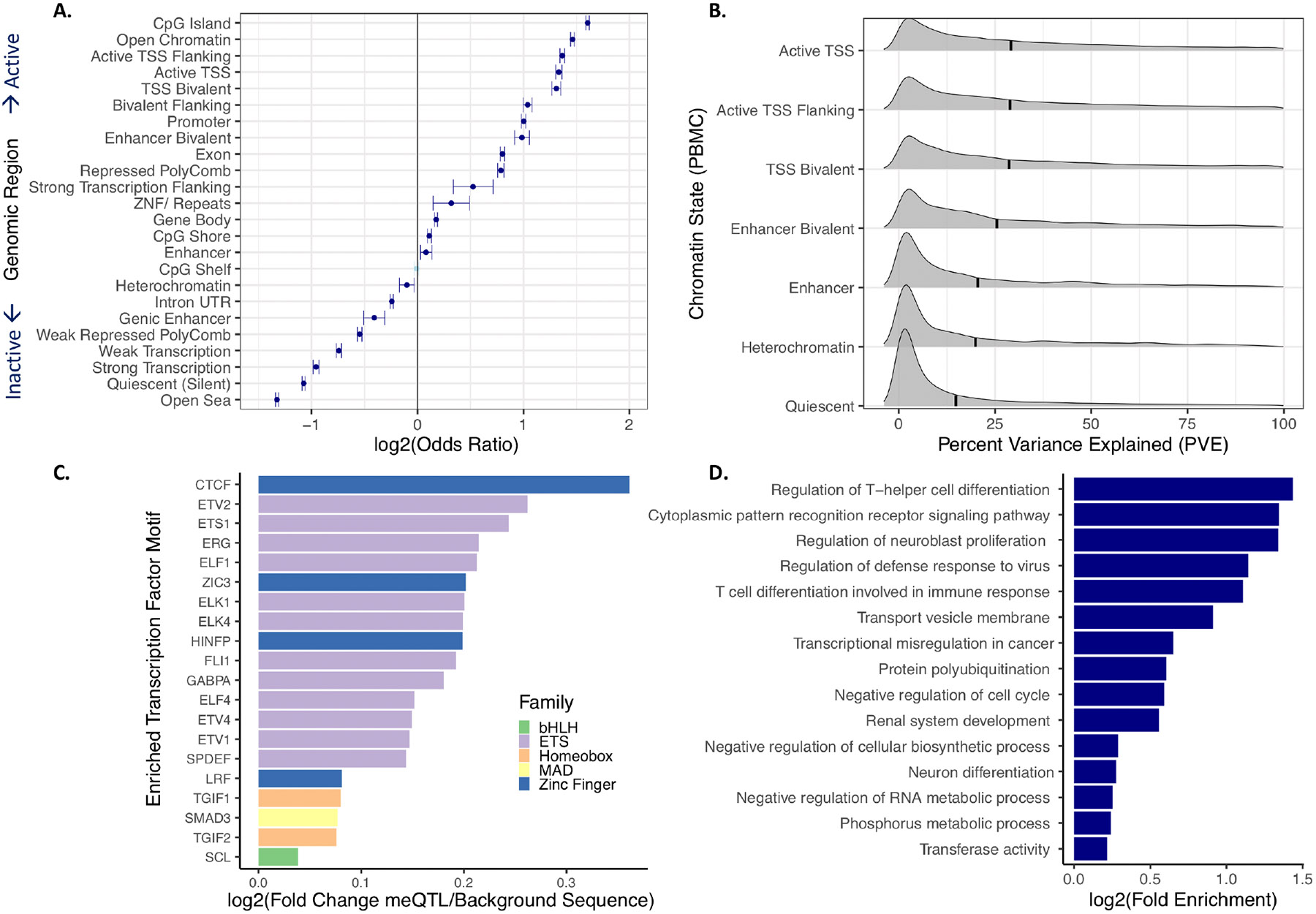
meQTL are enriched in functional regions of the genome. (A). Enrichment of meQTL CpGs compared to non-meQTL CpGs in genomic contexts and chromatin states. All chromatin state annotations are from human PBMCs, except ‘Open Chromatin’, measured in macaque PBMCs. Regions and chromatin states are not mutually exclusive. Points represent log2(OR) with 95% confidence interval. Positive values represent enrichment and negative represent depletion of CpGs in that region. Fisher’s Exact Test *p* < 2.20E-16 for all regions except the CpG shelf (Table S1.6). (B). Average percent methylation explained (PVE) by meQTL genotype (black line) is greater in active chromatin states (*p* < 2.2e-16, *t*-test: Active TSS, Active Flanking, Bivalent, vs. Quiescent). (C). Enrichment of top TF binding site motifs near *cis* meQTL compared to non-meQTL regions. Input sequences = 185,698, background = 31,347. Includes TF motifs with *q* < 0.05, > 5% of target sequences with motif and > 1% difference between target and background sequence. *CTCF* is also highlighted, although < 5% of targets had this motif. Colours represent the motif TF family. *x*-axis: log2 fold change in percent motifs found in meQTL vs. non-meQTL sequences. (D). meQTL-associated genes are involved in regulatory and immune pathways. FDR 20%. *y*-axis: Gene ontology pathway term. *x*-axis: log2 fold enrichment for meQTL genes compared to all genes with tested CpGs in that pathway.

After confirming widespread genotype-dependent methylation, we wanted to ask how these effects were distributed across the genome, to test the prediction that they would be enriched for functionally relevant CpGs. We found multiple lines of evidence suggesting that meQTL CpG sites were enriched in regulatory compartments. meQTL CpGs (FDR < 0.05) are more likely than non-meQTL CpGs to be found in CpG islands (Fisher’s exact test *p* < 2.20E-16, log2OR = 1.604) and CpG shores (Fisher’s exact test *p* < 2.20E-16, log2OR = 0.111), and less likely to be found in open seas (Fisher’s exact *p* < 2.20E-16, log2OR = −1.326) (Figure 4A). They are also more likely to be found in promoters (*p* < 2.20E-16, log2OR = 1.002) and exons (*p* < 2.20E-16, log2OR = 0.802), and less likely to be found in the untranslated regions (introns and UTRs) (*p* < 2.20E-16, log2OR = −0.243) (Figure 4A).

The whole-blood meQTL CpGs were enriched in accessible chromatin regions characterised in macaque PBMCs, which are likely to be active regulatory regions in this tissue (*p* < 2.20E-16, log2OR = 1.463) (Figure 4A). meQTL are also more likely to be found in active chromatin states as indicated by chromatin activity annotations in human PBMCs (Figure 4A, Table S1.6). For example, meQTL CpGs are enriched near active TSSs, bivalent states and enhancers. They are depleted in quiescent (silent) states and regions of heterochromatin (inaccessible regions). meQTL SNPs in active regulatory regions explained more methylation variation on average when compared to those in silent or heterochromatic regions, consistent with their greater phenotypic impact (Figure 4B, Figure S16). This is not due to lower methylation variation in active regions, which are actually more variable on average (Figure S8).

### 3.4 ∣ meQTL SNPs Are Enriched in TF Binding Sites

After confirming that meQTL were enriched in active regulatory regions of the genome, we next tested the hypothesis that these variants impact methylation through the disruption of nearby TF binding sites. As predicted, we found that meQTL SNPs (*n* = 185,698) were more likely than non-meQTL SNPs (*n* = 31,347) to fall in or near known vertebrate TF binding sites (*n* = 245 enriched motifs; FDR < 5%, Table S1.5). This included 2930 meQTL regions (SNP ± 100 bp) enriched for binding sites of the chromatin regulator *CTCF*, where altered binding affinity has been linked to methylation variation (Banovich et al. 2014). The majority of the top enriched motifs (63.2%) belonged to the ETS family of TFs. The second most enriched were Zinc Fingers (ZF) (15.8%), followed by Homeobox (10.5%), MAD (5.3%) and bHLH (5.3%) (Figure 4C).

### 3.5 ∣ meQTL CpGs Are Enriched in Regions Showing Differential Methylation Between Species

Regulatory-associated loci can vary in their expression both within and between populations and species and may represent less conserved regulatory regions. In other words, they may be under less constraint at both the sequence and regulatory level, contributing to increased levels of variation within and between populations (Fair et al. 2020). In some cases, DNA methylation differences between populations may be explained by differences in allele frequencies at loci that also affect within-population variation. We therefore asked whether rhesus macaque meQTL CpGs are enriched in DMRs identified between three macaque species (Rhesus vs. Tibetan (*Macaca thibetana*), Rhesus versus Crab-eating (*Macaca fascicularis*), Tibetan versus Crab-eating; Wang et al. 2023). We found meQTL CpGs are 2.64 times more likely to fall in a DMR for any one of the species comparisons than CpGs without a significant meQTL (log2OR = 1.40, log2lowerCI = 1.37, log2upperCI = 1.43, *p* < 2.2e-16).

### 3.6 ∣ meQTL-Associated Genes Are Enriched in Immune Response and Regulatory Pathways

We next asked whether there were particular sets of genes that are more likely to harbour higher numbers of significant meQTL. We found that genes with high numbers of meQTL (*n* = 2667; Table S1.3) are enriched in pathways related to key cellular, metabolic, regulatory and immune functions (Figure 4D, Table S1.2). This includes genes responsible for the negative regulation of cellular processes, cell proliferation and protein modification. There is also an enrichment for transferase activity, including methyltransferases like *DNMT3B*, and MAPK (mitogen-activated protein kinase) genes, like *MAPK11*. The greatest fold change was seen in immune response pathways, including T cell differentiation, and cytoplasmic pattern recognition receptor signalling, an important component of the innate immune system (Li and Wu 2021). This includes interleukin receptor *IL18R1*, TFs like *RELA*, an NF-kappa-B subunit and *ZBTB7B*, which regulates T cell lineages, and genes that promote receptor-mediated signalling like *DDX60*. A gene set enrichment analysis (GSEA) recapitulated many of these results and revealed that meQTL-associated genes are enriched for differential expression in stimulated immune cells, for example in dendritic cells exposed to lipopolysaccharide and naive versus memory CD8 T cells (Supporting Information Section 2.7; Table S1.10-13). Genes less likely to harbour meQTL (*n* = 1533; Table S1.7) are enriched for regulation of transposition in the genome (Table S1.8, Figure S19).

### 3.7 ∣ meQTL Overlap Whole Blood Expression Quantitative Trait Loci (*cis*-eQTL)

**FIGURE 5 ∣:**
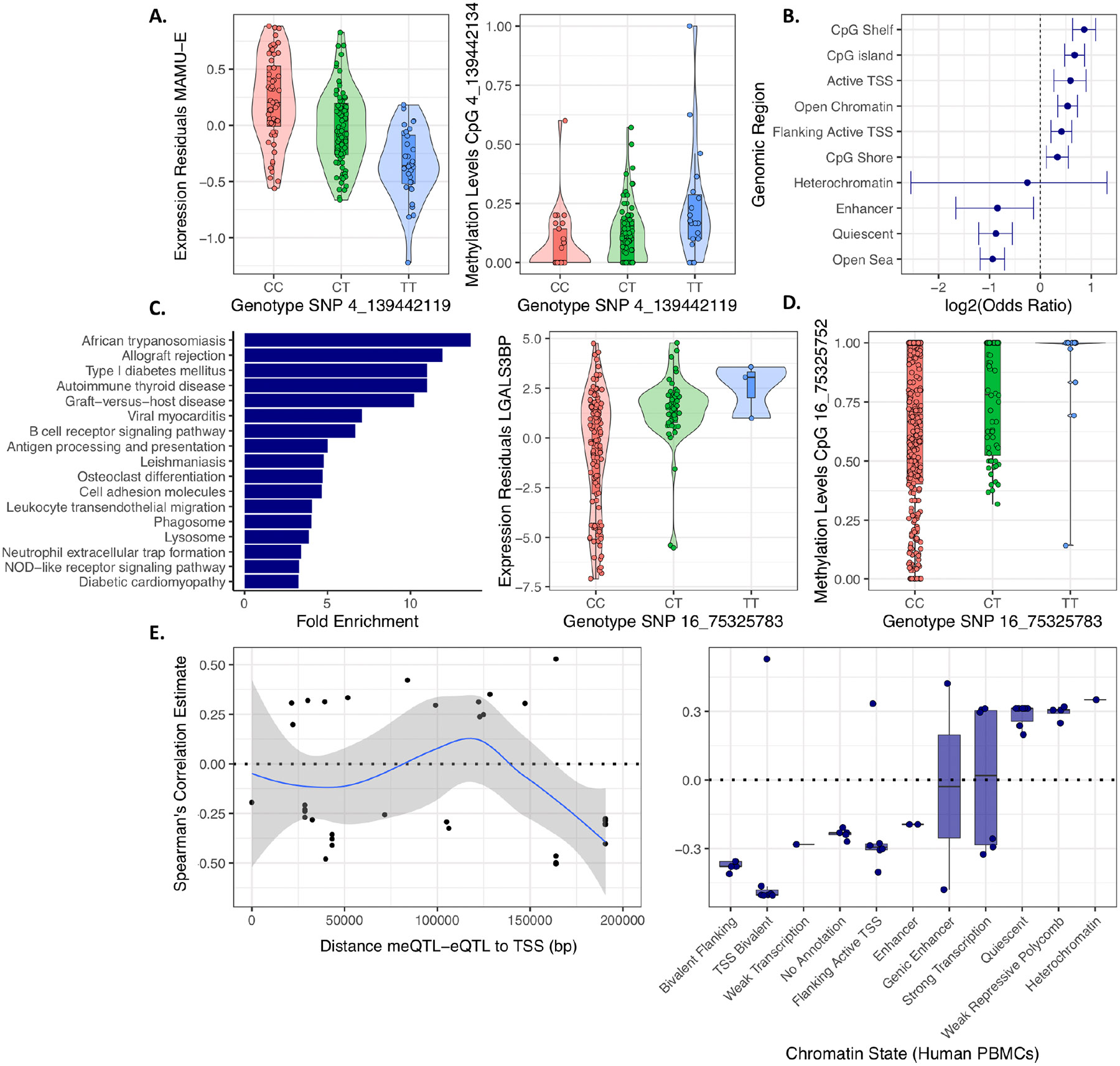
meQTL overlap eQTL in active regulatory regions of immune genes and show coordinated changes in methylation-expression levels. (A). Example meQTL–eQTL associated with *MAMU-E* expression (*left*) and methylation (*right*). (B). Enrichment of meQTL–eQTL CpGs in different genomic regions. Error bars represent 95% confidence intervals. (C). Gene ontology (GO) enrichment of macaque meQTL–eQTL eGenes. FDR 20%. *y*-axis: GO term. *x*-axis: Fold enrichment meQTL–eQTL eGenes compared to tested genes. (D). (*left*) eQTL for *LGALS3BP* (beta = 1.879) and (*right*) meQTL for correlated CpG (PQLseq beta = 0.613), Rho = 0.307. Chromatin state: strong transcription, Region: open sea. (E). Spearman’s methylation-expression correlation estimate against QTL distance from gene TSS (*left*) and by chromatin state (*right*). *y*-axis: Spearman’s rho.

To test the hypothesis that meQTL also influences nearby gene regulation, we first tested for associations between genotype and expression levels of 4,836,446 SNP–gene pairs. There were 18,398 significant eQTL (FDR 20%), including 17,533 unique SNPs and 619 genes (eGenes) (Table S2.10-11). Of the 26,816 whole blood *cis*-meQTL within 200 kb of a gene in the whole blood RNAseq dataset (90,135 SNP–gene pairs), 332 were also identified as a *cis*-eQTL (e.g., Figure 5A). In other words, the genotype at that SNP was associated with both local methylation and gene expression levels. 20.87% of eGenes had at least one meQTL–eQTL, for a total of 129 meQTL–eQTL genes, 345 SNP–gene pairs and 966 unique meQTL–eQTL SNP–CpG–Gene trios (Table S2.1). The effect of QTL genotype on methylation and expression is in the same direction 56.4% of the time, which is modest but more than expected by chance (binomial *p* = 0.0002). This relationship is not dependent on the distance of the QTL to the gene TSS (Figures S33-S35). meQTL–eQTL CpGs (*n* = 924) are further enriched in active regions, compared to non-eQTL meQTL CpGs tested (*n* = 67,333) (Figure 5B, Figure S32). eGenes with a meQTL–eQTL, compared to all expressed genes with nearby meQTL, were enriched for immune functions (Figure 5C, Table S2.2-4). This included antigen presentation and signalling genes, like MHC (major histocompatibility complex) class I gene *MAMU-E*, *CD81*, involved in B cell receptor signalling, and *LILRA5* and *LILRA3*, with innate immune and pro-inflammatory functions. Enrichment of NOD-like receptor signalling, a type of cytoplasmic recognition receptor, mirrors results for genes with many meQTL, for example *MYD88*, an immune signalling protein in the TLR4 pathway.

After identifying QTL–CpG–Gene trios we wanted to assess the functional relationship between CpGs and genes under shared genetic control. Using matched methylation and gene expression data, 38 meQTL–eQTL CpG–Gene pairs (4.19%) were significantly correlated (FDR 20%; Table S2.8-9). 12.4% of meQTL–eQTL eGenes had at least one CpG where methylation correlated with its expression (e.g., Figure 5D, Figure S37). The average correlation estimate (absolute value) was 0.339 (median = 0.310, SD = 0.099). The majority (65.79%; *n* = 25) of CpG–Gene pairs showed the canonical negative correlation, and are found in CpG islands, some of which fall in promoters and near active or bivalent TSSs. Positive correlations were found in silent states, open seas and gene bodies (Figure 5E, Figures S36 and S38).

If the QTL influences expression through its impact on methylation, we would predict that methylation and expression levels at those loci would be correlated, as in these pairs, and that the direction of the QTL effect would be consistent with the correlation (i.e., a negative correlation and opposite QTL effect). For significant pairs, the correlation estimate agrees with the direction of the QTL effect on methylation and expression 37.9% of the time (Figure S39). This suggests evidence of a shared causal mechanism, but also the potential that some QTL exert independent effects on methylation and gene expression. Many correlated genes are immune response genes, like the MHC class II gene *Mamu-DPA1* involved in antigen processing. The most significant methylation-expression associations included *LGALS3BP*, involved in intercellular interactions, cell cytotoxicity and upregulated during cancer and HIV infection in humans (Capone, Iacobelli, and Sala 2021; Lodermeyer et al. 2013), although its antiviral properties may not be fully conserved in macaques (Lodermeyer et al. 2018). The strongest CpG–gene correlations (> 0.5) are in the regulatory region of *FCGR2A*, a cell surface receptor, involved in phagocytosis and immune complex clearing.

## 4 ∣ Discussion

We comprehensively mapped *cis*-meQTLs and *cis*-eQTLs in a large dataset of free-ranging macaques and identified genome-wide patterns of genetic effects on gene regulation. meQTL have large effects on nearby DNA methylation, are frequently found in regulatory regions, TF binding sites, and near immune response genes, and in some cases also correlate with gene expression levels, at the variant and methylation level. Our results support previous studies which suggest that non-human primate populations, when compared to human studies, have increased power to detect genetic associations with intermediate regulatory phenotypes (Anderson, Vilgalys, and Tung 2020; Fan et al. 2019; Jasinska et al. 2017; Tung et al. 2015). This study also further validates the potential for joint genotyping and functional analyses in non-human species, including from RRBS data alone (Fan et al. 2019; Tung et al. 2015). This possibility allows researchers to investigate genetic associations with molecular phenotypes, as well as incorporate important relatedness information for individuals in their assessment of environmental effects when more costly whole genome sequencing is not feasible.

### 4.1 ∣ Sources of Methylation Variation

Consistent with current literature on the factors driving methylation variation, we found that local genetic context explains more methylation variation on average than demographic effects, namely sex and age (Lea et al. 2017). This observation agrees with results from large human cohort studies (Bergstedt et al. 2022; Czamara et al. 2021). The majority of age-associated sites decreased with age, in support of the trend of global hypomethylation during ageing (Unnikrishnan et al. 2018), and the majority of sex-associated sites had higher methylation in females, supporting human whole blood results showing more female-biased autosomal CpGs (Bergstedt et al. 2022; Grant et al. 2022). The significant overlap of meQTL with age and sex effects is also observed in human cohorts (Gopalan et al. 2017; Mulder et al. 2021; Traglia, Bout, and Weiss 2022). Genetic effects on gene regulation are often context-dependent and hypothesized to explain a portion of the missing heritability in complex phenotypes (Czamara et al. 2019; Findley et al. 2021; Runcie et al. 2013). Future work is needed to determine the environmental contribution to DNA methylation variation in the Cayo macaque population, how this compares with genetic and demographic effects and how they interact with widespread genetic effects to generate cellular immune variation.

### 4.2 ∣ Properties of meQTL

meQTL are more likely to be found in active regions of the genome, like open chromatin, active TSSs and CpG islands. This is similar to human whole blood meQTL findings (Gutierrez-Arcelus et al. 2013; Hawe et al. 2022; Huan et al. 2019), and supports the hypothesis that genotype-dependent methylation is likely to occur in regions where sequence context may disrupt protein binding and impact downstream regulatory activity. However, a study in wolves and baboons showed a depletion of meQTL in CpG islands, and an enrichment in open seas (Fan et al. 2019). We further annotated the baboon meQTL using the approach we took here, and found similar enrichment near active TSSs, but a depletion in exons, and no enrichment for promoters (Suppoting Information Section 6.1, Figure S40-S44, Table S1.15). We may have greater power to detect genetic effects on methylation in additional regulatory regions. Furthermore, whilst promoter CpG islands are largely hypomethylated, many intergenic CpG islands and those within gene bodies are hypermethylated (Jeziorska et al. 2017; Jjingo et al. 2012) and these patterns can differ across species (Al Adhami et al. 2022).

meQTL may impact methylation levels through several mechanisms: for example, SNPs may occur within transcription factor binding sites, and altered binding may affect local epigenetic patterns, or SNPs may occur directly within CpG sites. Within species, the genotype of meQTL variants in TF binding sites is associated with altered binding affinity of the TF as well as nearby CpG methylation levels (Banovich et al. 2014). At a species level, human-specific differentially methylated regions frequently overlap TF binding sites with human-specific mutations (Hernando-Herraez et al. 2015). We tested two predictions from each of the proposed mechanisms: (1) that meQTLs would be enriched near vertebrate TF binding sites and (2) that some meQTLs would be a CpG dinucleotide. In support of the first prediction, we found a large number of enriched TF-binding site motifs in meQTL regions, consistent with the hypothesis that genotype-dependent TF-binding links QTLs to nearby methylation variation. In support of the second prediction, meQTL SNPs were more likely than non-meQTL to fall in associated CpGs. This observation also serves as a validation, confirming the model is highlighting expected associations between genotype and methylation levels.

Interestingly, the majority of the top enriched motifs belonged to a single family of TFs, the erythroblast transformation-specific (ETS) family, whose binding is known to be methylation-sensitive (Stephens and Poon 2016). In previous work, ETS binding sites were highly enriched in methylation-dependent regulatory elements in a massively parallel reporter assay conducted in human blood-derived cell lines (Lea et al. 2018). This suggests that ETS binding may depend on both genotype and methylation, with genotype-dependent binding also altering local methylation patterns. The second most enriched family were Zinc Fingers, including the chromatin regulator *CTCF*, implicated in genotype-methylation interactions in *cis* (Banovich et al. 2014; Schulz et al. 2017; Shi et al. 2014) and *trans* (Hawe et al. 2022). Many top TFs play roles in immune regulation (Luo et al. 2017; Seifert et al. 2019; You et al. 2013) are preferentially expressed in blood cells (David et al. 2022; Birdsey et al. 2008), and ETS TFs play an important role in haematopoiesis (Hogart et al. 2012). Genetic effects on methylation in whole blood may be exerted through disruption of blood-specific TF activity.

### 4.3 ∣ Phenotypic Impact of meQTL

The highest fold enrichment for meQTL-associated genes was in pathways related to T cell differentiation during the adaptive immune response, viral defence and pattern recognition receptor signalling, and meQTL were overrepresented near genes that are differentially expressed during immune stimulations. To further confirm the functional impact of macaque meQTL on immune cell gene regulation, we leveraged an available whole blood gene expression dataset to test for genotype-expression and methylation-expression relationships. We have a smaller sample and less statistical power in the eQTL analysis compared to the meQTL, and we did not have paired samples. Additionally, emerging work suggests regulatory variants may be more likely to result in a detectable stable change in methylation than expression. For example, a recent multitissue meQTL study found that many meQTL colocalized with GWAS traits did not have a corresponding bulk tissue eQTL, such that the associated eQTL may only exist in a different context (e.g., a different cell, developmental or disease state) (Oliva et al. 2023). Nevertheless, eQTL mapping revealed 332 SNPs impacting both nearby methylation and expression levels. Compared to non-eQTL meQTL tested, *cis*-eQTL were further enriched in open chromatin, CpG islands and active TSSs, and depleted in silent states. They were also less likely to be in enhancers, which are distal regulatory elements. This aligns with current primate data, where *cis*-eQTL are enriched near TSSs (Fair et al. 2020; Jasinska et al. 2017; Tung et al. 2015).

meQTL–eQTL eGenes are as predicted enriched for immune functions, particularly cellular interactions and signalling. Immune genes often evolve rapidly (Judd et al. 2021) and may be more likely to harbour functional genetic variation in their coding and regulatory regions (Jin et al. 2018; Simons et al. 2017). This is consistent with the enrichment of antigen processing for eGenes shared between baboons and humans, as well as humans and chimpanzees, and their low conservation scores compared to non-eGenes (Fair et al. 2020; Tung et al. 2015). meQTL- and eQTL-associated genes or CpGs may often be lineage-specific and more likely to be differentially expressed or methylated between species or populations, as is the case for meQTL-associated CpGs in humans and baboons (Husquin et al. 2018; Vilgalys 2019). In support of this, we found that meQTL CpGs were enriched in differentially methylated regions between macaque species. For a subset of meQTL–eQTL, CpG methylation and eGene expression levels were correlated, supporting the functional relationship between DNA methylation and expression, and the possibility that the SNPs effects on expression is mediated by DNA methylation (or vice versa) (Gutierrez-Arcelus et al. 2013; Husquin et al. 2018).

### 4.4 ∣ Limitations and Future Directions

This study had several limitations. First, we are only looking at proximal *cis* genetic effects rather than *trans* effects which are more difficult to detect but are significant contributors to interindividual methylation variation (Hawe et al. 2022; Husquin et al. 2018). The enrichment of meQTL near known regulatory enzymes, including DNA methyltransferases, suggests some *cis*-meQTL may indeed also be acting in *trans*. Second, in our IMAGE analyses, we were limited to testing SNP–CpG combinations from the same sequencing read. This was not necessarily a disadvantage, as proximal *cis* variants are both more common (Hawe et al. 2022; Husquin et al. 2018; McClay et al. 2015) and have larger effects on methylation than more distal variants, with the effect size being inversely proportional to the distance between the variant and CpG (Villicaña and Bell 2021). Finally, whilst we used the best performing genotype calling method for RRBS reads available (Lindner et al. 2022), at an individual level genotype calls from bisulfite sequencing data are likely more error prone than those from whole genome data given the uncertainty at C/T variants.

Future work expanding the range of SNPs with whole genome sequence data will allow for colocalization and mediation analyses, and shed light on the underlying relationships between genotype, methylation and expression in the macaque. Differential methylation can serve as a biomarker of health, disease progression and biological ageing (Belsky et al. 2022; Cappozzo et al. 2022; Huan et al. 2022; Kundakovic et al. 2015; Salameh, Bejaoui, and El Hajj 2020). Additional research is needed to determine the potential for the regulatory loci identified here to mediate interindividual differences in key phenotypic traits, such as the stress or immune response. The impact of meQTL on regulatory activity may also be confirmed in vitro in macaque cell lines, using techniques like mSTARR-seq (Lea et al. 2018). Finally, comparative work may highlight important patterns of genetic effects on methylation, elucidating the mechanism and process—how gene regulation and DNA methylation evolve within and between species, and how variation is generated and maintained in populations.

## Supplementary Material

Supplementary Tables 1

Supplementary Tables 2

Supplementary Information

Additional supporting information can be found online in the Supporting Information section.

## Data Availability

Code written for analyses and RRBS metadata is publicly available on GitHub at https://github.com/Cec701/genetic_architecture_DNAm_rhesus. Complete model outputs are also available on Zenodo (10.5281/zenodo.10800972). Raw RRBS reads are deposited on the National Center for Biotechnology Information (NCBI) sequence read archive (SRA) at accession PRJNA610241 and raw WGS reads at NCBI SRA accession PRJNA1126685. Raw RNA sequencing reads are available on the NCBI SRA (PRJNA715739; Watowich et al. 2021).
