# Supplementary material for: Genetic Architecture of Immune Cell DNA Methylation in the Rhesus Macaque
Source: Mol Ecol. Author manuscript; Available in PMC 2025 Jul 17. (PMC12269644; doi:10.1111/mec.17576)
Supplement: Supplementary Information [file NIHMS2092291-supplement-Supplementary_Information.pdf]

MOLECULAR ECOLOGY

Supplemental Information for:

Genetic architecture of immune cell DNA methylation in the rhesus macaque

Christina E. Costa; Marina M. Watowich; Elisabeth A. Goldman; Kirstin Sterner; Josue E. Negron-Del Valle; Daniel Phillips; Cayo Biobank Research Unit; Michael Platt; Michael J. Montague; Lauren J. N. Brent; James P. Higham; Noah Snyder-Mackler; Amanda J. Lea.

Table of Contents:

**S1. Dataset Overlap**	Page 2
**S2. meQTL Analysis**	Page 3
**S3. Sigma^2 Filtering**	Page 27
**S4. eQTL Analysis**	Page 35
**S5. CpG-Gene Correlation Analysis**	Page 47
**S6. Baboon and Wolf meQTL Enrichment**	Page 52
**Supplemental References**	Page 58

MOLECULAR ECOLOGY

S1. Dataset Overlap

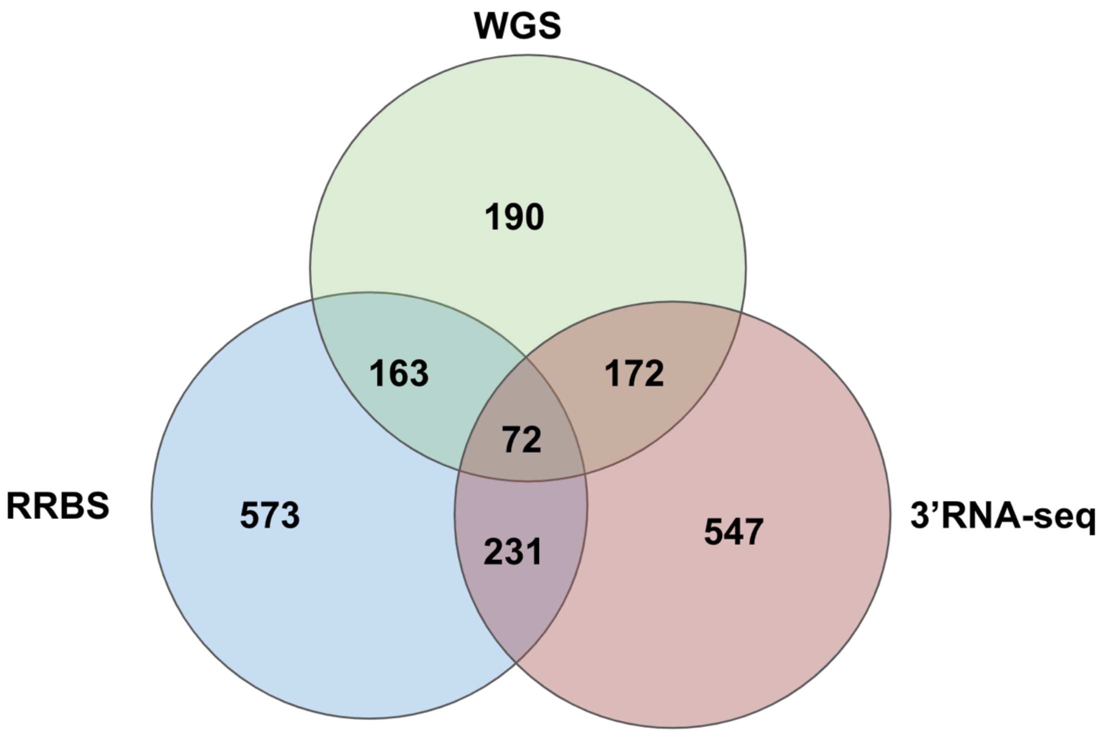

Dataset	Number of Samples	Number of Individuals	Males	Females	Age Range
**RRBS**	573	500	223	277	0.11-28.78
**RNAseq**	547	445	240	204	0.11-27.93
**WGS**	190	190	78	112	3.18-27.93
**RRBS-WGS**	163	163	69	94	3.18-27.93
**RNAseq-WGS**	172	120	52	68	6.18-27.93
**RRBS-RNAseq**	231	226	103	123	0.11-27.93

Supplemental Figure 1. Overlap of sequencing datasets. (1). Venn diagram: numbers represent samples with that data type. **(2).** Demographic table for each independent and overlapping modality, including number of unique individuals.

MOLECULAR ECOLOGY

S2. meQTL Analysis
Supplemental Methods

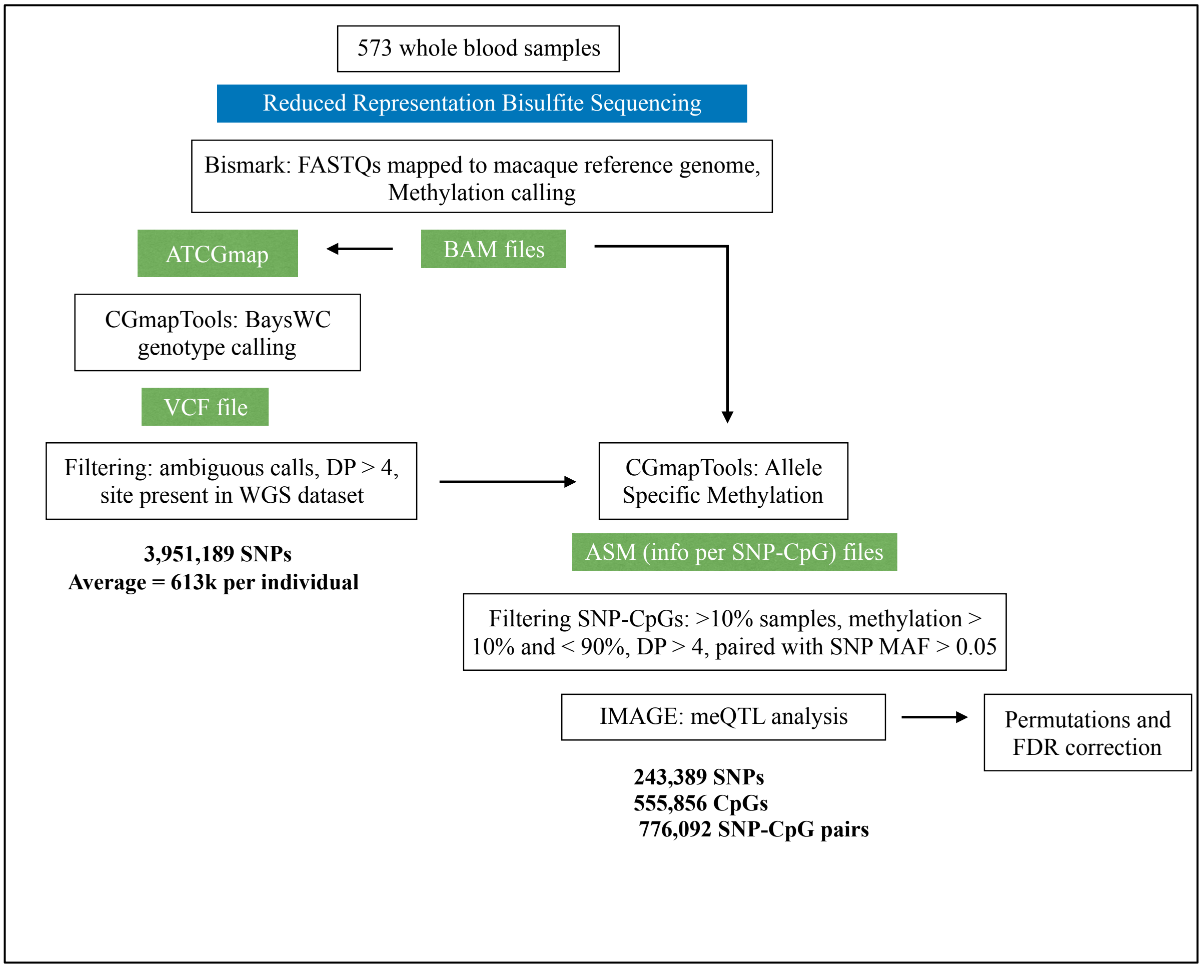

2.1 SNP-CpG Filtering

Filter 1 (Methylation measured < 10% samples) → 3,763,384 SNP-CpG pairs

Filter 2 (<10 or >90 percent methylation in > 90% of samples) → 2,960,724 SNP-CpG pairs

Filter 3 (Mean read depth/DP < 5) → 2,782,353 SNP-CpG pairs

Filter 4 (SNP MAF < 0.05) → 794,886 SNP-CpG pairs

MOLECULAR ECOLOGY

2.2 Comparison with whole genome genotype calls

To assess the accuracy of CGmapTools single nucleotide variant calling results, GATK derived genotype calls from the whole genome data were available for a subset of RRBS samples (n = 163). BCFtools 'gtcheck' (Danecek et al., 2021) was used to compare genotype calls for all sites in the IMAGE RRBS VCF (filtered for DP > 4, no vague calls, also in WGS dataset) and the full WGS dataset for SNPs passing GATK germline variant quality control filters (QualByDepth > 2.0, mapping quality (MQ) > 35.0, FS (strand bias) < 60, MQRankSum > -12.5, and ReadPosRankSum > -8.0). 582,303 sites were compared between samples in total (mean per sample 32,798.18, median= 30,513, SD= 14,532.92). Calculating the number of mismatched genotypes directly (-e 0 flag), the average number of mismatches between the same (matched) samples was 3,970.56 (median= 3,386, SD= 2,596.54). The percentage of mismatched sites was lower in matched samples (mean = 10.29 %, median = 9.78%, SD=2.63%) than in non-matched samples (mean = 11.49%, median = 11.11%, SD= 3.92%). Results suggest >80% agreement on average between genotype calls between the two datasets for sites compared here (**Fig S6A, B**) Limiting the comparisons to homozygous sites only, shows greater than 90% agreement between the two datasets on average, with the mean percentage of mismatched genotypes at 7.92% (median = 7.78%, SD=3.43%) (**Fig S6C**).

2.3 Models and percent variance explained (PVE) equations

Our analyses rely on the mixed effects modeling framework established in quantitative and population genetics for designs that include families or relatives, genetic structure, and/or repeated measures (e.g., as laid out in Kang *et al*., 2008 and Zhou & Stephens, 2012). IMAGE and PQLseq expand on this framework to model the count-based nature of the DNA methylation data, at a given CpG site, by including a logit link function as follows:

$$y_i \sim Bin(r_i, \pi_i)$$

$$logit(\pi_i) = \mu + x_i\beta + u_i + e_i$$

$$u_i \sim MVN(0, \sigma^2 h^2 K)$$

$$e_i \sim MVN(0, \sigma^2 (1 - h^2) I)$$

MOLECULAR ECOLOGY

where y_i and r_i are the methylated read counts and total read counts for individual i that follow a binomial distribution (note that IMAGE also includes modifications to break these counts down by allele, which we ignore here for the purpose of explaining the general mixed effects framework). π_i is the true methylation level, μ is the intercept, x_i is the predictor or interest, in this case genotype, and β is the effect size of the predictor. u_i is a random effect that controls for kinship and other sources of genetic structure. **K** is square matrix that models the covariance among samples. This covariance can be due to either familiar relatedness, population structure, and/or the inclusion of repeated samples; we note that several mixed effects modeling programs include convenience functions (in the form of an incidence matrix) to map repeated sample identities onto pairwise genetic relationships between individuals (e.g., EMMREML (Akdemir & Okeke, 2015), EMMA (Kang et al., 2008)). Neither IMAGE nor PQLseq includes these convenience functions, and thus we manually expanded our K matrix such that repeated measures from the same individual get a value of 1 in the K matrix (e.g., as in Cuomo et al., 2021). **I** is the identity matrix, $\sigma^2 h^2$ is the genetic variance component, and $\sigma^2(1-h^2)$ is the environmental variance component. *MVN* denotes the multivariate normal distribution.

a. IMAGE meQTL (Binomial Mixed Model)

Methylated/total read counts at CpG site ~ genotype at associate SNP + sex + age + batch + (1|relatedness matrix (pedigree))

b. PQLseq meQTL (Binomial Mixed Model)

Methylated/total read counts at CpG site ~ genotype at associate SNP + sex + age + batch + (1|relatedness matrix (pedigree))

c. PVE (Linear Model)

Methylated/total read counts at CpG site ~ sequencing batch + sex + age

First, linear models with all covariates were run for every CpG using the 'lm' function in R, with the number of methylated to total reads as the dependent variable, and sequencing batch, sex, and age as fixed effects. The results were extracted using the 'summary' function, and the residuals were taken for each model. The variance of the residuals for each model (each CpG site) was obtained using the 'var' function (var(x_res)).

MOLECULAR ECOLOGY

d. PVE equations

IMAGE PVE: The PVE was then calculated as follows for each SNP-CpG pair, taking var(x_res) for the corresponding CpG site. Beta (β) represents the genotype effect size for each SNP-CpG pair, and sigma squared ($sigma^2$) represents the total variance component.

$$PVE = ((\beta^2 * variance(x\_res)) / (\beta^2 * variance(x\_res) + sigma^2)$$

PQLseq PVE: Percent methylation variance explained (PVE) by each predictor was calculated using the following equations. Variance in each predictor was calculated separately for each CpG site depending on the individuals included in that model.

$$A\_covariate = (\beta\_covariate)^2 * (variance(covariate))$$

$$Total\_variance = \sum (A\_covariate\_i \ldots A\_covariate\_n) + sigma^2$$

$$PVE\_covariate = A\_covariate/Total\_variance$$

2.4 meQTL modeling without repeated samples (unique individuals)

Removing repeated DNA methylation measures from the same individuals in the meQTL analysis did not significantly alter the results. The original models account for these replicates with a random effect generated from the relatedness matrix, where samples from the same individual years apart are related at r=1.0 (i.e., identical). We therefore present the original IMAGE and PQLseq results (n=573 samples) in the paper.

In the PQLseq model with unique individuals (n=500), 80.07% of SNP-CpGs remain significant (5% FDR). The model identified 229,380 significant (5% FDR) SNP-CpG pairs in total, 94.39% (n= 216,504) of which were also significant in the complete model. The betas for the PQLseq analysis with and without repeated samples (n=500 unique individuals) are highly correlated for SNP-CpG pairs modeled in both (n= 572,732 SNP-CpGs, p-value < 2.2e-16, rho= 0.869). This correlation increases when limiting to sites that were significant meQTL in the original analysis (5% FDR, n= 270,395 SNP-CpGs, rho = 0.886, p-value < 2.2e-16) and increases further when considering sites significant (5% FDR) in both models (n=216,504 SNP-CpGs, rho = 0.97, p-value < 2.2e-16) (**Fig S14**).

2.5 IMAGE permutations and multiple testing correction

We used a permutation or empirical null based multiple testing correction method, as we did not have prior assumptions about the null distribution of p-values from the IMAGE analysis. We used the built-in permutation function in IMAGE (Fan *et al.*, 2019). For each chromosome, total and methylated read counts for each allele were combined and permuted. Total and methylated read counts for the first allele were taken from all samples both homozygous and heterozygous. These were then combined with the total and methylated read counts for the second allele from all heterozygotes and shuffled. These permuted values are reinserted into the appropriate locations in the original data frame keeping homozygotes, heterozygotes, and missing data the same. This permuted data was used as input to IMAGE and repeated 10 times per chromosome. The permuted p-values, along with the actual p-values, were input into the empirical null based multiple testing correction R program 'perm.fdr', which uses the Storey-Tibshirani method (Storey & Tibshirani, 2003) to provide corresponding q-values for the list of p-values. We used a false discovery rate (FDR) cutoff of 0.05 (5%). (**Fig S10**).

2.6 Model comparisons (PQLseq versus IMAGE)

To further validate the meQTL, we performed meQTL mapping within PQLseq (Sun et al., 2019). Modeling the same SNP-CpG pairs, this revealed 298,021 whole blood *cis* meQTL (5% FDR) out of 630,826 SNP-CpG pairs (47.24%). 90.38% of *cis* meQTL in PQLseq are also a significant meQTL in IMAGE, representing 52.18% of the significant IMAGE meQTL (**Fig S13**). With a less conservative FDR (20%) this increases to 64.53% of IMAGE meQTL. PQLseq models the minor allele as the effect allele, while IMAGE models the non-reference allele. For sites where the non-reference is the minor allele, betas are strongly positively correlated for significant meQTL in both models (rho=0.795, p-value < 2.2e-16, **Fig S13**). Where the non-reference is major, they are negatively correlated (rho = -0.165, p-value < 2.2e-16).

PVE estimates between the two methods for significant IMAGE meQTL (5% FDR) modeled in both (n=420,812, with $sigma^2 > 0$ filter = 245,992), were moderately positively correlated (p-value < 2.2e-16; Spearman's rho: 0.411). This correlation estimate increases when limiting to sites that were significant meQTL (5% FDR) in IMAGE and in PQLseq (n=269,346,

with $sigma^2 > 0$ filter = 158,398, p-value < 2.2e-16; Spearman's rho: 0.467). $Sigma^2$ was also moderately positively correlated between the models (p-value < 2.2e-16; Spearman's rho: 0.517).

From the PQLseq output, 162,847 CpGs had significant batch effects (5% FDR) and a large amount of variance was explained by sequencing batch (mean = 13.48%), which was controlled for in all models (**Fig S15**).

2.7 meQTL Gene Set Enrichment Analysis (GSEA)

To further support the meQTL Gene Ontology enrichment results, we used the Broad Institute GSEA (gene set enrichment analysis) (Subramanian et al., 2005). We sorted the genes by the proportion of significant meQTL CpGs to tested CpGs near the gene and used this ranked list as input (STable 1.8). We tested for enrichment of five gene sets in the Molecular Signatures Database (MSigDB v2023.1.Hs): Hallmark (Liberzon et al., 2015), GO biological process (BP), GO molecular function (MF), C7 immunologic signature (ImmuneSigDB; Godec et al., 2016), and C8 cell type signatures (FDR 20%). The parameters for the Hallmark pathway were minimum genes =10, maximum = 500, and for all the rest, minimum genes = 10, maximum = 2000. Cell type signature genes include marker genes from human single-cell sequencing studies. Results are shown in Supplemental Tables File 1 (STables1.9-12).

2.8 LiftOver chromatin state annotations from human to macaque coordinates

Using the default settings, chromatin state activity annotations (PBMCs) were converted from human (hg38) to rhesus macaque (mmul_10) genome coordinates with UCSC Genome Browser program LiftOver. 332,398 records (96.69%) were successfully converted (11,389 failed).

MOLECULAR ECOLOGY

S2. Supplemental Figures

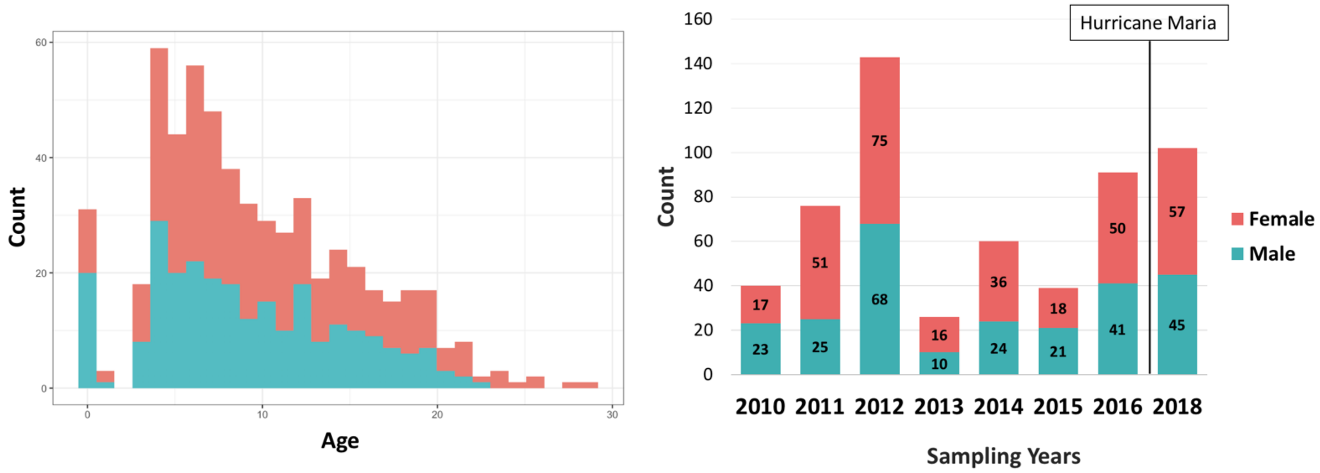

Supplemental Figure 2. Sample information for rhesus macaques in RRBS dataset. (1). Histogram of sample count by age, colored by sex. **(2).** Number of samples collected by year, colored by sex. No sampling occurred in 2017 due to Hurricane Maria.

MOLECULAR ECOLOGY

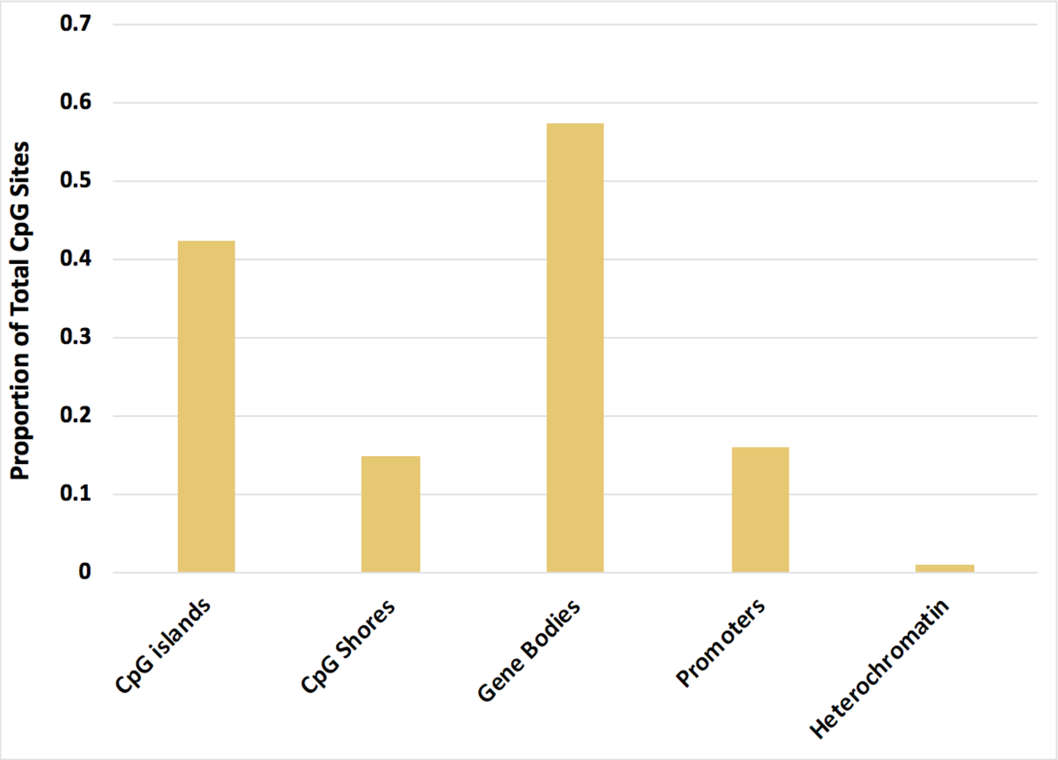

Supplemental Figure 3. Proportion of RRBS CpGs in certain genomic regions. Proportions match expectations for RRBS dataset, as shown in Lea et al., 2017.

MOLECULAR ECOLOGY

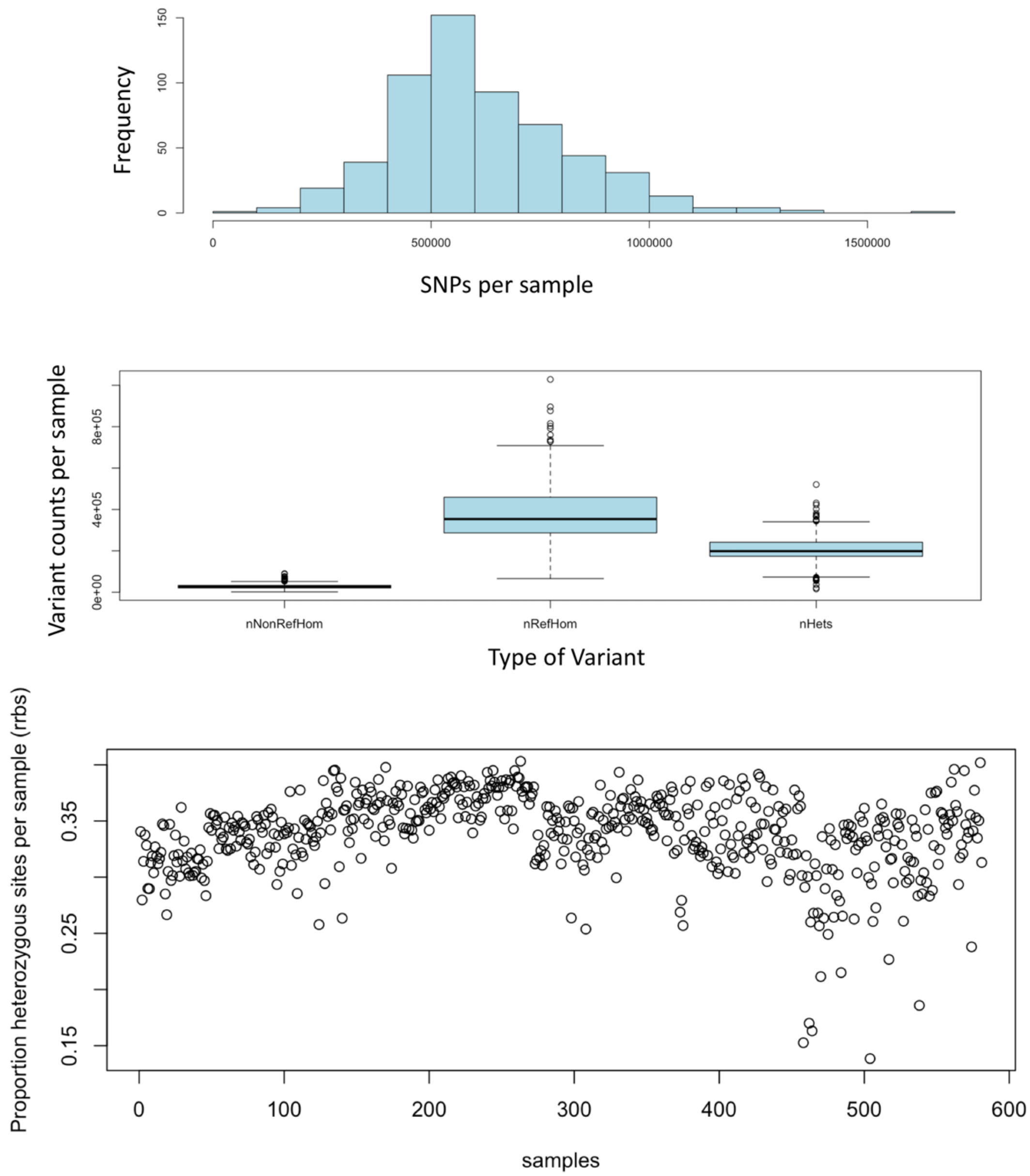

Supplemental Figure 4. Description of RRBS genotyping. (1). Histogram of SNPs called per sample. **(2)**. Counts of variant types called in the dataset across samples. nRefHom = Reference homozygous, nNonRefHom = Non-reference homozygous, nHets = Heterozygous. **(3).** Plot of heterozygosity per RRBS sample.

MOLECULAR ECOLOGY

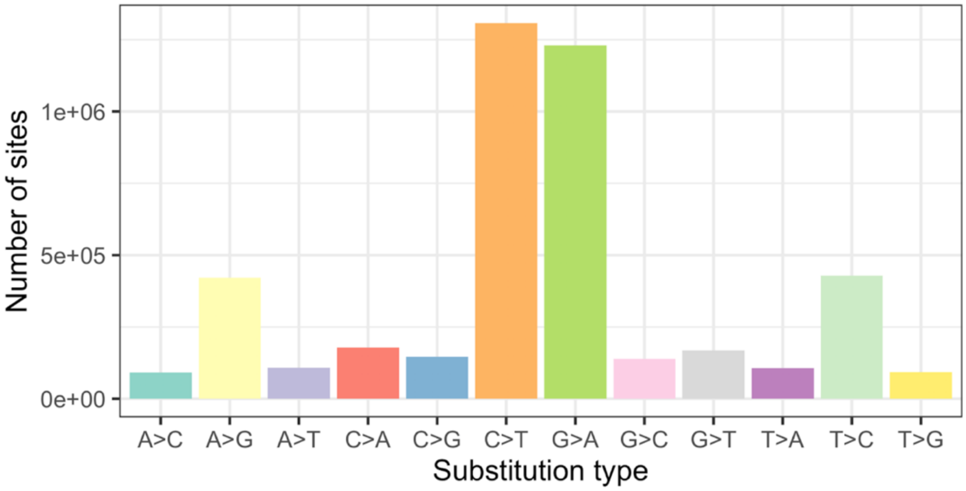

Transitions (TS)	Transversions (TV)	TS/TV
3,387,509	1,030,132	3.29

Supplemental Figure 5. RRBS substitution statistics. (1). Counts of substitution type. **(2)**. Table with TS and TV counts and ratio.

MOLECULAR ECOLOGY

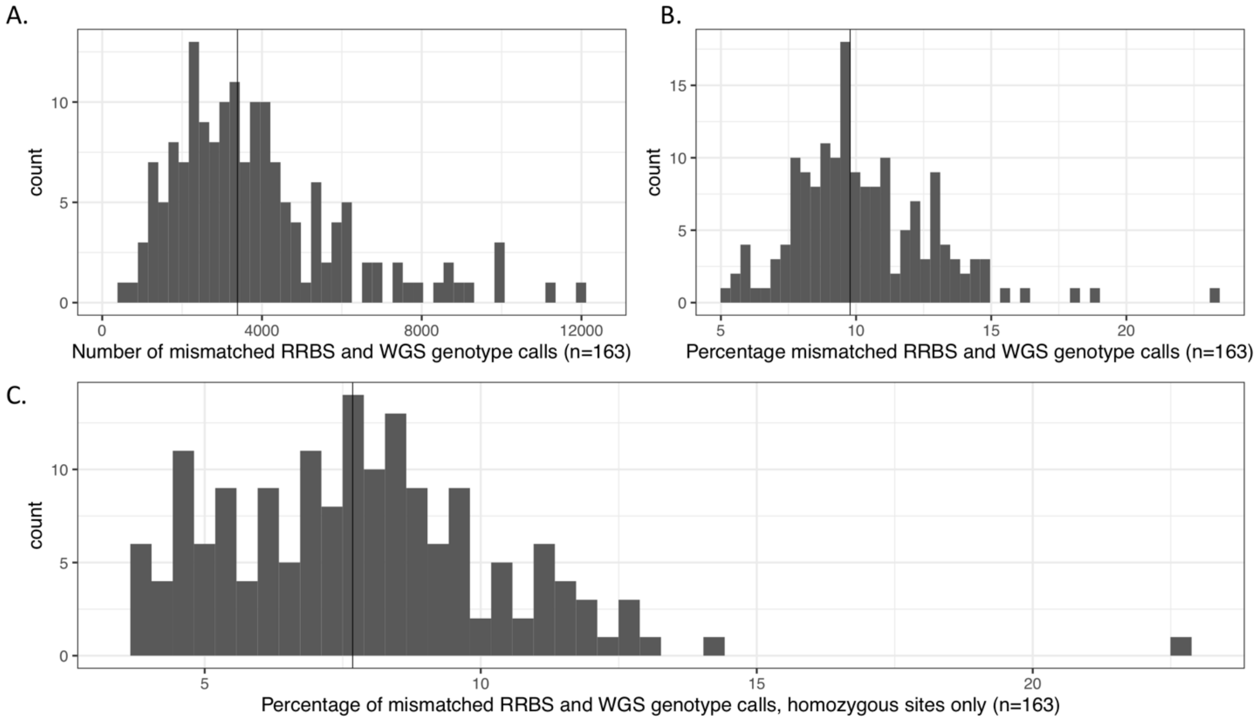

Supplemental Figure 6. Mismatched genotypes in the same samples, for genotypes called from RRBS versus WGS data. Average number of sites compared: 32,798.18. **(A).** Number of genotypes that do not agree in the RRBS and WGS data **(B).** Percentage of genotypes compared that do not agree in the RRBS and WGS data. **(C).** Percentage of genotypes compared that do not agree in the RRBS and WGS data at homozygous sites only. Black lines indicate median.

MOLECULAR ECOLOGY

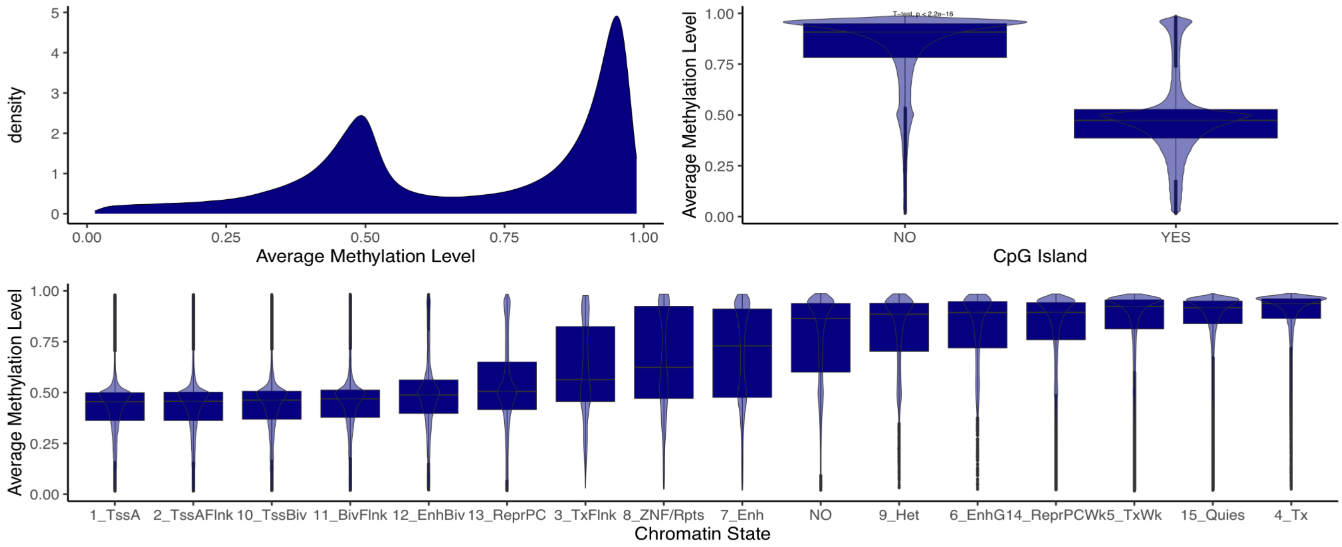

Supplemental Figure 7. Methylation level of CpG sites included in the IMAGE analysis (post-filtering). (1) density plot, Methylation level = proportion of methylated/total reads. **(2).** Average methylation level of macaque CpGs in CpG islands and **(3).** across chromatin states.

MOLECULAR ECOLOGY

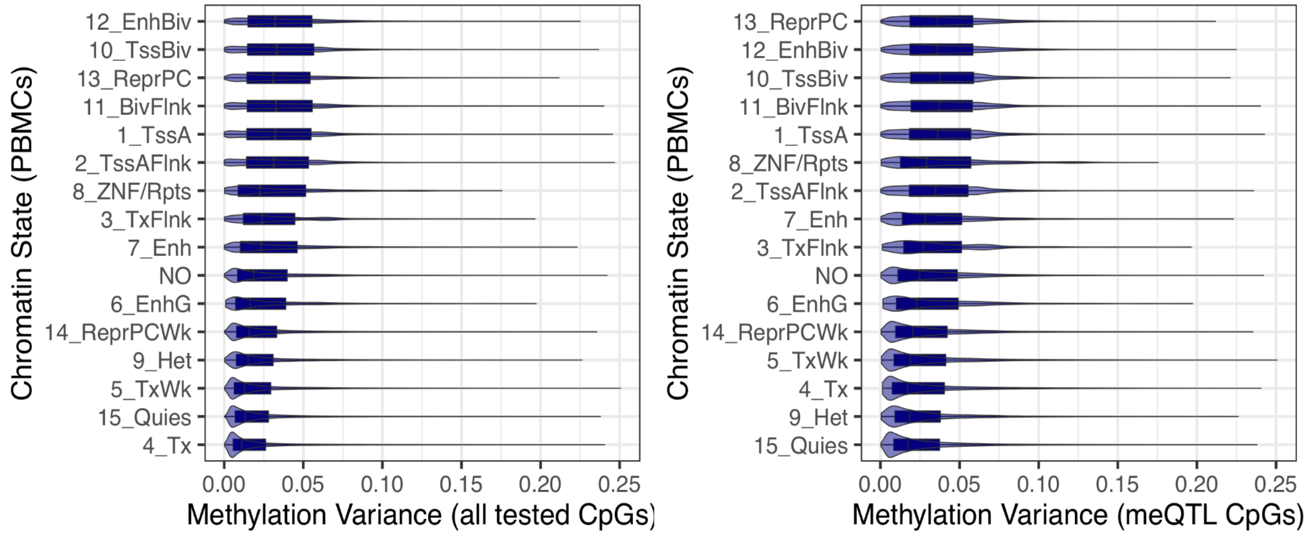

Supplemental Figure 8. CpG Methylation Variance (1). Variance in methylation levels across chromatin states for all CpGs in analysis, **(2)** and significant meQTL CpGs**.**

MOLECULAR ECOLOGY

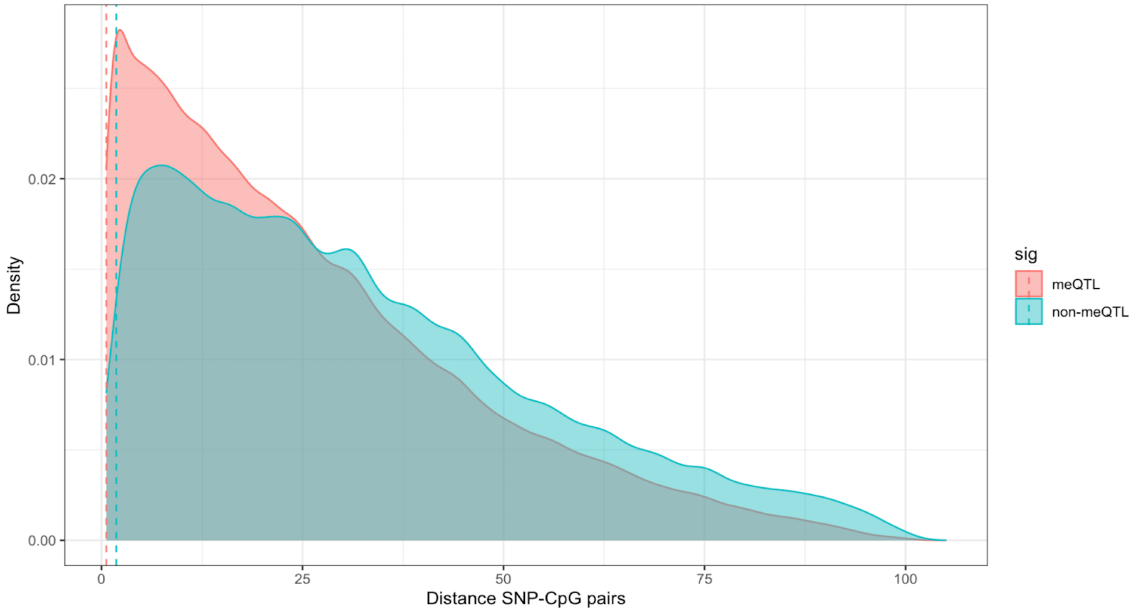

Supplemental Figure 9. Distance (bp) between significant and non-significant SNP-CpG pairs. Average distance between meQTL SNP-CpGs = 25.71 bp (median = 21, SD = 20.46).

MOLECULAR ECOLOGY

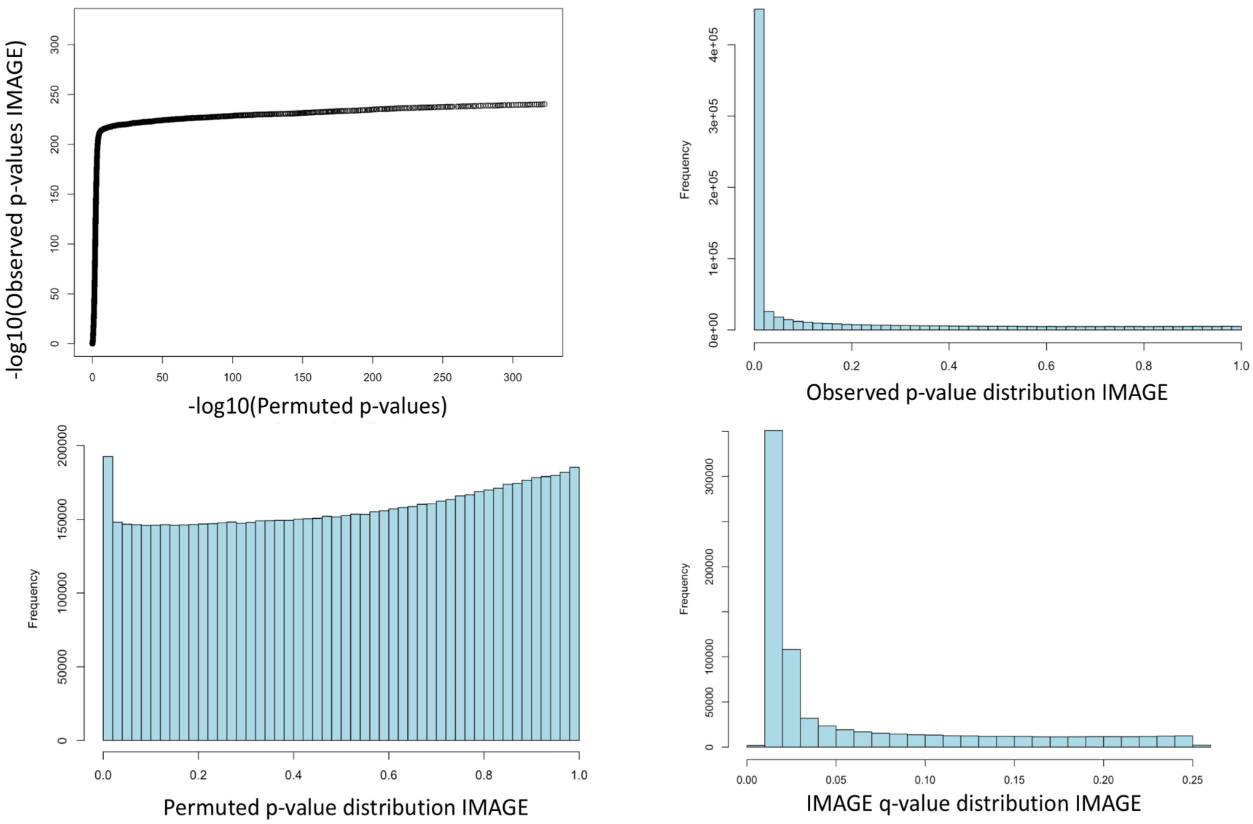

Supplemental Figure 10. IMAGE Modeling. (1). Q-Q plot of permuted and observed p-values in IMAGE analysis. **(2)**. Observed IMAGE p-values. **(3)**. Permuted p-values (10 permutations per chromosome, 200 total). **(4).** Empirical null based p-value correction. Histogram of q-values.

MOLECULAR ECOLOGY

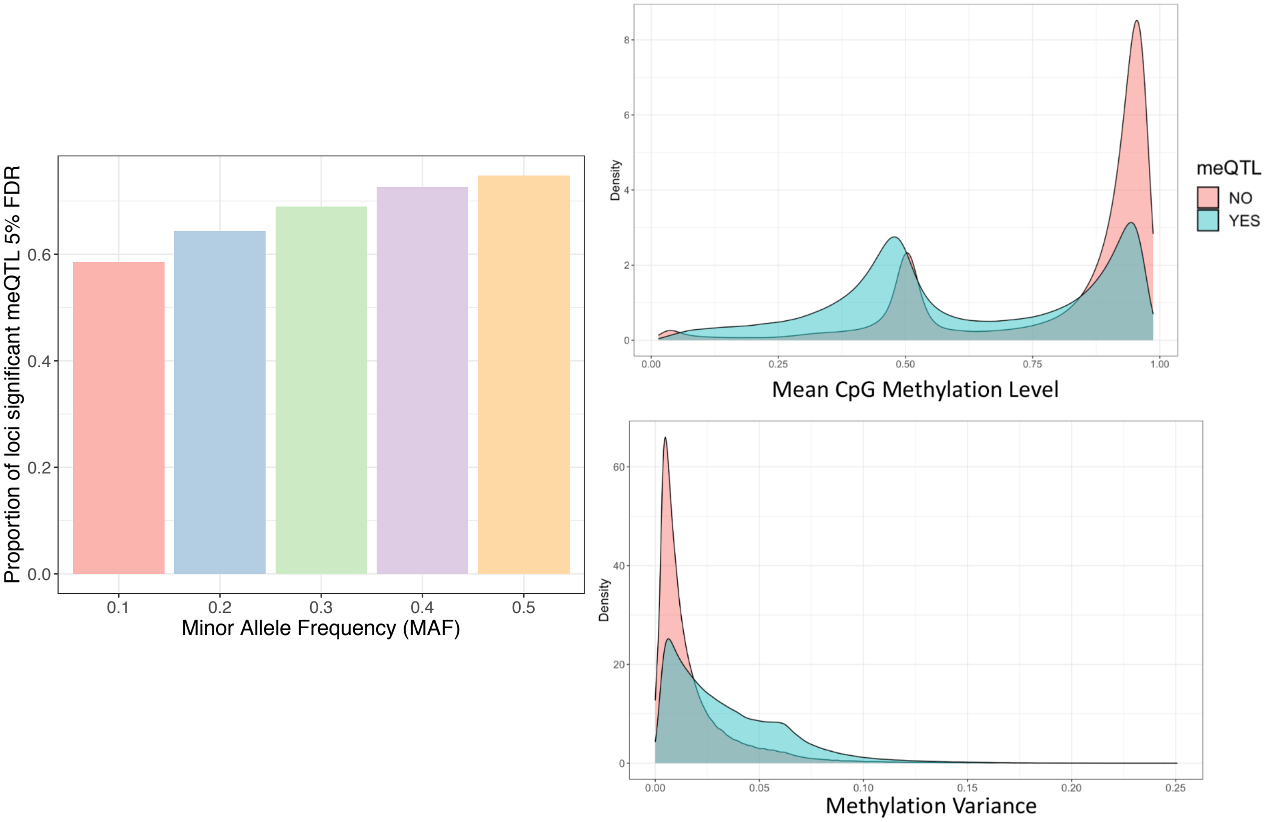

Supplemental Figure 11. ***cis*** **meQTL characteristics. (1)** Number of significant meQTL increases with minor allele frequency (MAF) of the SNP. **(2).** *(Above)* Average methylation level of significant meQTL and non-meQTL CpGs. *(Below)* Methylation variation across samples for meQTL and non-meQTL CpGs.

MOLECULAR ECOLOGY

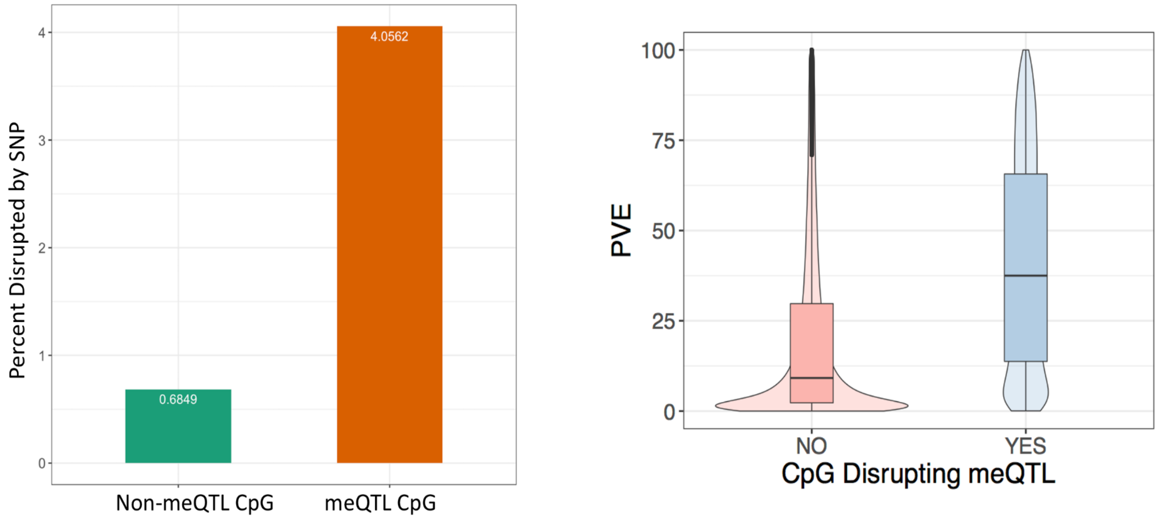

Supplemental Figure 12. CpG-disrupting SNPs. (1) Percent meQTL CpG disrupted by the associated SNP compared to non-meQTL CpGs. **(2)** CpG-disrupting SNPs may show distinct patterns; Consistent with this, average PVE by CpG-disrupting SNPs is greater than non-disruptive meQTL SNPs (t.test p-value < 2.2e-16).

MOLECULAR ECOLOGY

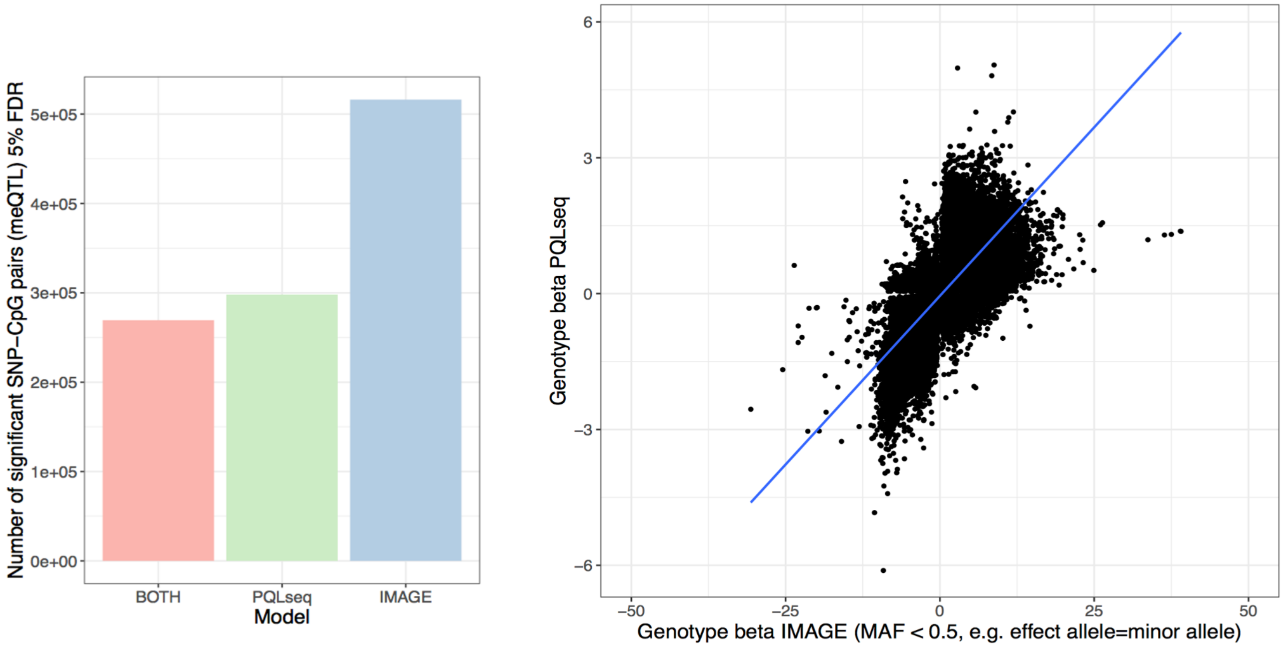

Supplemental Figure 13. IMAGE versus PQLseq meQTL mapping. (1). Number of meQTL (SNP-CpGs), 5% FDR, in each modeling framework and detected in both. **(2).** Genotype effect size from PQLseq plotted against genotype effect size from IMAGE, for SNP-CpGs pairs where the effect allele (non-reference allele) is also the minor allele (e.g., MAF < 0.5) in the IMAGE analysis. Line represents linear regression line ('lm' function).

MOLECULAR ECOLOGY

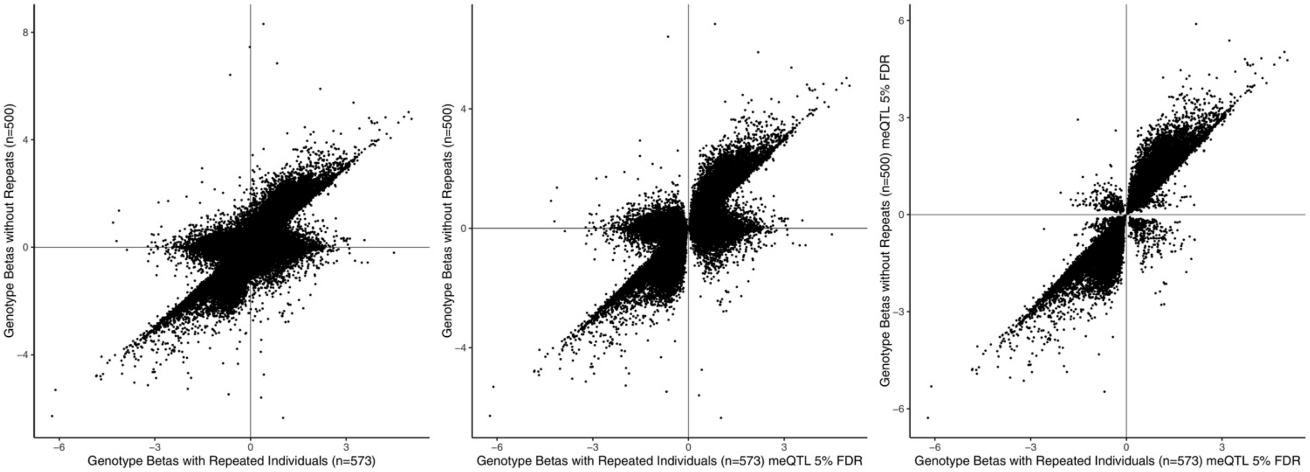

Supplemental Figure 14. Effect size correlation between SNP genotype-CpG methylation models in PQLseq with and without replicates. x-axis: PQLseq genotype effect size including repeated individuals (n=573), y-axis: PQLseq genotype effect size with only unique individuals (n=500). **(1)**. Betas for all modeled SNP-CpG pairs, **(2).** Betas for meQTL SNP-CpGs (5% FDR) in complete model, **(3)**. Betas for meQTL SNP-CpGs (5% FDR) in both models.

MOLECULAR ECOLOGY

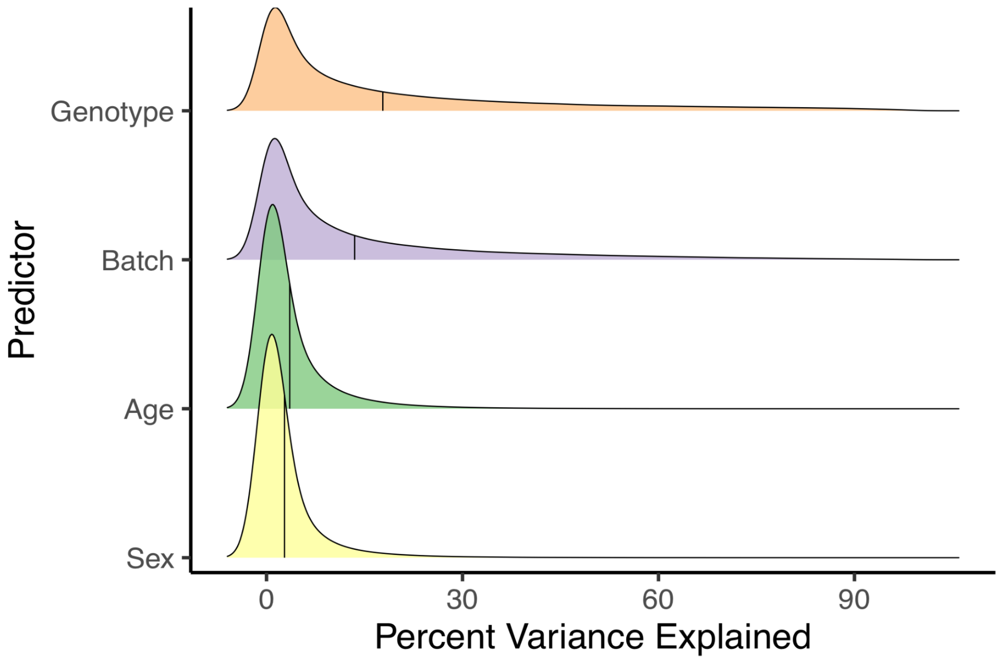

Supplemental Figure 15. Percent CpG methylation variance explained by RRBS sequencing batch effects. From the PQLseq output, 162,847 CpGs had significant batch effects (5% FDR) and a large amount of variance was explained by sequencing batch (mean = 13.48%), which was controlled for in all models.

MOLECULAR ECOLOGY

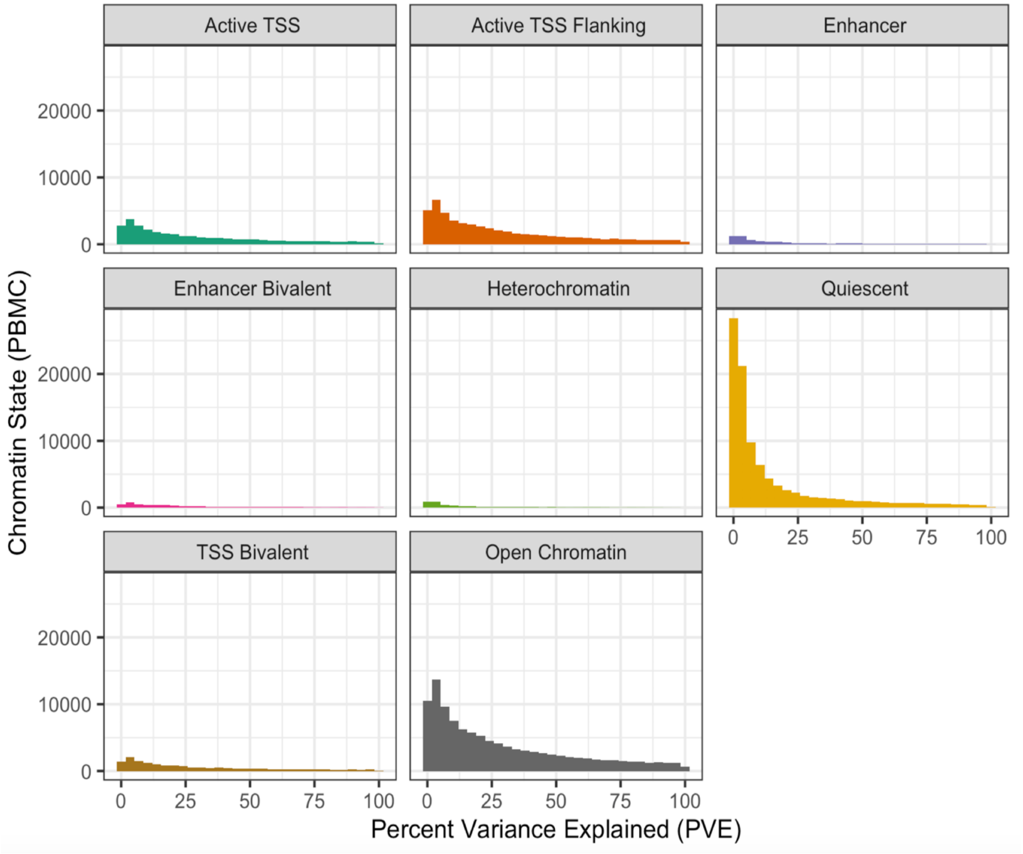

Supplemental Figure 16. Histograms of percent methylation variance explained (PVE) by genotype for SNP-CpG pairs in each chromatin state. IMAGE PVE estimates.

MOLECULAR ECOLOGY

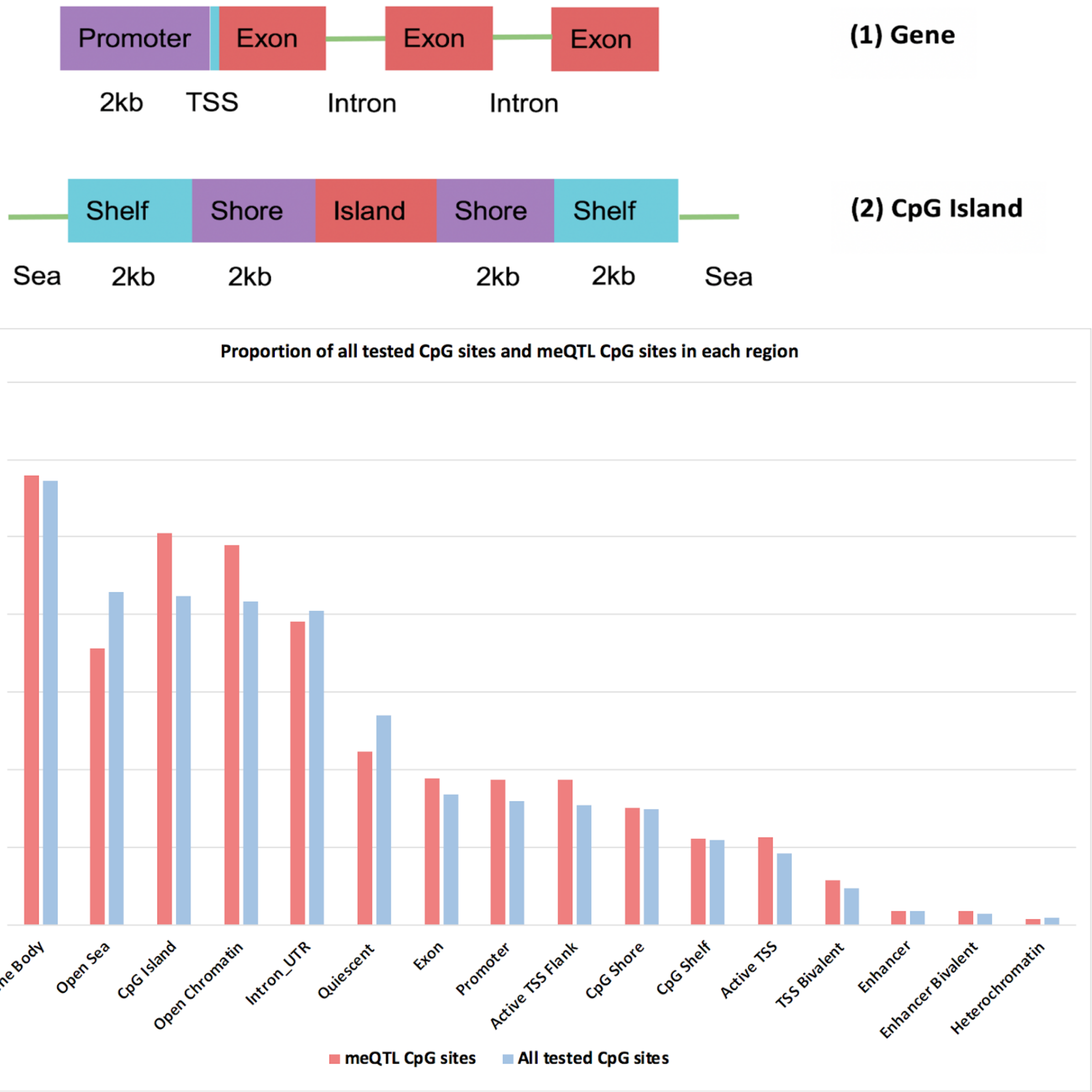

Supplemental Figure 17. meQTL CpG annotation and enrichment. (Above) Diagram shows (1) macaque gene features, and (2) CpG island annotations. **(Below)** Proportion of significant meQTL CpGs in different genomics regions and PBMC chromatin states compared to all tested CpGs.

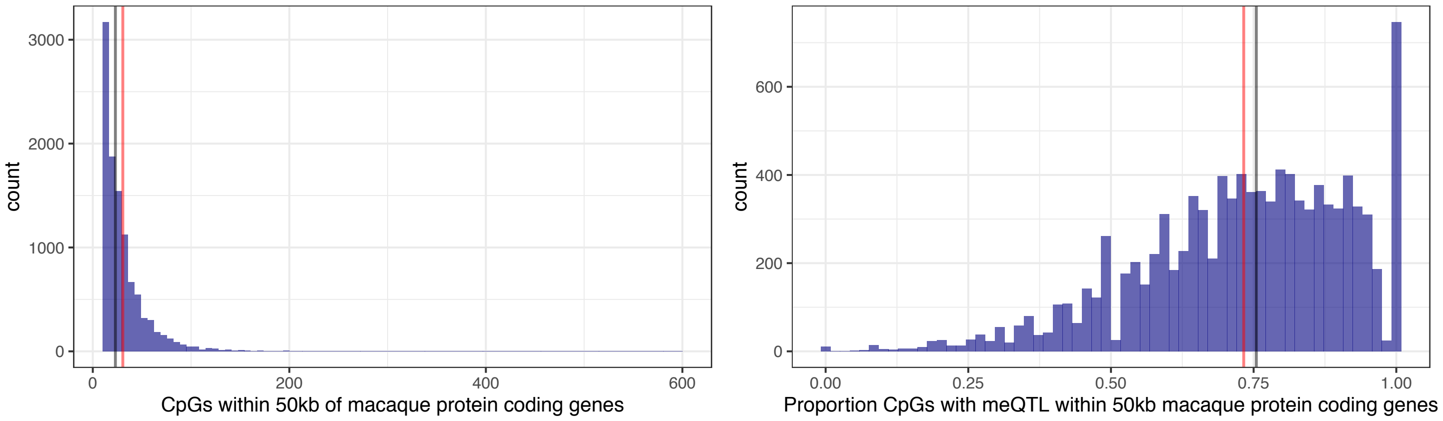

Supplemental Figure 18. Proportion of significant to tested CpGs near protein coding genes. n=10,415 genes. **(1)** Histogram of the number of CpGs within 50 kb of each macaque protein coding gene (for genes with at least 10 CpGs). Mean (red line) = 30.5, Median (black line) = 23, SD = 24.2, maximum = 594. **(2)** Histogram of the proportion of significant CpGs to total CpGs within 50 kb of each macaque protein coding gene (with at least 10 associated CpGs). Mean (red line) = 0.73, Median (black line) = 0.75, SD= 0.18.

MOLECULAR ECOLOGY

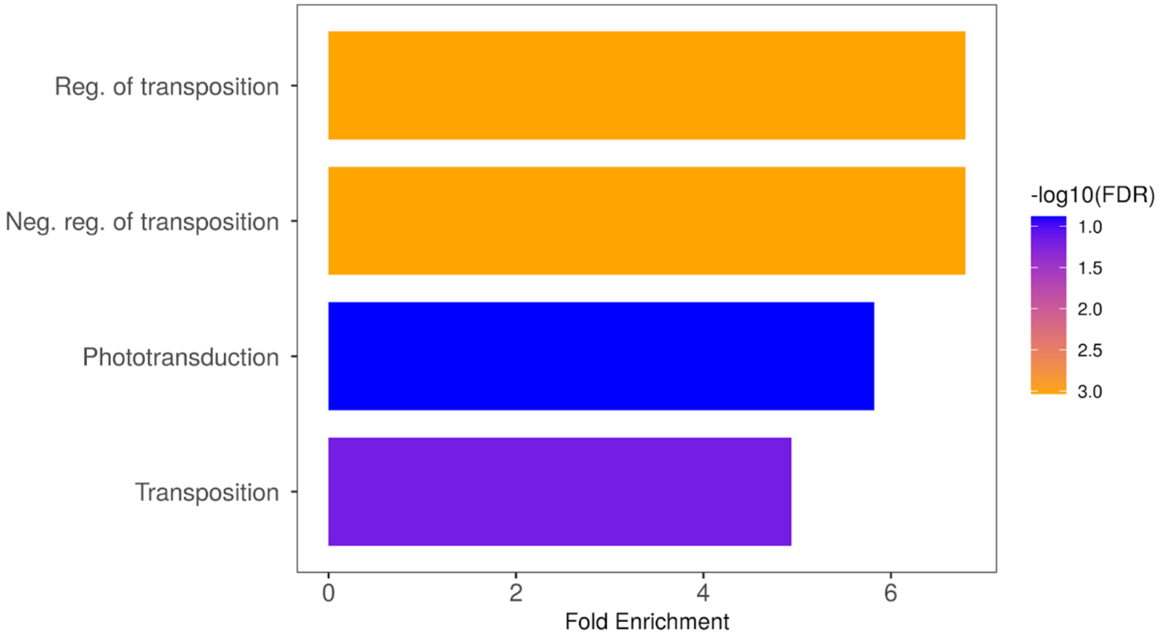

Supplemental Figure 19. Gene ontology enrichment for meQTL genes below the 5th percentile of permuted distribution. Genes harboring less significant meQTL than expected by chance (n=1,533).

MOLECULAR ECOLOGY

S3. Sigma^2 Filtering
Supplemental Methods

3.1 Sigma^2 Filtering

A component of the PQL modeling approach appears to be a computation of sigma^2 for some sites resulting in a sigma^2 of 0, even when the total variance in methylation levels is greater than 0, in particular for sites where sigma^2 is 0 but is still called a significant meQTL. This is generally supported when visually plotting the data, and no discernible difference in variance or other model parameters is significantly apparent when comparing these SNP-CpG pairs to other tested pairs, beyond a lower average methylation level (**Figures S20-24**). Variance in methylation level was calculated using the 'var' function in R for each CpG. This is true for both IMAGE and PQlseq, which both employ penalized quasi likelihood (PQL) as the underlying modeling framework. These sites were included in any total counts and enrichment analyses, under the assumption they still represent putative true associations between genotype and methylation. However, they were not included in the PVE estimates, as in IMAGE this results in an inflation of sites where genotype explains 100% of variance (as the numerator and denominator are identical if sigma^2 is 0), and in PQLseq it results in an inflation of larger PVE estimates for all covariates, as the denominator is smaller when sigma^2 is 0. 151,526 significant SNP-CpG pairs were removed for IMAGE PVE estimates, and 183,373 total SNP-CpG pairs for PQLseq (91,658 of which were significant). All PVE plots including these sites show similar patterns to those in the main text (**Figures S25-26).** There are two potential explanations, one is that our CpGs are very close to the SNPs, and therefore most genetic effect sizes were very large (essentially explaining 100% of the variation). More conservatively, another reason is that most methylated reads were zero across samples for a given SNP-CpG pair, and IMAGE could not obtain an accurate estimate of the sigma^2 parameter. Given the lower average methylation levels for sigma^2 = 0 sites, the latter seems likely.

MOLECULAR ECOLOGY

S3. Supplemental Figures

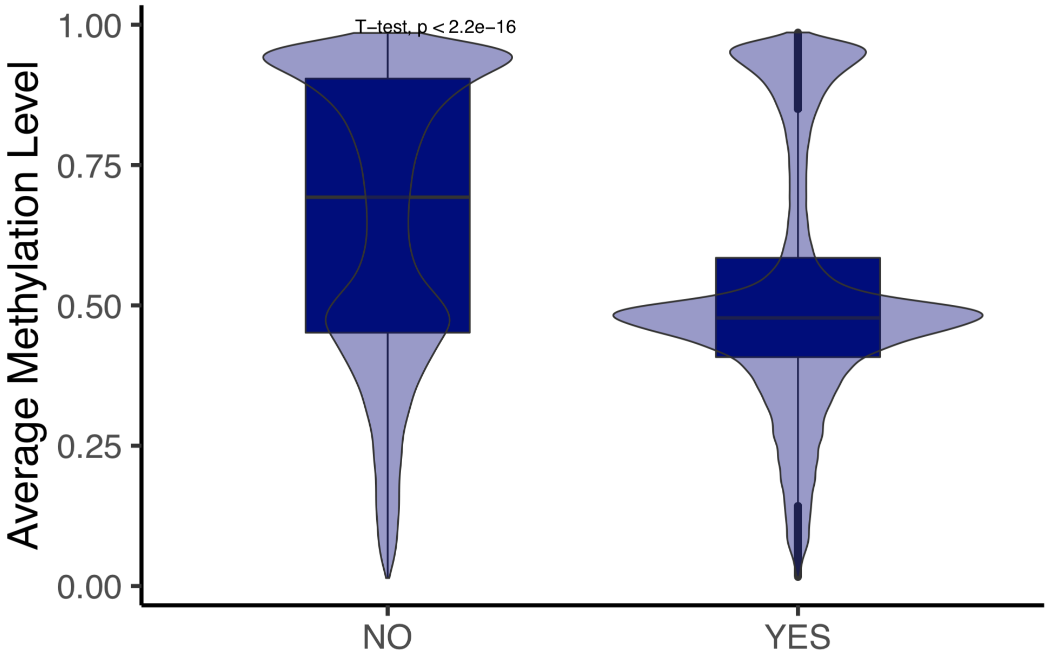

Supplemental Figure 20. Average methylation level of CpGs for SNP-CpGs pairs where the estimate of sigma squared was zero. $Sigma^2 = 0$ SNP-CpG pairs ("YES") show lower average methylation levels.

MOLECULAR ECOLOGY

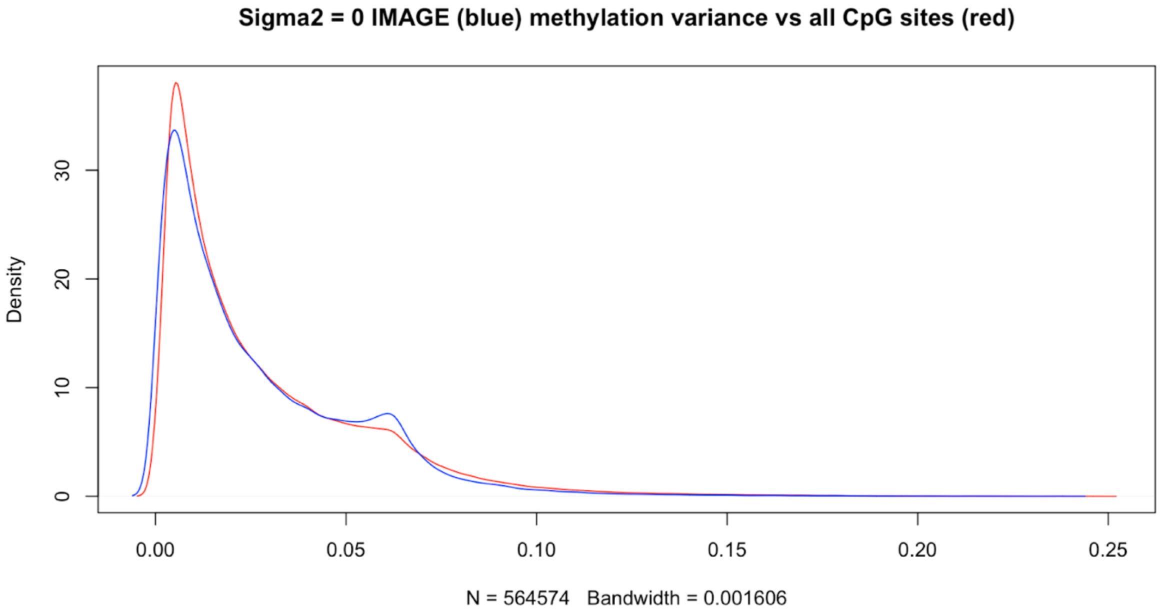

Supplemental Figure 21. Density plot of methylation variance of CpGs for SNP-CpGs pairs where the estimate of sigma squared was called as zero (blue). Sigma^2= 0 SNP-CpGs pairs do not show lower average methylation variation.

MOLECULAR ECOLOGY

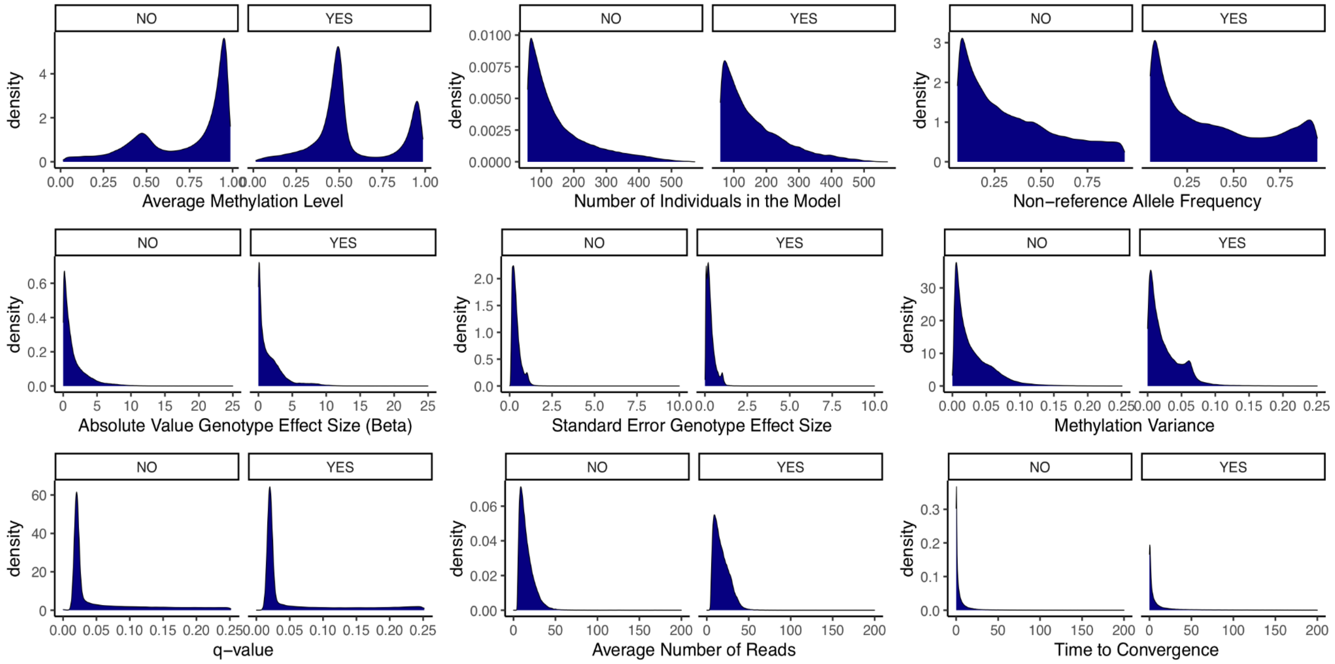

Supplemental Figure 22. Characteristics of Sigma^2 = Zero pairs for all tested CpGs. Comparisons between Sigma^2> 0 (NO) and Sigma^2 = 0 (YES) CpGs.

MOLECULAR ECOLOGY

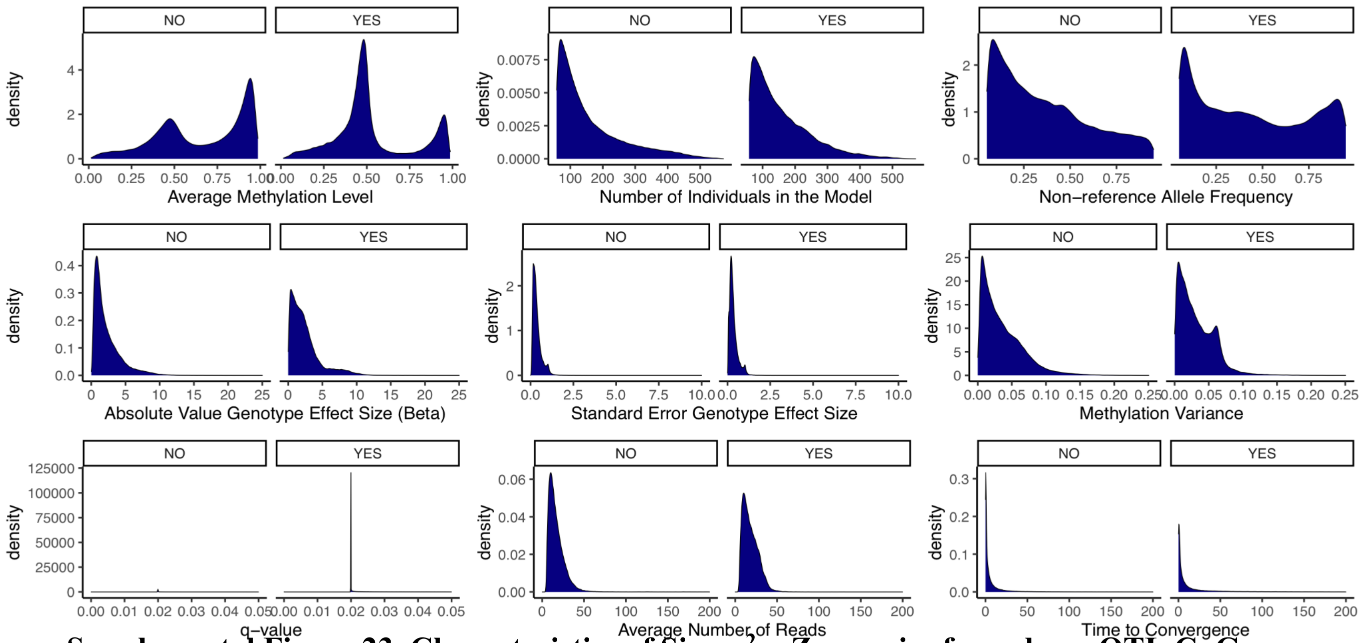

Supplemental Figure 23. Characteristics of Sigma^2 = Zero pairs for only meQTL CpGs. Comparisons between Sigma$^2 > 0$ (NO) and Sigma$^2 = 0$ (YES) CpGs.

MOLECULAR ECOLOGY

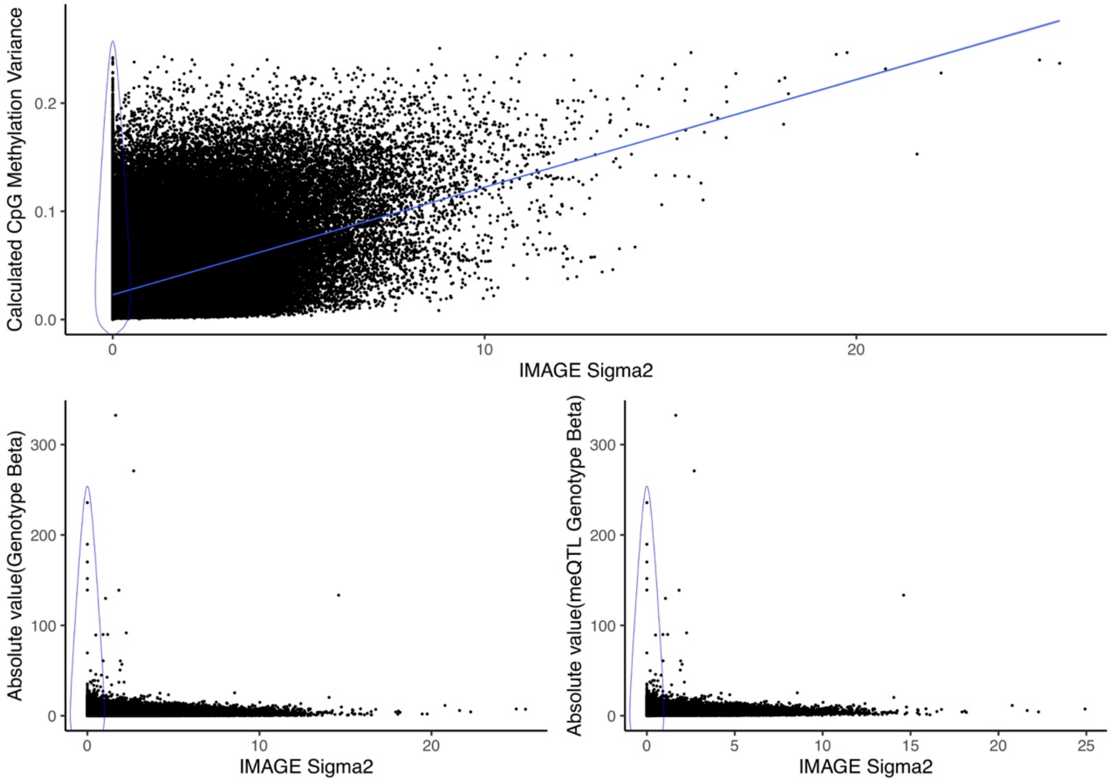

Supplemental Figure 24. Sigma^2 of zero CpGs show a range of methylation variance and normal genetic effect sizes. Blue circles highlight SNP-CpGs where sigma^2 equals 0. **(1)** Relationship between methylation variance of each CpG against sigma squared value for the CpG in the IMAGE model. **(2).** Relationship between sigma squared and the absolute value of the SNP effect size in IMAGE for all tested pairs (*left*) and for meQTL (*right*). There are a few cases where a sigma squared of zero corresponds to a large effect size, but in general the effect sizes are still quite small.

MOLECULAR ECOLOGY

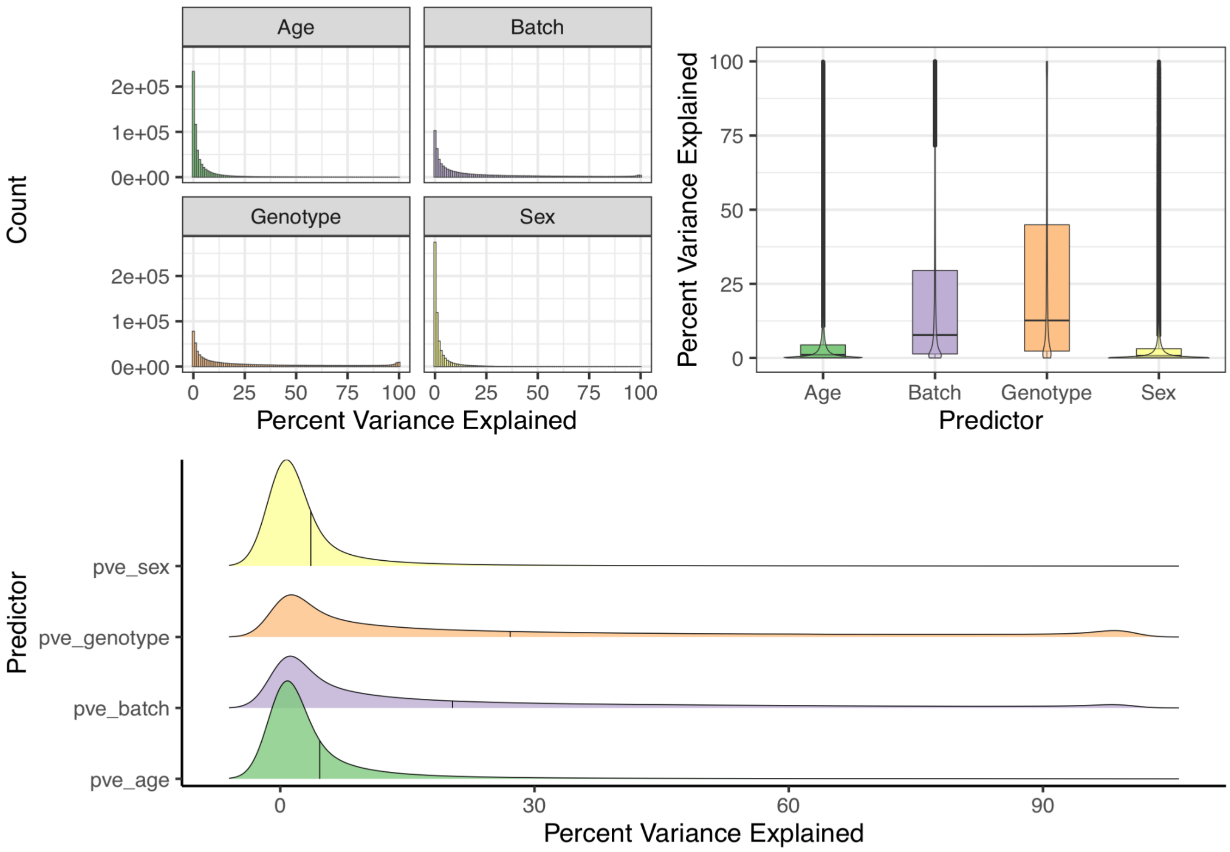

Supplemental Figure 25. Percent Variance Explained (PVE) plots with all sites (no filtering).

MOLECULAR ECOLOGY

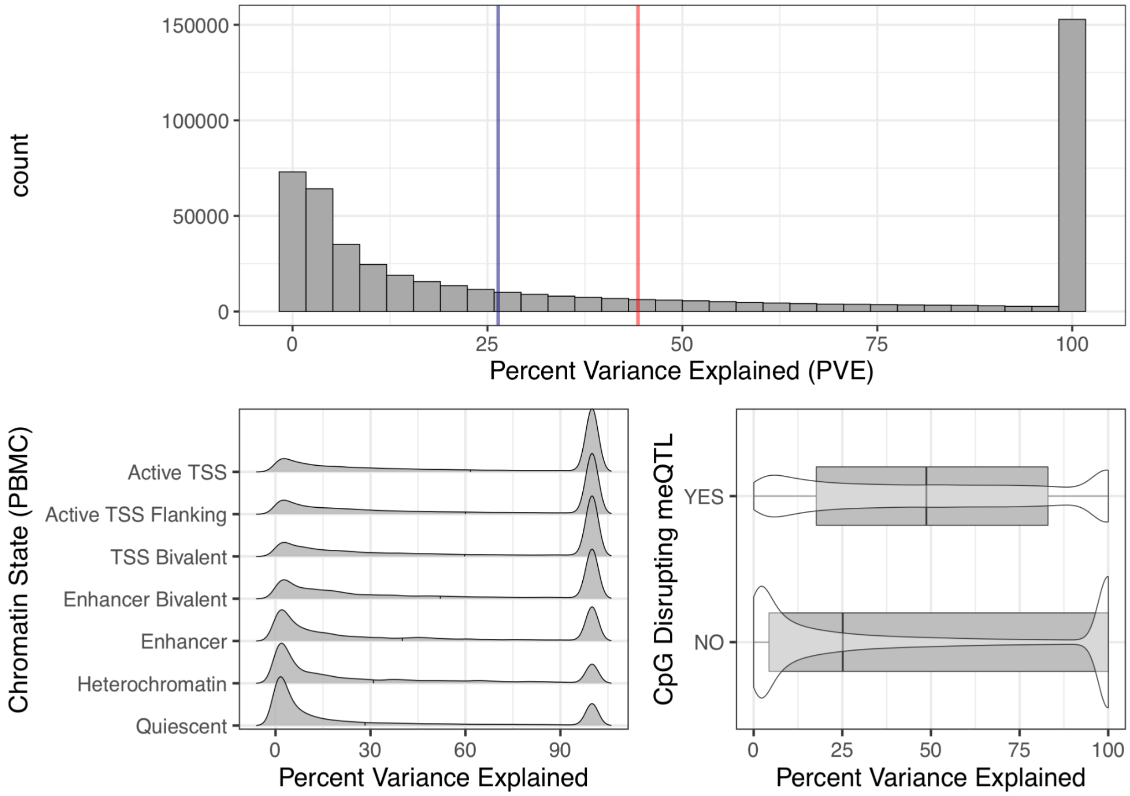

Supplemental Figure 26. PVE plots with all sites (no filtering).

MOLECULAR ECOLOGY

S4. eQTL Analysis
Supplemental Methods

4.1 Modeling: GEMMA eQTL (Linear Mixed Model); Zhou & Stephens, 2012

Gene expression residuals ~ genotype at associated SNP + sex + age + RIN +(1|relatedness matrix (GRM from WGS SNP data))

4.2 eQTL modeling without repeated samples (unique individuals)

Removing repeated expression measures in the eQTL analysis did not significantly alter effect size estimates, although we see a decrease in significance, as expected. The original models account for these replicates with the relatedness matrix included as a random effect. We therefore present the original GEMMA results (n=172 samples) in the paper.

We ran the eQTL analysis in GEMMA without replicates (n=120 individuals) and found 34.98% of SNP-Gene pairs remain significant (20% FDR). 7,901 significant SNP-gene pairs were identified in total (20% FDR), 81.14% (n=6,411) of which were also significant in the model with repeated measures. The betas for SNP-gene pairs modeled in both are highly correlated (n= 4,780,850, rho= 0.814, p-value < 2.2e-16). This increased when limiting to sites that were significant eQTL in the original analysis (20% FDR, n=18,327, rho = 0.976, p-value < 2.2e-16), and further when looking at sites significant (20% FDR) in both models (n=6,411, rho = 0.981, p-value < 2.2e-16). (**Figure S31**).

4.3 Permutations on chromosome 1 for eQTL analysis

Expression residuals were permuted and input with genotype data to GEMMA (n = 7,550 total permutations, 10 per gene) for chromosome 1. The Q-Q plot (**Figure S29**) shows a graphical representation of the deviation of the observed chromosome 1 *p* values from the null hypothesis (permuted values), and an enrichment of significant values. The empirical p-value distribution also appeared uniform (**Figure S29**), unlike in the IMAGE analysis. We therefore used the R package 'qvalue' to perform multiple testing correction and provide significance levels.

MOLECULAR ECOLOGY

4.4 meQTL-eQTL overlap analyses

meQTL SNPs were not more likely to be an eQTL compared to unique non-meQTL SNPs (Fisher's exact p-value =0.3, log2OR=0.094). i.e., 104 non-meQTL (tested in IMAGE FDR > 0.05) were also an eQTL. 73.44% of those non-meQTL associated eGenes are also associated with a significant meQTL SNP (47 of the 129 meQTL-eQTL eGenes), meaning eGenes associated with non-meQTL might just be genes with a lot of nearby regulatory sequence variation. There are 89 meQTL-eQTL eGenes that do not have an associated non-meQTL (FDR > 0.05) SNP. This may be more conservative because it is then less likely there is an overlap because these are just very variable regions, e.g., this removes genes like *MAMU-E*. A GO enrichment for those 89 compared to the 6,100 with a tested meQTL, revealed even more significant (FDR 10%) enrichment including: response to interferon-gamma, extracellular space, response to external stimulus, immune response, immune system processes, interspecies interactions, defense response to other organism, phagocytic vesicle, and vesicles in general (Supplemental Tables File 2 (STables2.4).

The vertebrate TF motif *PRDM14* was enriched near meQTL-eQTL SNPs compared to non-eQTL meQTL (FDR 10%; **S4. Table 1**). meQTL that are also significant eQTL have a higher minor allele frequency (MAF) on average (t.test; p-value =0.015). meQTL-eQTL CpGs are not more likely to be in a DMR between species than non-eQTL meQTL CpGs; they are slightly depleted compared to non-eQTL meQTL CpGs (Fisher's exact test, log2OR=-0.633, p-value=1.37E-06). 31 meQTL-eQTL eGenes overlap genes showing differential methylation and expression between macaque species (STables2.7) in Wang *et al*., 2023, but not more than expected given the overlap in the background set of tested genes (Fisher's exact test, log2OR=0.3199, p-value=0.3188).

MOLECULAR ECOLOGY

S4. Supplemental Figures

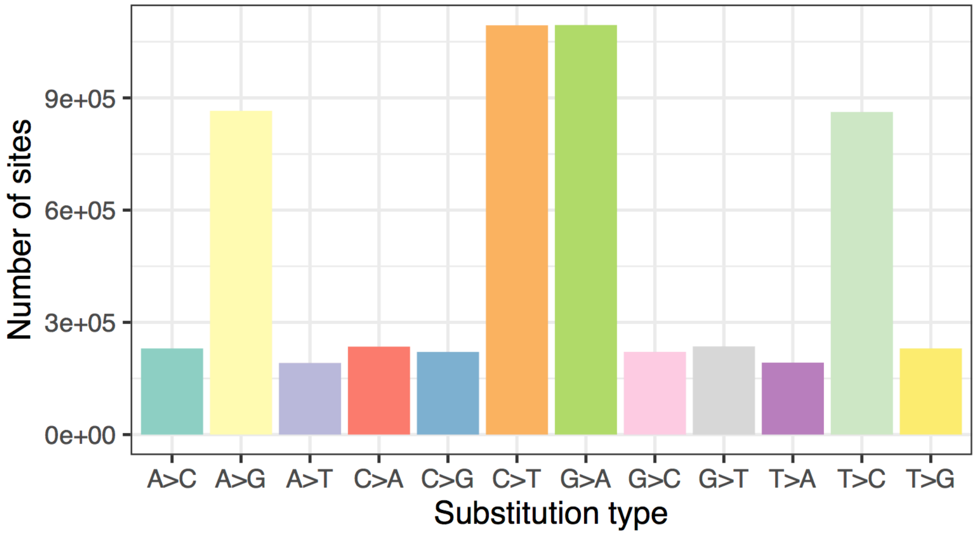

Transitions (TS)	Transversions (TV)	TS/TV
3,916,619	1,757,172	2.23

Supplemental Figure 27. Whole genome sequencing imputed VCF description. VCF is filtered for genotype probability > 0.9, MAF > 0.05, and HWE 10e-8. **(1)** Counts of substitution type (total SNPs = 5,684,471). **(2)** Table with TS/TV ratio.

MOLECULAR ECOLOGY

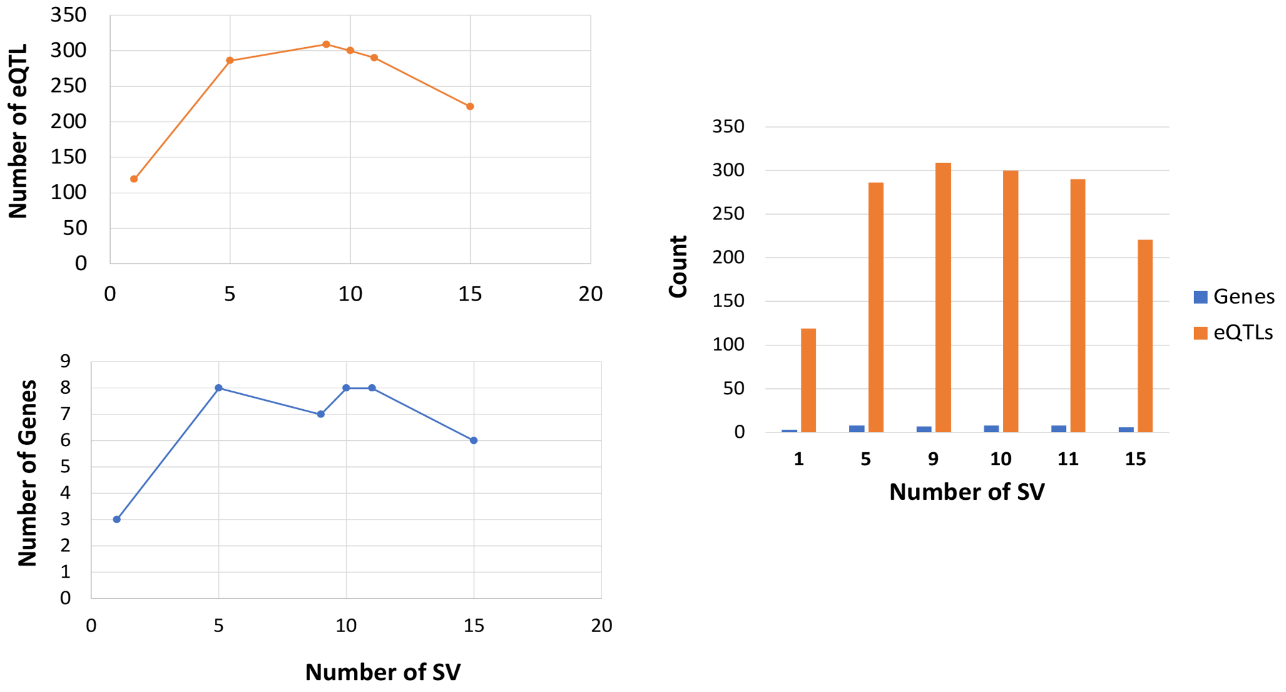

Supplemental Figure 28. Surrogate Variable Analysis (SVA). GEMMA eQTL and eGene detection on chromosome 1 for normalized expression counts with 1,5,9,10,11, and 15 surrogate variables (SVs) regressed out. The greatest increase between SVs was seen from 1 to 5, with additional variables not providing significant increases in eQTL and eGene detection.

MOLECULAR ECOLOGY

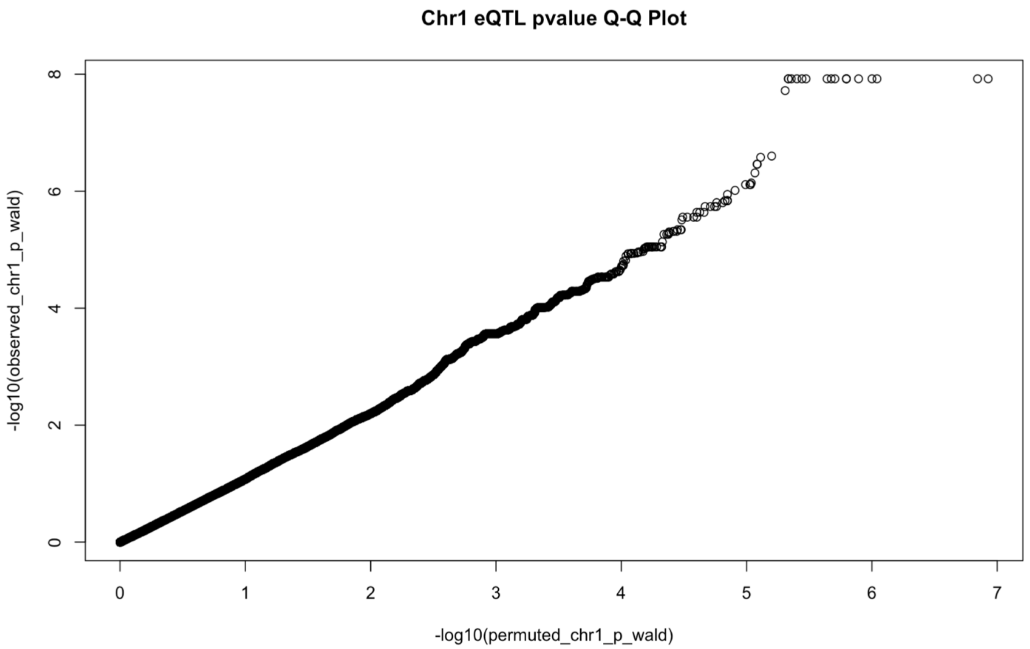

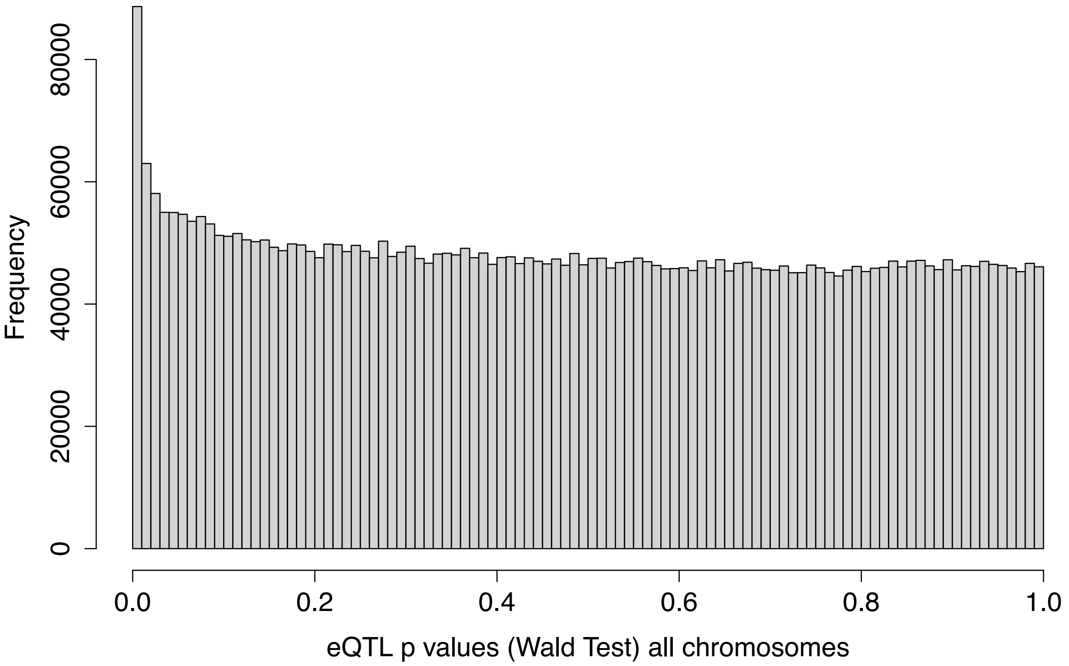

Supplemental Figure 29. GEMMA eQTL modeling. (1). Q-Q plot of permutations done for eQTL analysis for chromosome 1 **(2).** Empirical p-value distribution GEMMA, all chromosomes.

MOLECULAR ECOLOGY

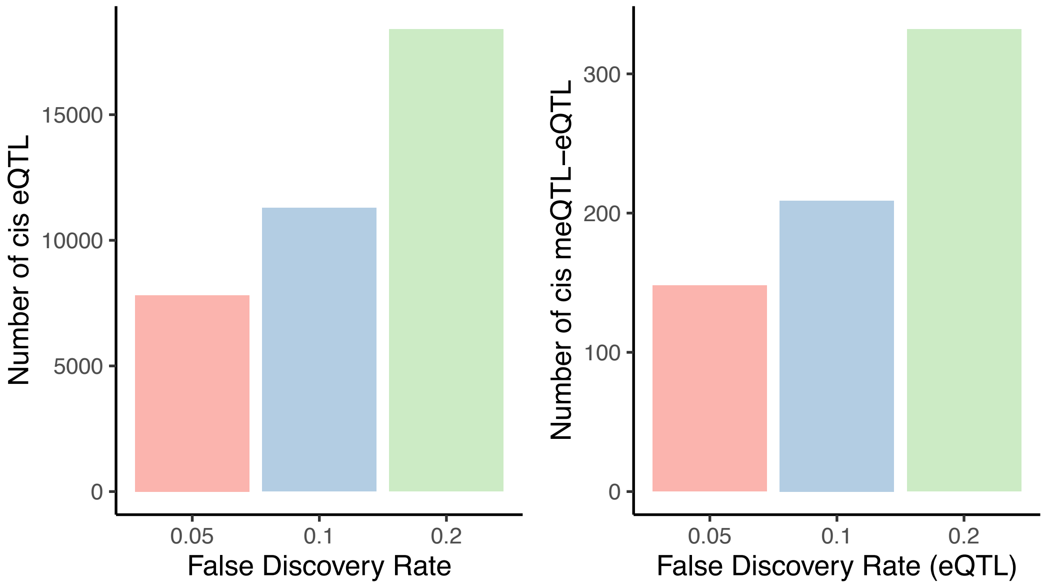

Supplemental Figure 30. Whole blood eQTL FDR thresholds. (1). Number of *cis* eQTL identified at different q-value thresholds (0.05 = 7,811; 0.1=11,287; 0.2=18,398). **(2).** Number of overlapping *cis* meQTL-eQTL identified at different q-value thresholds (0.05= 148, 0.1= 209, 0.2= 332).

MOLECULAR ECOLOGY

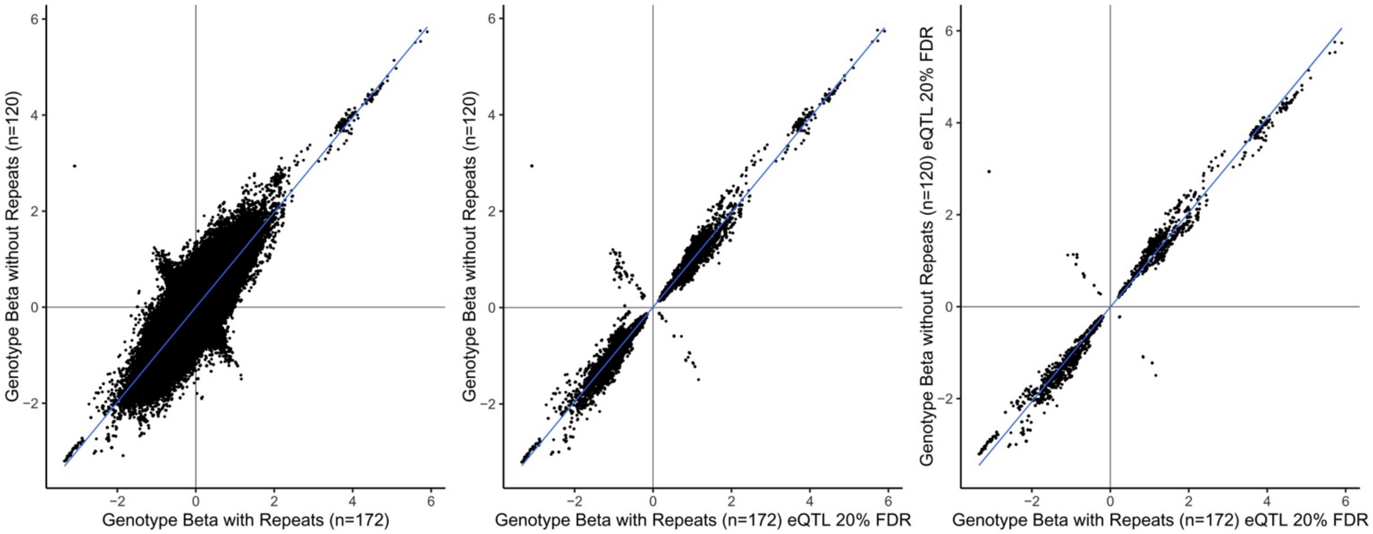

Supplemental Figure 31. Effect size correlation between SNP genotype-gene expression models in GEMMA with and without replicates. x-axis: GEMMA genotype effect size including repeated individuals (n=172), y-axis: GEMMA genotype effect size with only unique individuals (n=120). Line represents linear regression line ('lm' function). **(1).** Betas for all modeled SNP-gene pairs, **(2).** Betas for significant eQTL SNP-gene pairs (20% FDR) in complete model, **(3).** Betas for significant eQTL SNP-gene pairs (20% FDR) in both models.

MOLECULAR ECOLOGY

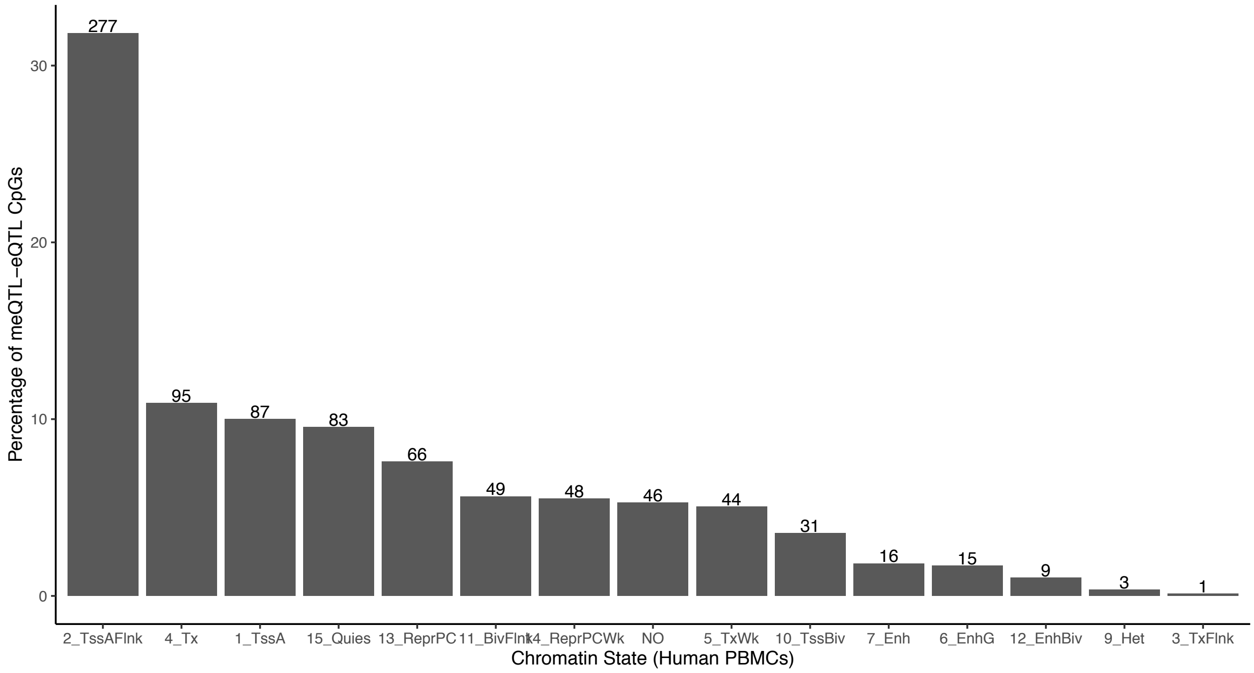

Supplemental Figure 32. Proportion of meQTL-eQTL CpGs in different chromatin states. Counts represent the number of unique CpGs in that state.

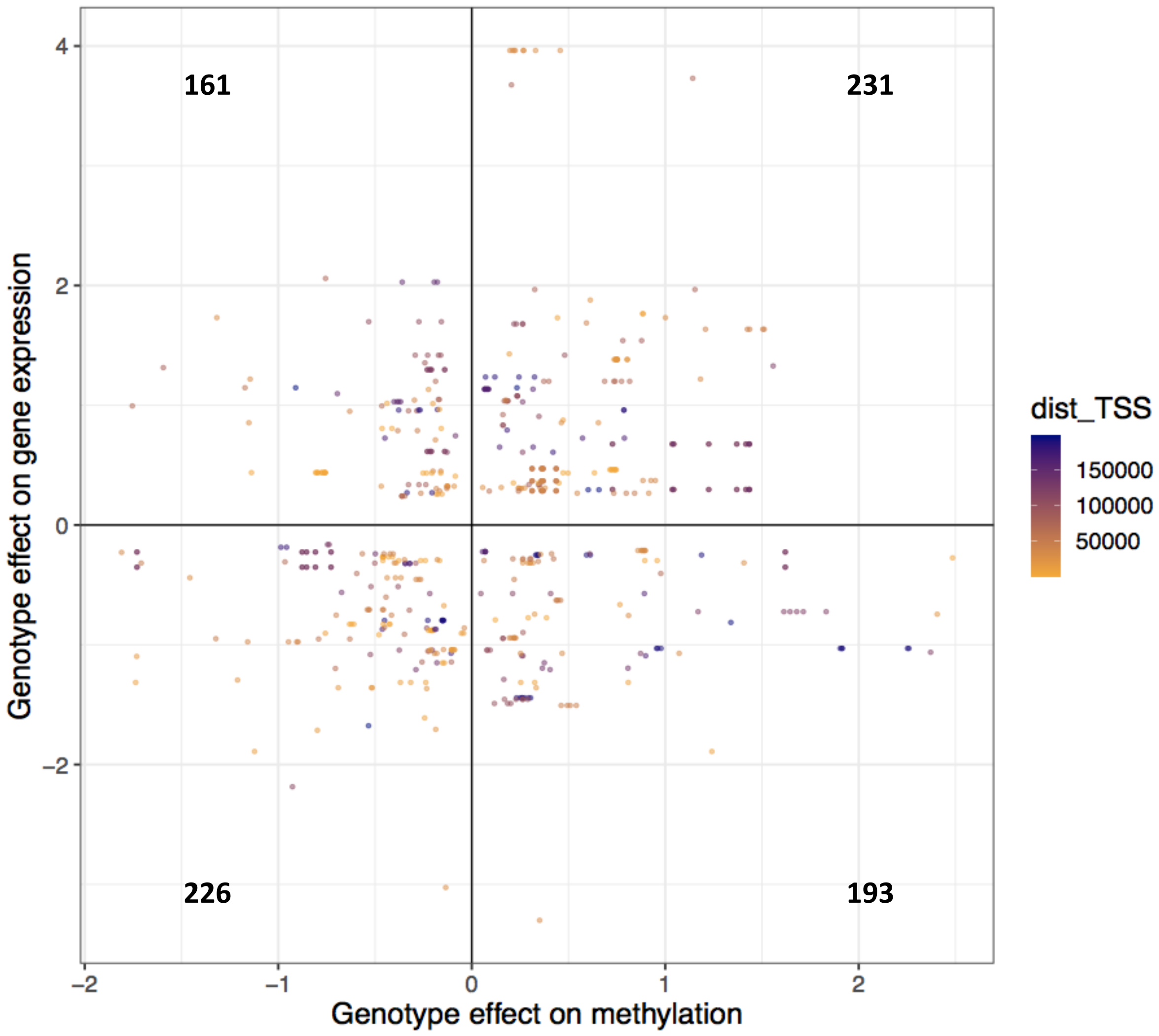

Supplemental Figure 33. meQTL-eQTL SNP-CpG-Gene trios. Quadrant plot of genotype effect on expression (GEMMA) and methylation (PQLseq). PQLseq betas are used as it models the same effect allele as GEMMA (minor allele as effect allele). Direction of effect of QTL genotype on methylation (x-axis) and expression levels (y-axis). Colors represent the distance from the meQTL-eQTL to the TSS of the eGene. Numbers represent the count of meQTL-eQTL in each quadrant.

MOLECULAR ECOLOGY

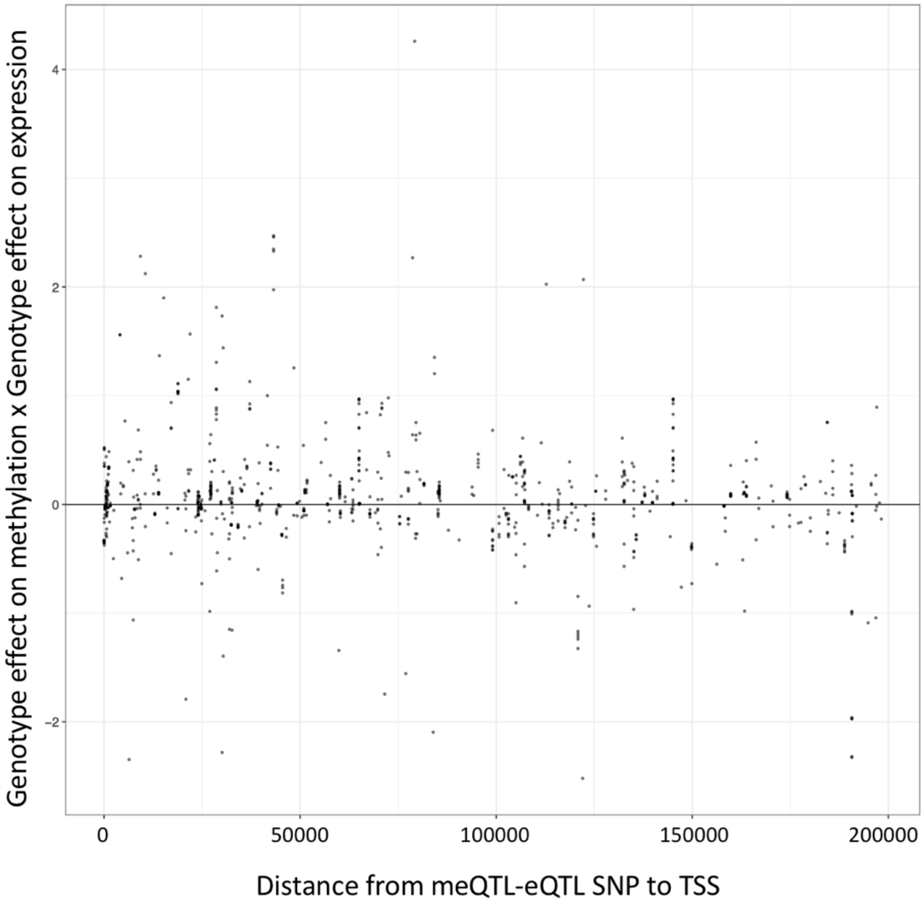

Supplemental Figure 34. There does not appear to be a relationship between direction of effect on expression and methylation and distance to TSS (bp). i.e., y-axis negative values mean genotype effect on expression and methylation is in the opposite direction, which we would hypothesize would be more common in promoter and proximal regulatory regions of genes (within 2 to 20kb of the TSS), where methylation and expression levels are generally negatively correlated.

MOLECULAR ECOLOGY

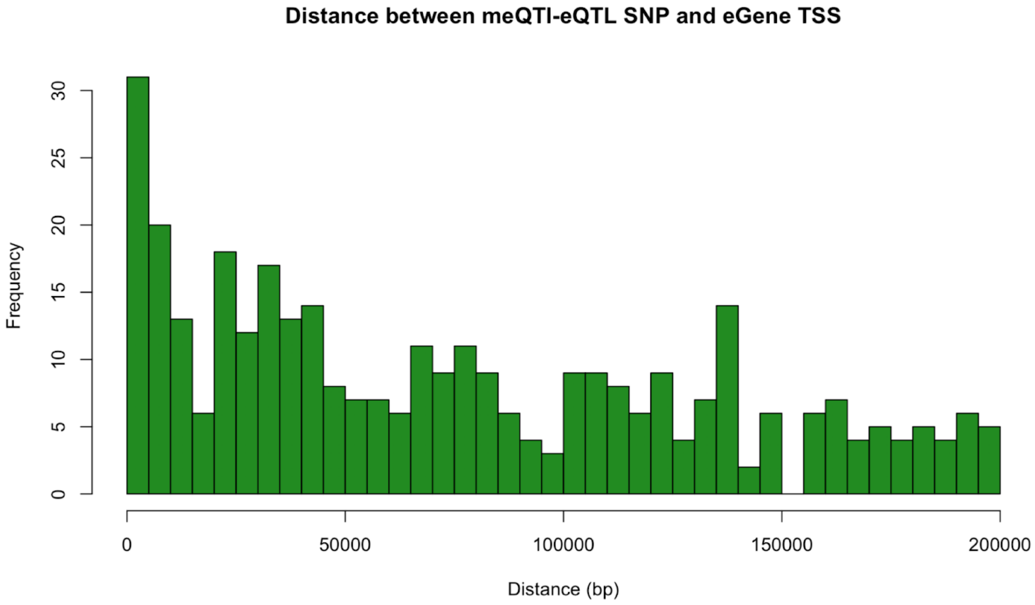

Supplemental Figure 35. Distance (bp) between the meQTL-eQTL and the TSS of the associated eGene. These loci were 74,700 bp away on average from the TSS of the gene (median: 65,018 bp, SD: 58,031.97).

MOLECULAR ECOLOGY

Known					
**Motif**	**Family**	**q-value**	**Percent Target**	**Percent Background**	**Percent Difference**
PRDM14	ZF	0.0948	5.42	2.05	3.37

Supplemental Table 1. meQTL-eQTL TF motif enrichment results. Input sequence = 332, background = 25,964. Known vertebrate TF motifs with q-value < 0.10, > 5% of target sequences with motif, and >1% difference between target and background sequence.

S5. Correlation Analysis
Supplemental Methods

5.1 Genotype effect on methylation and expression and CpG-gene expression correlation

Average distance between meQTL and eGene TSS for correlated pairs was 97,945.61 bp (median=102,060. SD=64,038.13). meQTL-eQTL CpGs with significant methylation-expression correlations are more variable on average than non-correlated CpGs (t.test; p-value = 0.021). PQLseq and GEMMA share comparable beta estimates (minor allele as the effect allele). For the 29 meQTL-eQTL CpG-Gene pairs with PQLseq estimates, 20 regulatory QTL have an effect on methylation and expression in the same direction, 9 have an effect on methylation and expression in the opposite direction. This agrees with the correlation estimates, e.g., QTL increases methylation, decreases expression, and this CpG-Gene pair shows a negative relationship, in some cases, but not all (**Figure S39**). 6 correlated methylation-expression eGenes (37.5%) were in the list of differentially methylated and expressed genes between macaque species, but not enriched compared to all meQTL-eQTL eGenes (log2OR=1.07, p-value=0.2124).

MOLECULAR ECOLOGY

S5. Supplemental Figures

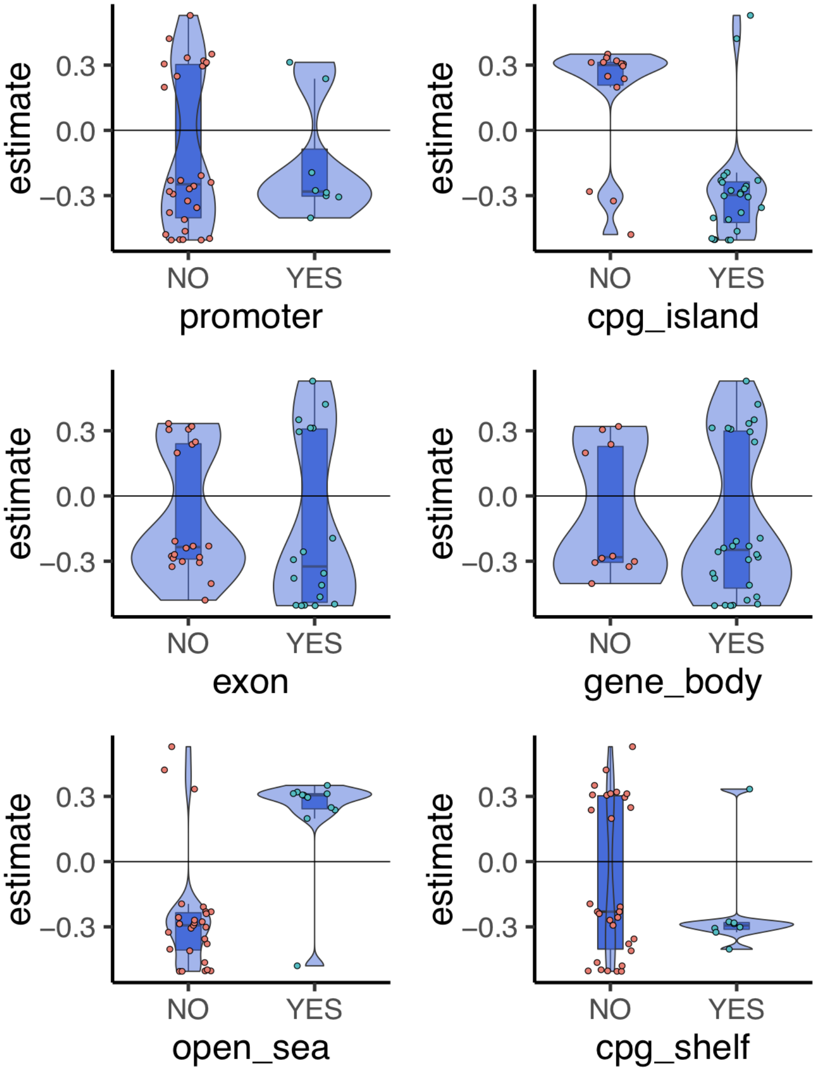

Supplemental Figure 36. meQTL-eQTL methylation-expression Spearman's correlation estimate (FDR 20%) by gene features. Each dot represents an estimate for a CpG-Gene pair**.** Macaque promoter regions = 2000 bp upstream of TSS.

MOLECULAR ECOLOGY

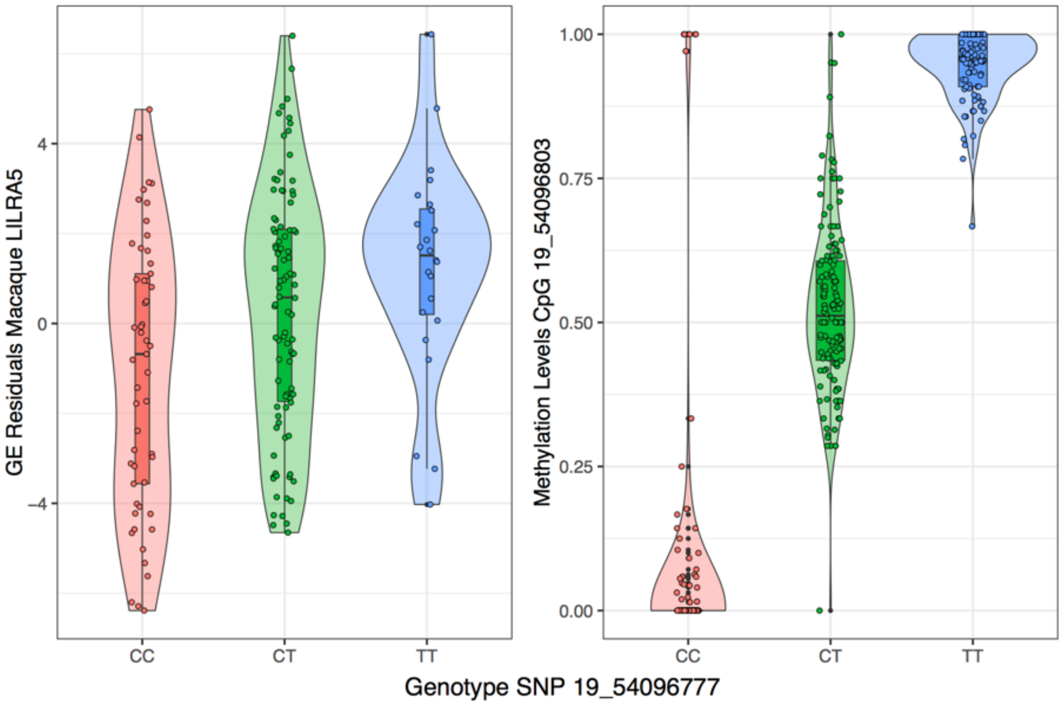

Supplemental Figure 37. meQTL-eQTL with correlated CpG methylation- eGene expression. eQTL for LILRA5 (*left*) and meQTL (*right*).

MOLECULAR ECOLOGY

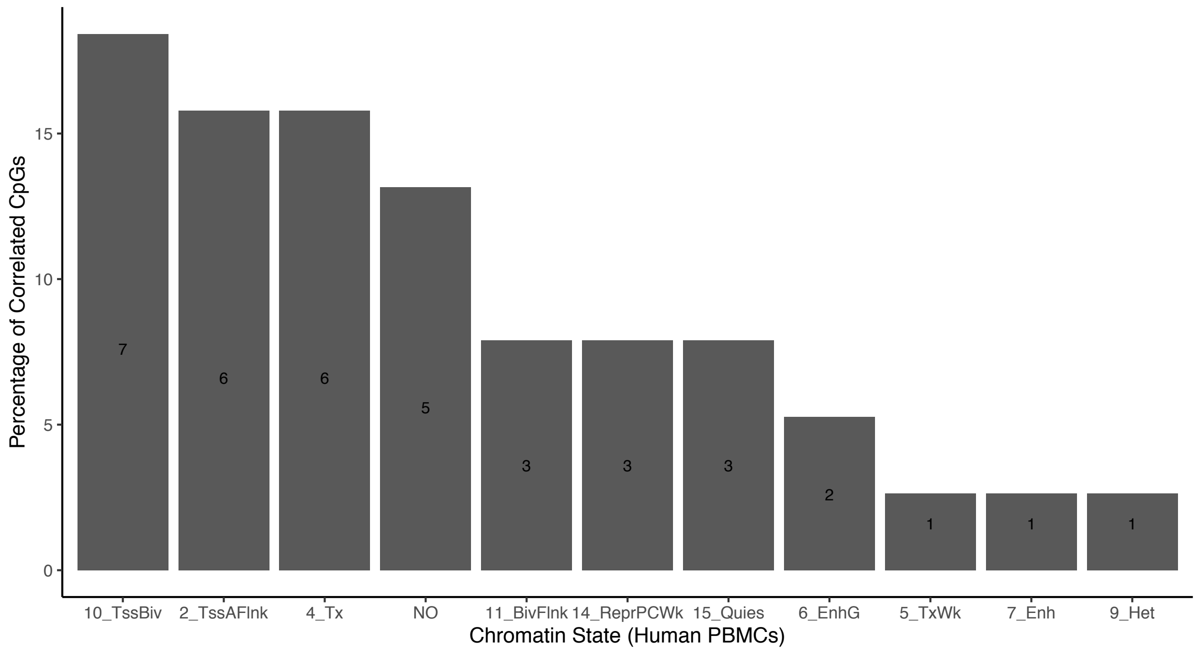

Supplemental Figure 38. Proportion of CpGs correlated with eGene expression found in different chromatin states. Numbers represent counts of correlated CpG-gene pairs. "NO" means no chromatin state annotation.

MOLECULAR ECOLOGY

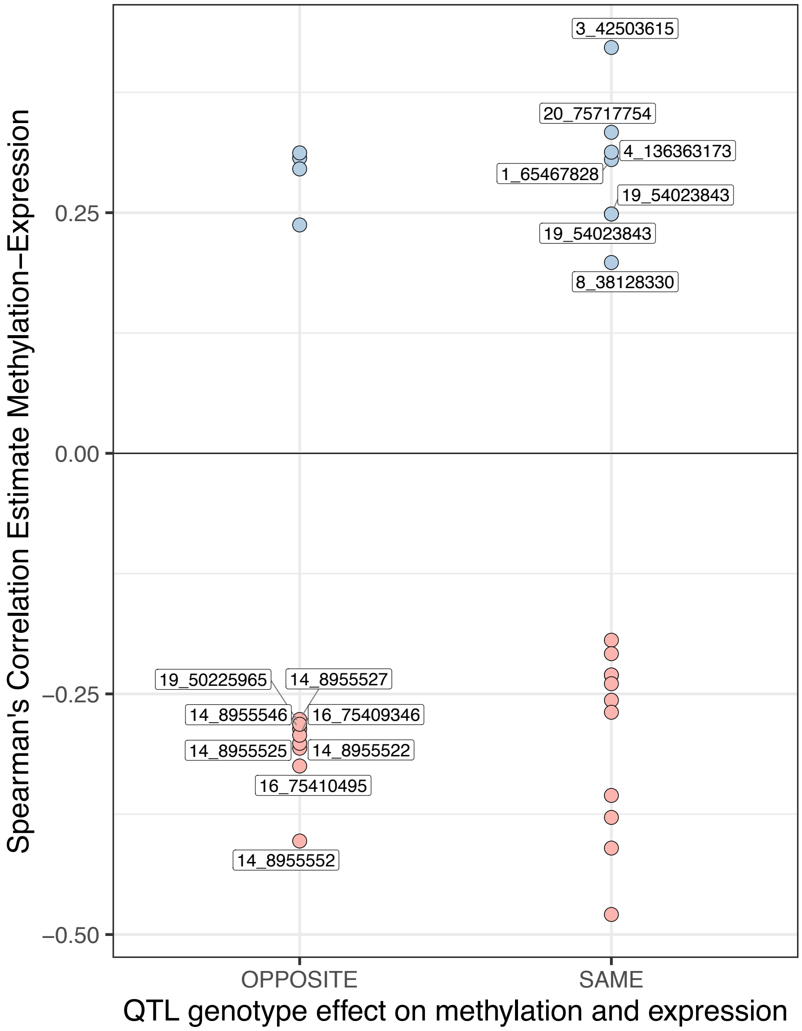

Supplemental Figure 39. QTL genotype effect on methylation and expression and methylation-expression correlation. N = 20 with QTL having an effect on methylation and expression in the same direction. N = 9 having an effect on methylation and expression in the opposite direction. This only agrees with the correlation estimates in some cases (top right, bottom left). This uses PQLseq betas, with only 29 of the 38 CpGs having model outputs. Labels are CpG sites where genotype effect agrees with Spearman's correlation estimate direction.

MOLECULAR ECOLOGY

S6. Baboon and wolf meQTL CpG enrichment
Supplemental Methods

6.1 Baboon and wolf meQTL annotations

meQTL annotation and enrichment analyses were also carried out on the publicly available baboon and grey wolf *cis* meQTL dataset from Fan *et al*., 2019. This dataset was generated using the same IMAGE pipeline, and therefore is ideal for comparing patterns of genotype-dependent DNA methylation enrichment across species. Gene feature annotations were downloaded from Ensembl, and CpG island annotations from UCSC Genome Browser (*Panu2.0; canFam3.1)*. For baboons only (due to evolutionary distance between humans and wolves), human chromatin state annotations were converted to baboon genome coordinates (*Panu2.0)* using LiftOver. 93.75% of records were successfully converted (323,539 converted; 20,248 failed). One sided fisher's exact was used to test for enrichment or depletion. Annotation and enrichment results in **SFigures 40-44** and Supplemental Tables File 1 (STables1.15).

MOLECULAR ECOLOGY

S6. Supplemental Figures

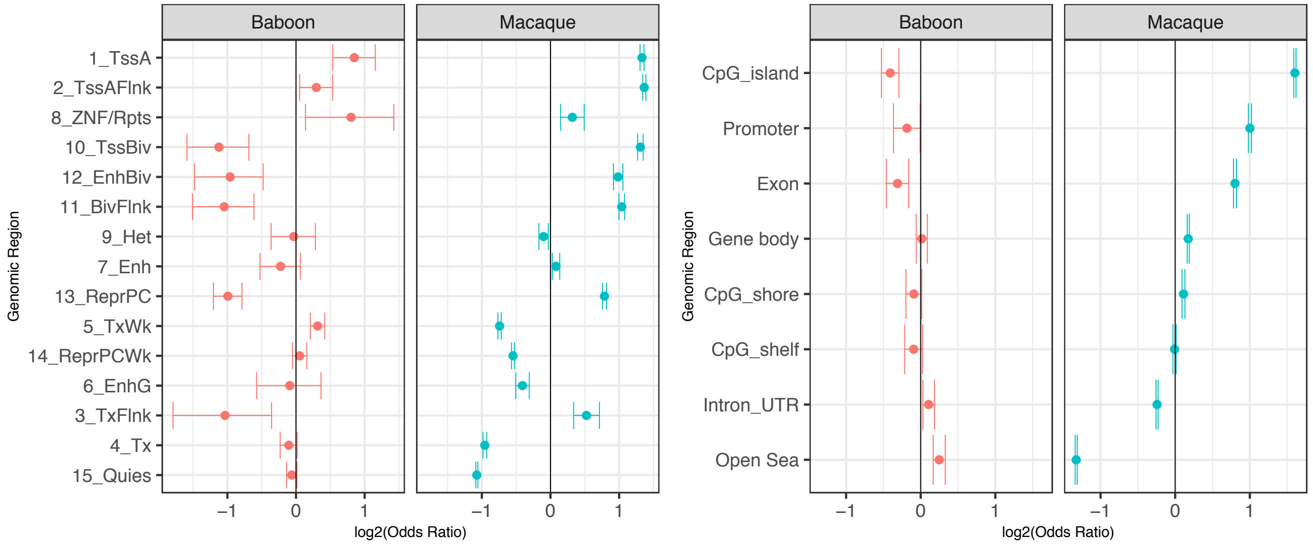

Supplemental Figure 40. Gene feature and chromatin state enrichments for published baboon meQTL versus macaque meQTL CpGs. Chromatin states are human PBMC annotations converted to baboon and macaque genome coordinates.

MOLECULAR ECOLOGY

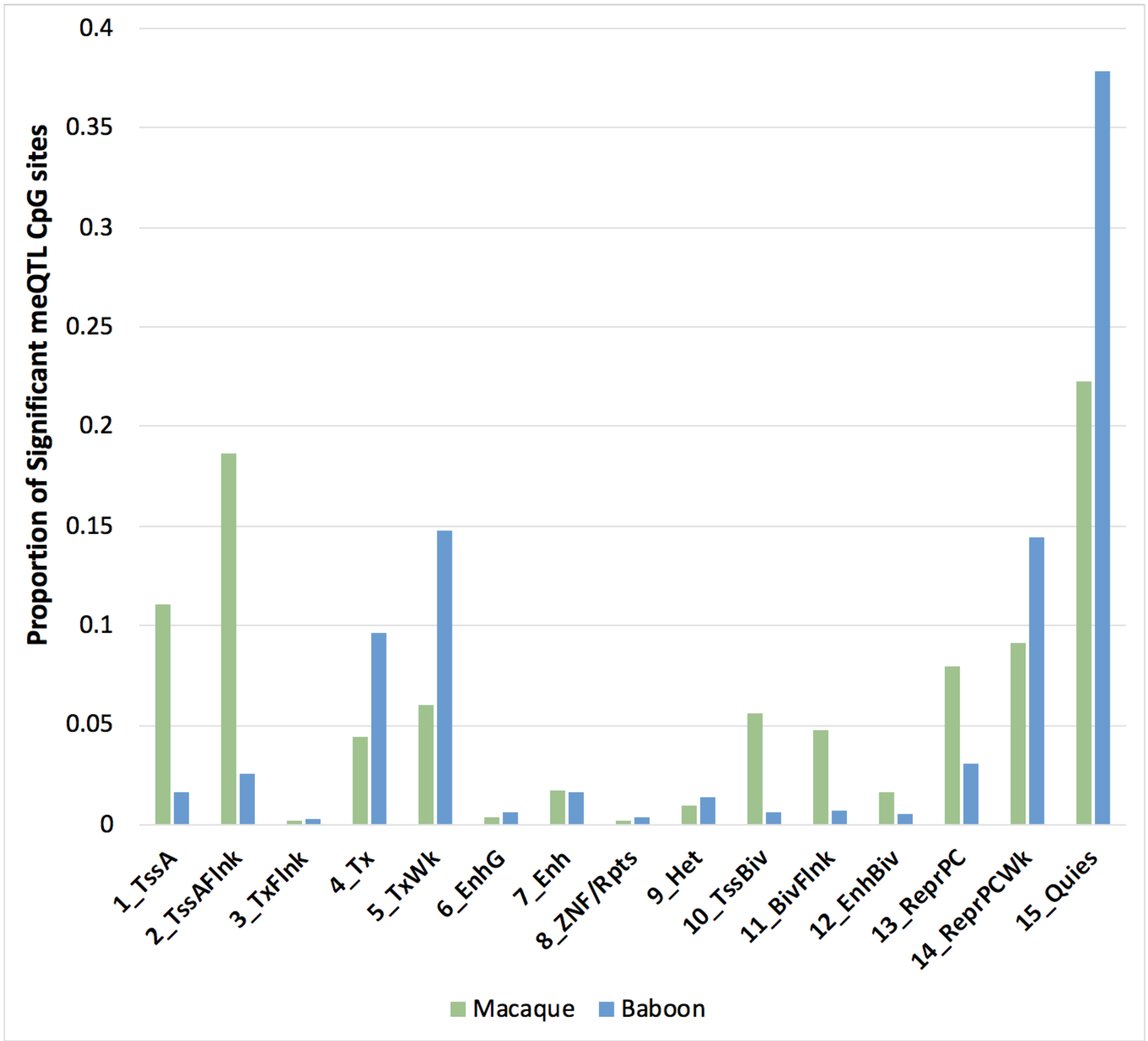

Supplemental Figure 41. Proportion of meQTL CpGs found in different human PBMC chromatin states by species.

MOLECULAR ECOLOGY

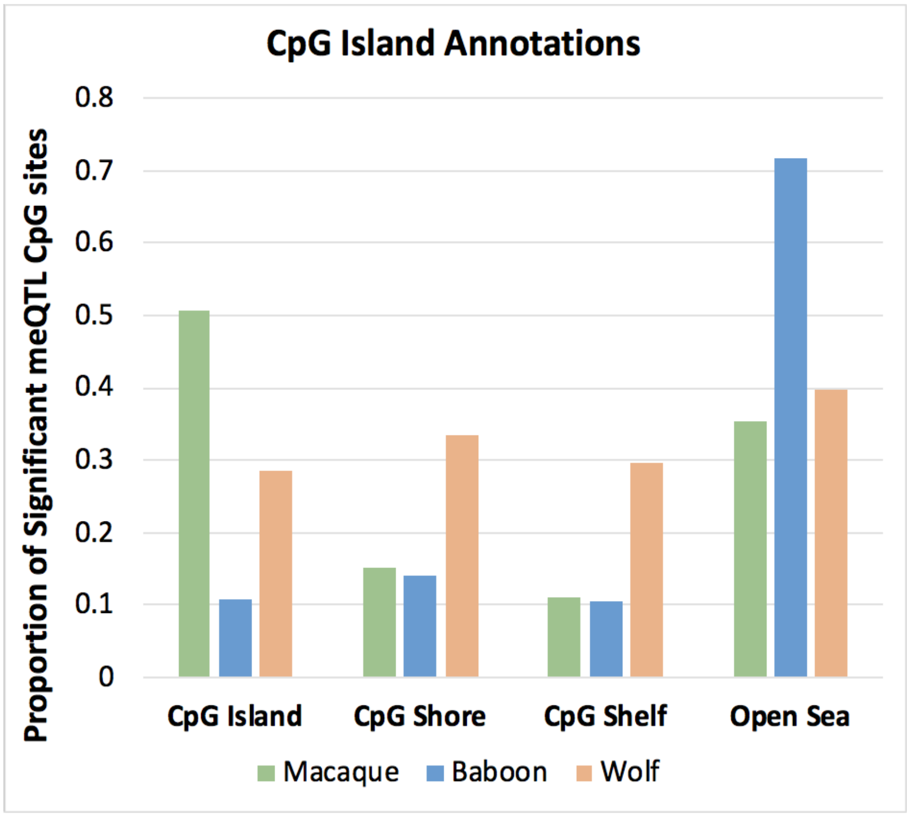

Supplemental Figure 42. Proportion of meQTL CpGs in different CpG contexts by species.

MOLECULAR ECOLOGY

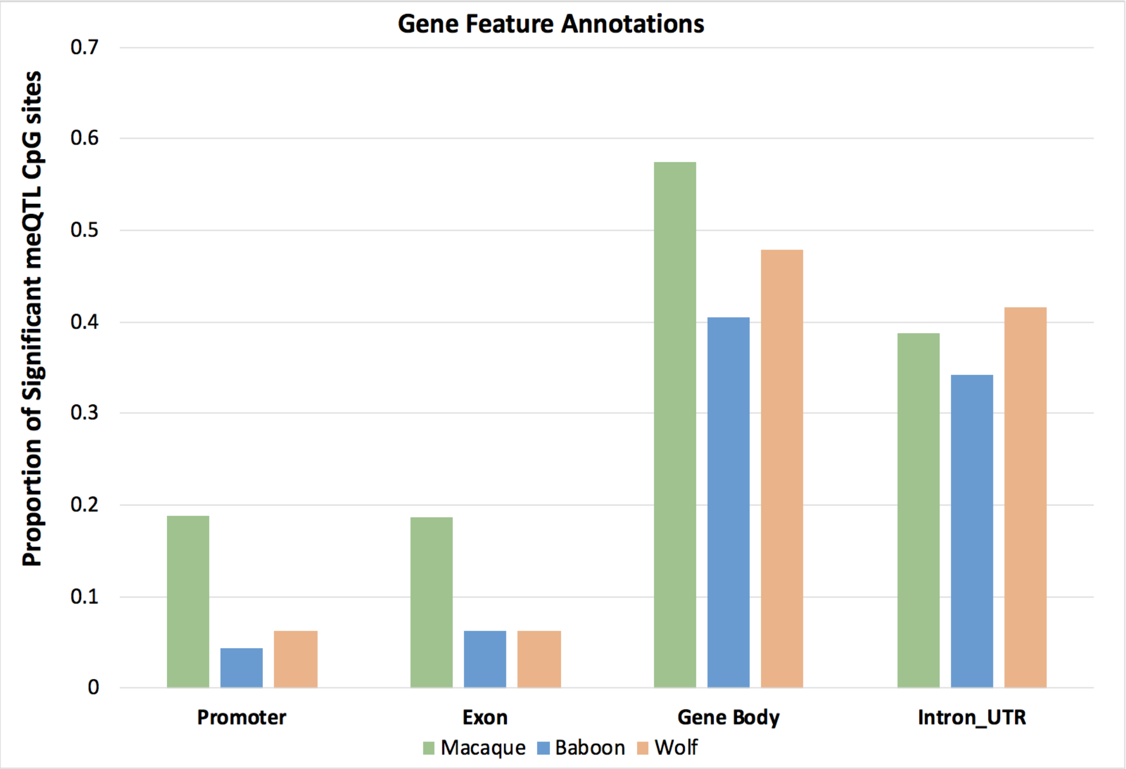

Supplemental Figure 43. Proportion of meQTL CpGs in different gene features by species.

MOLECULAR ECOLOGY

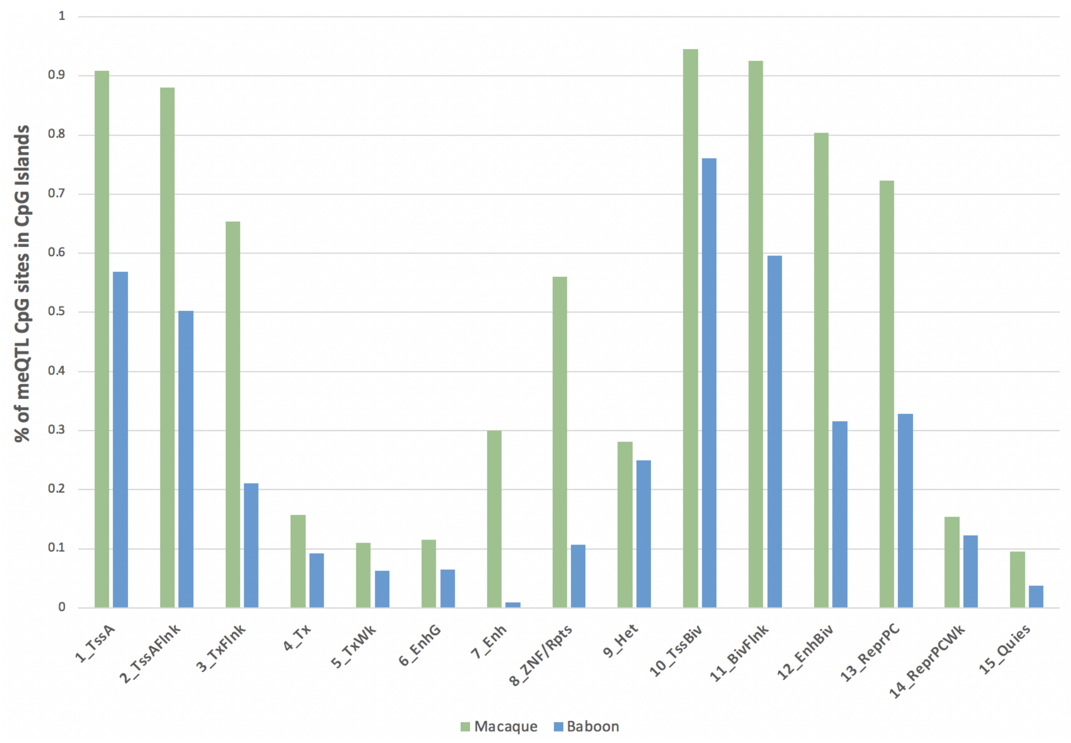

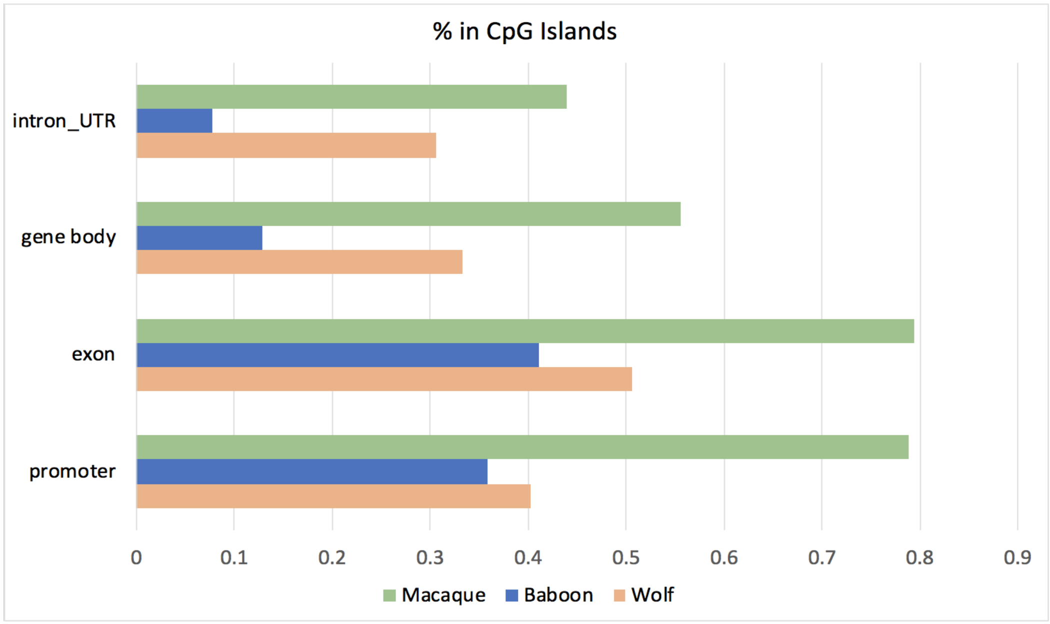

Supplemental Figure 44. Percent of meQTL CpGs in different chromatin states and genomic regions that overlap a CpG island by species. (1) Percent of baboon and macaque CpGs by chromatin state that are also in a CpG island. **(2)** Percent baboon, macaque, and wolf CpGs by genomic region that are also in a CpG island. The macaque consistently shows a larger proportion of CpGs falling in CpG islands regardless of region. CpG island annotation quality may vary by species.

Supplemental References

Akdemir, D. & Okeke, U. G. EMMREML: Fitting Mixed Models with Known Covariance Structures. https://cran.r-project.org/package=EMMREML. R package version 3.1 (2015).

Cuomo, A. S. E., Alvari, G., Azodi, C. B., single-cell eQTLGen consortium, McCarthy, D. J., & Bonder, M. J. (2021). Optimizing expression quantitative trait locus mapping workflows for single-cell studies. *Genome Biology*, *22*(1), 188.

Danecek, P., Bonfield, J. K., Liddle, J., Marshall, J., Ohan, V., Pollard, M. O., Whitwham, A., Keane, T., McCarthy, S. A., Davies, R. M., & Li, H. (2021). Twelve years of SAMtools and BCFtools. *GigaScience*, *10*(2). https://doi.org/10.1093/gigascience/giab008

Fan, Y., Vilgalys, T. P., Sun, S., Peng, Q., Tung, J., & Zhou, X. (2019). IMAGE: high-powered detection of genetic effects on DNA methylation using integrated methylation QTL mapping and allele-specific analysis. *Genome Biology*, *20*(1), 220.

Godec, J., Tan, Y., Liberzon, A., Tamayo, P., Bhattacharya, S., Butte, A. J., Mesirov, J. P., & Haining, W. N. (2016). Compendium of Immune Signatures Identifies Conserved and Species-Specific Biology in Response to Inflammation. *Immunity*, *44*(1), 194–206.

Kang, H. M., Zaitlen, N. A., Wade, C. M., Kirby, A., Heckerman, D., Daly, M. J., & Eskin, E. (2008). Efficient control of population structure in model organism association mapping. *Genetics*, *178*(3), 1709–1723.

Lea, A. J., Vilgalys, T. P., Durst, P. A. P., & Tung, J. (2017). Maximizing ecological and evolutionary insight in bisulfite sequencing data sets. *Nature Ecology & Evolution*, *1*(8), 1074–1083.

Liberzon, A., Birger, C., Thorvaldsdóttir, H., Ghandi, M., Mesirov, J. P., & Tamayo, P. (2015). The Molecular Signatures Database (MSigDB) hallmark gene set collection. *Cell Systems*, *1*(6), 417–425.

Revelle, William (2023). *psych: Procedures for Psychological, Psychometric, and Personality Research*. Northwestern University, Evanston, Illinois.

Storey, J. D., & Tibshirani, R. (2003). Statistical significance for genomewide studies. *Proceedings of the National Academy of Sciences of the United States of America*, *100*(16), 9440–9445.

Subramanian, A., Tamayo, P., Mootha, V. K., Mukherjee, S., Ebert, B. L., Gillette, M. A., Paulovich, A., Pomeroy, S. L., Golub, T. R., Lander, E. S., & Mesirov, J. P. (2005). Gene set enrichment analysis: a knowledge-based approach for interpreting genome-wide expression profiles. *Proceedings of the National Academy of Sciences of the United States of America*, *102*(43), 15545–15550.

Sun, S., Zhu, J., Mozaffari, S., Ober, C., Chen, M., & Zhou, X. (2019). Heritability estimation and differential analysis of count data with generalized linear mixed models in genomic sequencing studies. *Bioinformatics*, *35*(3), 487–496.

Wang, J., Liu, X., Lan, Y., Que, T., Li, J., Yue, B., & Fan, Z. (2023). DNA methylation and transcriptome analysis reveal epigenomic differences among three macaque species. *Evolutionary Applications*. https://doi.org/10.1111/eva.13604

Zhou, X., & Stephens, M. (2012). Genome-wide efficient mixed-model analysis for association studies. *Nature Genetics*, *44*(7), 821–824.
